# Finding Nemo’s clock reveals switch from nocturnal to diurnal activity

**DOI:** 10.1038/s41598-021-86244-9

**Published:** 2021-03-24

**Authors:** Gregor Schalm, Kristina Bruns, Nina Drachenberg, Nathalie Geyer, Nicholas S. Foulkes, Cristiano Bertolucci, Gabriele Gerlach

**Affiliations:** 1 Institute of Biology and Environmental Sciences, Carl von Ossietzky University Oldenburg, Ammerländer Heerstr. 114-118, 26129 Oldenburg, Germany; 2 Institute of Biological and Chemical Systems (IBCS), Karlsruhe Institute of Technology (KIT), Hermann-von-Helmholtz-Platz 1, 76344 Eggenstein-Leopoldshafen, Germany; 3 Department of Life Sciences and Biotechnology, University of Ferrara, Via Luigi Borsari 46, 44121 Ferrara, Italy; 4 Biology and Evolution of Marine Organisms, Stazione Zoologica Anton Dohrn Napoli, Villa Comunale, 80121 Naples, Italy; 5 Helmholtz Institute for Functional Marine Biodiversity (HIFMB), Ammerländer Heerstr. 231, 26129 Oldenburg, Germany; 6 Centre of Excellence for Coral Reef Studies and School of Marine and Tropical Biology, James Cook University, Townsville, QLD 4811 Australia

**Keywords:** Animal behaviour, Animal migration, Circadian rhythms, Gene expression

## Abstract

Timing mechanisms play a key role in the biology of coral reef fish. Typically, fish larvae leave their reef after hatching, stay for a period in the open ocean before returning to the reef for settlement. During this dispersal, larvae use a time-compensated sun compass for orientation. However, the timing of settlement and how coral reef fish keep track of time via endogenous timing mechanisms is poorly understood. Here, we have studied the behavioural and genetic basis of diel rhythms in the clown anemonefish *Amphiprion ocellaris*. We document a behavioural shift from nocturnal larvae to diurnal adults, while juveniles show an intermediate pattern of activity which potentially indicates flexibility in the timing of settlement on a host anemone. qRTPCR analysis of six core circadian clock genes (*bmal1*, *clocka*, *cry1b*, *per1b*, *per2*, *per3*) reveals rhythmic gene expression patterns that are comparable in larvae and juveniles, and so do not reflect the corresponding activity changes. By establishing an embryonic cell line, we demonstrate that clown anemonefish possess an endogenous clock with similar properties to that of the zebrafish circadian clock. Furthermore, our study provides a first basis to study the multi-layered interaction of clocks from fish, anemones and their zooxanthellae endosymbionts.

## Introduction

Diel, lunar and annual rhythms in the marine environment dominate the behaviour and physiology of numerous fish species. For example, in intricate coral reef environments, diel and lunar rhythms are major determinants of the timing of spawning, hatching and dispersal for many resident species. While adult coral reef fish are mostly sedentary, larvae are highly mobile and leave their natal reef directly after hatching. The time that larvae stay in the open ocean before settlement varies between species^1^. Dispersal sets the spatial scale for population connectivity and geographic population size (reviewed by^2^) and therefore influences replenishment of fish stocks in coral reefs. While dispersal is a very important life history trait, our knowledge about what determines dispersal duration remains incomplete.

Importantly, larvae are not just transported passively through water currents. Instead, they exhibit impressive swimming abilities before settlement^3^ and can sustain speeds faster than average currents for days^4^. This may well enable them to return to their natal reef within a given time frame. Up to 60 % of new settlers of the coral reef fishes *Ostorhinchus doederleini* and *A. percula* were found to originate from the respective reef of settlement^5,6^. Remarkably, 32 % of *A. polymnus* larvae settled within two hectares of their birth site^7^. Coral reef fish larvae may use a wide range of cues to find the way back to their natal reef^8^. For example, celestial cues in combination with a time-compensated sun compass are used by the cardinal fish *O. doederleini* larvae^9^. Anemonefish may use similar mechanisms for orientation, which underlines the necessity for them to have a functional timing system. Not only dispersal but also settlement involves the function of an endogenous timekeeping system. Reef fish larvae enter the coral reef during dusk and night^10^ and settlement takes place during the night^11^. The available data indicate that it is vital for the fish to enter the reef at a particular time of day^12^.

The mechanisms whereby coral reef animals adapt to and anticipate daily changes in their environment remain poorly understood. The fascinating clown anemonefish *A. ocellaris* (family: Pomacentridae) represents an ideal model organism to study the contribution of the circadian clock to shaping coral reef fish biology. Adult anemonefish participate in a remarkably close, three-way mutualism with anemones and their zooxanthellae endosymbionts^13^. They are therefore restricted to the photic and mesophotic zones due to their obligate association with anemones. Host anemones, like *E. quadricolor* together with the inhabiting anemonefish predominantly occur in shallow water^14^. Furthermore, like most coral reef fish, clown anemonefish have a larval dispersal stage with larvae hatching at night^15^, leaving the natal reef immediately after hatching and staying for up to 12 days in the pelagic zone.

One major time-keeping mechanism is the circadian clock, which most organisms, including plants, fungi, bacteria and animals possess^16^. The circadian clock allows organisms to anticipate daily environmental changes and consequently, many physiological and behavioural processes are temporally organized and synchronized with the daily 24 hours cycle^17,18^. The use of fish as models for studying the circadian clock, most notably the zebrafish *Danio rerio*, has provided new insight into the function and evolution of the clock^19,20^. In contrast to mammals, almost all fish tissue clocks are directly light-entrainable. Exploiting this special feature, it has been possible to study clock regulation by light even in fish-derived cell cultures. The circadian clock in zebrafish like all other vertebrates, consists of transcriptional/translational feedback loops. The core circadian clock consists of positively and negatively acting elements: Heterodimers comprised of CLOCK and BMAL proteins which activate the transcription of *period* and *cryptochrome* genes. PERIOD and CRYPTOCHROME in turn suppress the transcriptional activation driven by the CLOCK and BMAL transcription factors, thereby reducing *period* and *cryptochrome* expression levels and so ultimately enabling a new cycle of activation driven by CLOCK and BMAL. This gene regulatory circuit thereby directs circadian rhythms of gene expression^21^.

While sunlight is one of the most important timing cues and fundamentally enables photosynthesis for coral reefs, it is also associated with exposure to damaging UV radiation. The ozone layer is at its thinnest near the equator and water turbidity is also low, leading to very high UV irradiance in coral reefs^22^. Both reef fish and anemones have developed multiple adaptation strategies to minimize UV induced damage. These include behavioural avoidance strategies such as occupying shady areas as well as the production of UV light absorbing protective substances^23,24^. Additionally, DNA damage induced by UV light is repaired by the efficient photoreactivation DNA repair system via light dependent catalysis by the photolyase enzymes, close relatives of the cryptochromes^25^.

Here we have investigated the diel rhythms of clown anemonefish at different stages of development to better understand the timing of dispersal and settlement in this species. We reveal a striking shift from nocturnal to diurnal locomotor activity during the development of larval clownfish into the juvenile forms. In turn, we document rhythmic circadian clock gene expression in this species and show that its phase is not altered during the shift in behaviour. We also demonstrate light-inducible *photolyase* gene expression in the clown anemonefish which is comparable between larval and juvenile fish and closely resembles that previously observed in zebrafish. In order to minimize the need to sacrifice extremely valuable adult clown anemonefish for studying temporal changes in gene expression at high resolution and under different lighting regimes, we have established an embryonic *A. ocellaris* cell line and used this to study circadian clock function. This confirmed that the properties of the clown anemonefish circadian clock closely resemble those of the zebrafish. Therefore, we reveal important changes in activity patterns during development, while rhythmic clock gene expression remains relatively unchanged.

## Results

### Activity patterns in larval, juvenile and adult clown anemonefish

**Figure 1:**
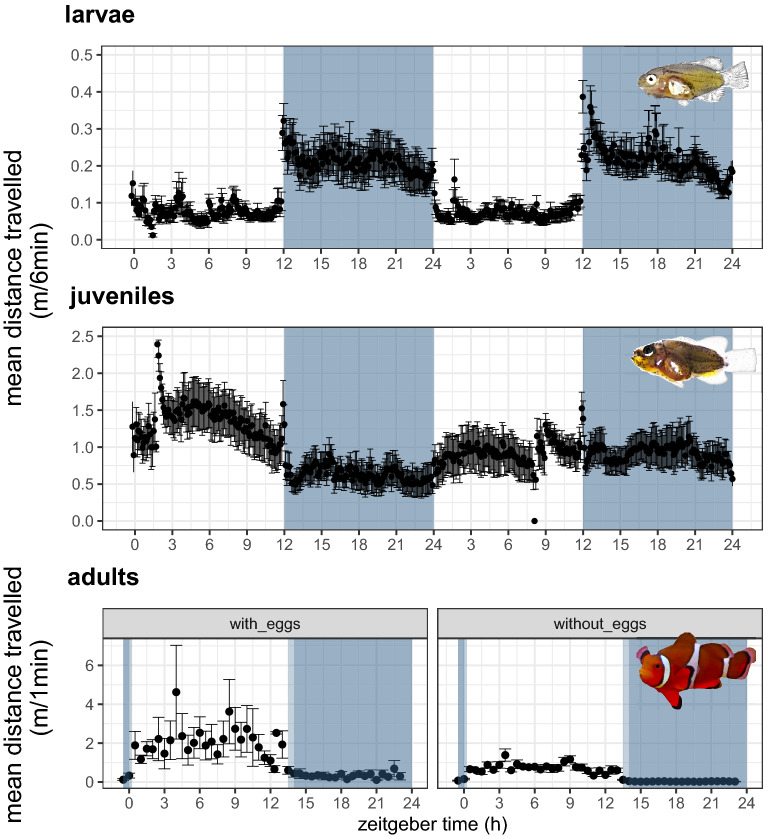
Representative locomotor activity determined by mean distance travelled ± *SEM* of *Amphiprion ocellaris* in 11–13 dph old larvae (n = 15–23), ca. 100 dph old juveniles (n = 9–18) and several years old adults (n = 4). Bluish-grey background refers to lights off. The mean locomotor activity of two consecutive days (larvae and juveniles) in m/6 min or two independent days (adults) in m/1 min are shown, dependent on zeitgeber time (h). Movement of larvae and juveniles was tracked continuously and the distance covered was summed up per 6 min. Adults were tracked manually half-hourly for one minute.

The habitat of very young larvae and adult animals is extremely different - young larvae are dispersing in the open ocean, while adults are highly sedentary in close mutualism with their host anemone. The locomotor activity of *A. ocellaris* larvae ranging from 7 to 23 days post hatching (dph), juveniles from 52 to 56 dph, 86 to 91 dph and 98 to 106 dph and breeding clownfish pairs (several years old) was observed during exposure to a Light:Dark (LD) cycle under laboratory conditions. We tested for rhythmic locomotor activity and also calculated the diurnality index (D)^26^ which represents a quantitative measure of the degree of nocturnal or diurnal activity, ranging from -1 (all animals are nocturnal) to +1 (all animals are diurnal). Larval and juvenile clownfish (> 96%) showed daily activity rhythms (*p* < 0*.*05, Fig. 1). All larvae were nocturnal (Fig. 1, D = -1). However, the nocturnality of animals diminished with increasing age (juveniles 52–91 dph: -0.1 < D < -0.5). Older juveniles (98–106 dph) were mostly diurnal (D =  +0.38) and all adult clownfish were exclusively diurnal (D =  +1), irrespective of breeding status. Importantly, locomotor activity levels tended to increase just before the onset or offset of the light period showing one of the most characteristic features of circadian clock regulated behaviour, the anticipation of the daily light–dark cycle (Fig. 1).

### Gene expression of clock and photoreactivation DNA repair genes in larval and juvenile clownfish

**Figure 2:**
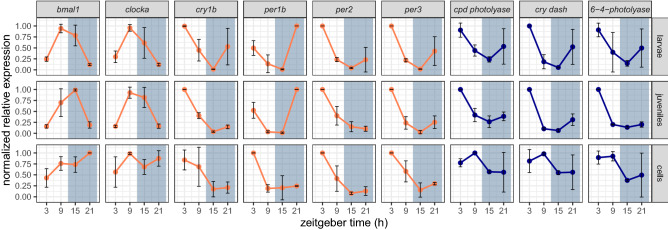
Mean normalized expression of six clock genes (orange traces): *bmal1*, *clocka*, *cry1b*, *per1b*, *per2*, *per3* and three genes of the photoreactivation DNA repair mechanism (blue traces): *cpd photolyase*, *cry dash*, *6-4-photolyase* of *Amphiprion ocellaris* cells, larvae and juveniles. Expression was quantified using quantitative real time PCR at zeitgeber time (h) 3, 9, 15 and 21. Lights were switched off at zeitgeber time 12, indicated by bluish-grey background. Per timepoint, three samples for larvae and juveniles and two samples for cells were analysed. The cycling expression pattern of clock genes as well as genes of the photoreactivation repair mechanism was similar between larvae and juveniles. The expression pattern of the period genes and *cry1b*, as well as the *photolyase* genes in the EAO cell line resembled those in the larvae and juveniles. However, expression of *clocka* and *bmal1* showed a non-cycling pattern in the EAO cells compared with the high amplitude rhythmic expression observed in the animal samples.

Does the shift from nocturnal to diurnal activity in *A. ocellaris* reflect a corresponding shift in the function of the core circadian clock mechanism? To address this question we compared the mRNA expression profile of a set of six core clock genes in whole body RNA extracts prepared from larval and juvenile clownfish exposed to a 12:12 h LD cycle. Specifically, we examined the temporal expression profile of six core clock genes (*bmal1*, *clocka*, *cry1b*, *per1b*, *per2*, *per3* sequences derived from an existing transcriptome database^27^) with a relatively low resolution of four timepoints over the course of one day-night cycle. The mean normalized gene expression is shown in Fig. 2. The peak of RNA expression of *per2*, *per3*, *cry1b* was at zeitgeber time (ZT) 3 and of *per1b* at ZT21 (*p *< 0*.*05), while genes encoding the positive elements of the core clock regulatory loop, *bmal1* and *clocka* were instead expressed highest at the day-night transition in both larvae and juveniles (a peak at ZT9 for *clocka* in larvae and juveniles and for *bmal1* at ZT9 in larvae and at ZT15 in juveniles (*p *< 0*.*05)). Thus, the rhythmic expression pattern for each gene was equivalent in nocturnal larvae and diurnal juveniles.

Given the importance of an efficient DNA repair system in the coral reef environment, we also investigated the expression of three genes of the photoreactivation DNA repair mechanism (*cpd photolyase*, *cry dash* and *6-4-photolyase*) (Fig. 2). They were most highly expressed during the day with a peak at ZT3 (*p *< 0*.*05) in larvae and juveniles, consistent with the predominantly light driven expression observed in other fish species, such as zebrafish and goldfish.

### Clock and light regulated transcriptional rhythms in clownfish

In order to explore in more detail the molecular mechanisms directing rhythmic gene expression in *A. ocellaris*, our next goal was to study in depth the regulation of clock gene expression under a range of lighting conditions. Such an experiment would be entirely impractical using fish, particularly at high resolution, due to the large numbers of animals required which would exceed the capacity of our animal facility. Furthermore, such a costly experiment would be objectionable purely based on ethical considerations. Therefore, we chose to establish an *A. ocellaris* cell line from enzymatically dissociated embryos, the embryonic *A. ocellaris* (EAO) cell line. Based on our previous studies of zebrafish and cavefish cell lines^28,29^, such a clownfish cell line should possess a directly light-entrainable circadian clock mechanism, comparable to that observed *in vivo*. To test this prediction, we compared rhythmic gene expression in the EAO cell line with that of larvae and juveniles as described above (see Fig. 2). In the EAO cell line, RAIN analysis revealed two rhythmic genes, *per2* and *per3* with a peak at ZT3 (*p *< 0*.*05), similar to larval and juvenile clownfish. Expression of *cry1b* seemed to be similar, compared to that of the fish with the highest expression at ZT3, a decrease until ZT15 and a small increase until ZT21. *Per1b* was most highly expressed during the day, but not at ZT21, as seen in clownfish larvae and juveniles. Thus, the rhythmic expression pattern for the *cryptochrome* and *period* genes was equivalent in the EAO cells, larvae and juveniles. Furthermore, consistent with our results in larvae and juveniles, in EAO cells, the *photolyase* genes were expressed at higher levels during the day than during the night, but without the clear peak at ZT3. However, interestingly, rhythmic mRNA expression of *bmal1* and *clocka* in larvae and juveniles did not resemble the non-cycling expression pattern in the clownfish cell line.

**Figure 3:**
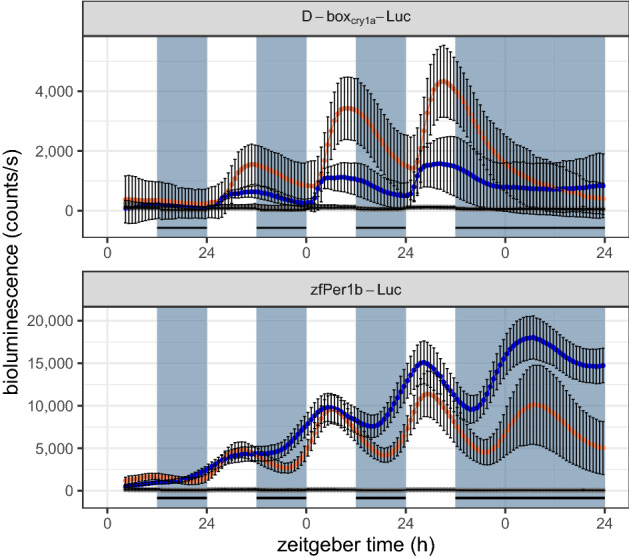
Luciferase assay showing rhythmic bioluminescence of *Amphiprion ocellaris* EAO (orange) and zebrafish PAC-2 cells (blue) transfected with D-box_*Cry1a*_-Luc (above) or zf*Per1b*-Luc (below) constructs. A negative control of non-transfected cells is shown in grey. For each condition, the mean of eight independently transfected wells and standard deviation is shown. Cells were exposed to a 12:12 h LD cycle, indicated by bluish-grey background and black stripes. Both cell lines, transfected with zf*Per1b*-Luc showed a comparable robust daily rhythm, even under DD. Cells transfected with D-box_*Cry1a*_-Luc were only rhythmic during the LD cycle.

We next wished to study at higher temporal resolution, clock and light-regulated transcription in *A. ocellaris* cells. With this aim we examined the expression of transiently transfected luciferase reporter gene constructs based on zebrafish *cryptochrome* and *period* gene promotors which we have previously characterized extensively in zebrafish cell lines. Specifically, zf*Per1b*-Luc has been previously reported to contain multiple E-box enhancer elements and so is strongly regulated by the core circadian clock elements CLOCK and BMAL^21^. Instead, D-box_*Cry1a*_-Luc contains multimerized copies of the D-box enhancer element which we have revealed serves as a light responsive enhancer element in most fish species analysed so far^30,31^. In particular, this enhancer directs light induced expression of *photolyase* genes^32^. EAO cells transfected with both constructs were exposed for four days to a 12:12 h LD cycle followed by one day in constant darkness (DD) and during the entire period, levels of bioluminescence generated by the reporters was measured at 48 min resolution by an *in vivo* bioluminescence assay (Fig. 3). With the D-box_*Cry1a*_-Luc construct, both transfected EAO and control zebrafish PAC-2 cells showed a robust bioluminescence rhythm in LD cycle, which was immediately absent in DD (Fig. 3, above), in agreement with zebrafish and *A. ocellaris* sharing light-regulated D-box function. This is also consistent with the light inducible expression observed for the *A. ocellaris photolyase* genes (Fig. 2). On the contrary, EAO and PAC-2 cells, transfected with the zf*Per1b*-Luc construct, showed a robust cycle of bioluminescence in LD, which persisted even in DD with a peak in the subjective day (Fig. 3, below), consistent with the presence of a robustly light-entrainable clock in both EAO and PAC-2 cell lines.

## Discussion

In our study we have revealed a considerable change in locomotor activity during developmental maturation of *A. ocellaris* from nocturnal larvae to diurnal adults. In juveniles we found both patterns of activity with an increase of diurnal activity with increasing age. Interestingly, the phase of rhythmic expression of the core clock genes did not change during ontogeny.

We provide new insight about locomotor activity of *A. ocellaris* larvae over a range from 7 to 23 dph. Larvae and juveniles up to 90 dph were mostly nocturnal (D < -0.1) and did not change their behaviour during the natural settlement period at the reef (around 12 dph). In the wild, undisturbed locomotor activity has not been observed in dispersing coral reef fish larvae. There is only indirect evidence for nocturnal locomotor activity e.g. that settlement occurs at dusk and night^10^. Thus, during the night, but not during the day, pomacentrid larvae prefer reef sound which might guide them to a reef for settlement^33^. Also, under laboratory conditions, larvae of the closely related species *A. melanopus* and *A. percula* moved at higher speeds during the night compared to the day when they were 7 and 9 dph old^34^. Nocturnal settlement of another pomacentrid species *Dascyllus trimaculatus* was observed in the field^11^, with these fish switching from nocturnal to diurnal behaviour directly after settlement. Larvae post metamorphosis at an age of 7–14 dph were protected by anemones, so they were able to settle at this age^35^. Anemonefish have been assumed to settle after 6–10 days in order to seek out an anemone^15^.

Interestingly, during our experiments, the switch from nocturnal to diurnal behaviour did not occur during the natural settlement time when clownfish arrive at a reef, but instead much later. In our experimental setup we could not provide anemones, so the absence of a potential host anemone for settling might have delayed the behavioural switch in the larvae. In the wild, clownfish are always found in close proximity to an anemone; indeed, not being able to find a suitable anemone is lethal^36^. It is possible that clownfish which initially cannot find an anemone, hide during the day to avoid predation and delay their settlement in an anemone. Future studies are required to explore whether clownfish larvae might exhibit wide temporal plasticity in this regard. However, we should be cautious not to overinterpret the exact timepoint when clownfish switch from nocturnal to diurnal behaviour since the laboratory conditions might have influenced this process. Nevertheless, it is important to emphasize that the switch from nocturnal to diurnal behaviour was not evoked by feeding or handling. All animals, from larvae and juveniles to adult clownfish were fed during the day, therefore it is likely that this change of activity is innate.

We analysed the rhythmic gene expression of *A. ocellaris* circadian clocks. Rhythmic clock gene expression overall is very similar to that described for zebrafish, the most studied fish model regarding circadian clock gene expression^37,38^ as well as other fishes including the migrating *Oncorhynchus mykiss*^39^ and the marine *Sparus aurata*^40^. The *bmal1* and *clock* genes serve as positive elements within the core clock mechanism, activating the transcription of the *cryptochrome* and *period* clock genes. The expression of *A. ocellaris clocka* and *bmal1* is comparable with the expression of zebrafish *clock*^28,37^ and *bmal*^37^ and *O. mykiss clock* and *bmal1*^39^ with a peak at around ZT12. Heterodimers of CLOCK and BMAL activate the transcription of *period* and *cryptochrome* genes including *per1b* via binding to E-box promotor elements^29^. Expression of *per1b* was highest at late night in *A. ocellaris*, similar to *per1* expression in the retina of the nocturnal *Solea senegalensis*^41^, the reef fish *Siganus guttatus*^42^ and zebrafish larvae^37^. In zebrafish the expression of period gene *per2* is light driven and therefore important for entrainment of the clock by light^30^. In the clown anemonefish, *per2* is expressed at highest levels during the day, similar to what is found in zebrafish^28^ and *S. senegalensis*^41,43^. We also found highest expression of *cry1b* at ZT3 and also elevated expression at ZT9, which is comparable with the peak of expression of *cry1b* in zebrafish during the middle of the light phase^21^.

All of our behavioural analysis together with the assays for clock gene expression in clownfish larvae and juveniles were performed under LD cycle conditions. Therefore, it is theoretically conceivable that the observed rhythms are driven acutely by the lighting conditions rather than being driven by a self-sustaining circadian clock. Definitive proof of circadian clock regulation *in vivo* is the persistence of behavioural or clock gene rhythmicity even under prolonged periods of DD. However, due to the high cost in terms of number of animals required as well as the small size and sensitivity of clownfish larvae combined with the need for daily feeding and water changing, we could not analyse behaviour or clock gene expression during DD in whole clownfish. Instead, we used a newly established clownfish cell line that gave us the possibility to analyse the circadian clock over several days with high temporal resolution. The zebrafish zf*Per1b*-Luc reporter is clock driven^29^ and EAO cells showed a robust bioluminescence rhythm with this construct which persisted in DD. This provides important evidence for clown anemonefish possessing an endogenous and cell autonomous, directly light-entrainable clock. We also tested expression of a D-box_*Cry1a*_-Luc reporter to analyse whether light drives rhythmic gene expression in these clownfish cells. The bioluminescence was robustly rhythmic in LD cycle conditions, with reporter expression increasing exclusively during the light period, before descending to lower basal levels following return to darkness. This rhythmicity was immediately abolished under DD, which provides additional evidence the zebrafish and clown anemonefish circadian clock share similar molecular mechanisms whereby light regulates expression and clock function. Furthermore, consistent with this conclusion, in our behavioural analysis we observed a characteristic upward tendency in locomotor activity occurring just before the onset or offset of the light period. This anticipatory activity is entirely consistent with regulation by a functional clock mechanism that would provide the necessary basis for timing such as that observed time-compensated sun compass orientation, which was found in coral reef fish *O. doederleini*^9^.

Interestingly, while robust rhythmic mRNA expression of the *clocka* and *bmal1* genes was observed in clownfish juveniles and larvae, these genes appeared to be arrhythmically expressed in EAO cells. Differences in the amplitude of rhythmic *clock* gene expression have been frequently reported between species and may reflect differences in the relative contributions of transcriptional and post-transcriptional mechanisms to driving circadian rhythms in *clock*- and *bmal*-mediated transcriptional activation^44^. Furthermore, within a single species, tissue and cell type-specific differences in the rhythmic expression pattern for individual clock genes have been frequently observed^45^. For example, rhythmic clock gene expression is absent in mouse embryonic stem cells^46^. Given that the EAO cell line was generated from a complex pool of cells obtained from enzymatically dissociated entire *A. ocellaris* embryos, it is problematic to determine precisely which original cell type represented the source of this cell line. Furthermore, cell phenotypes may be influenced by the inevitable selection process involved in repeated propagation of the cells under artificial cell culture conditions. Nevertheless, based on our previous comparison of the zebrafish circadian clock mechanism *in vivo* with that in zebrafish embryo-derived cell lines^29^ the observed differences in clock gene expression between EAO cells and *A. ocellaris* larvae and juveniles are unlikely to indicate radical differences in the clock mechanism.

Interestingly, although the locomotor activity pattern changed dramatically during maturation, the clock gene expression pattern remains similar in larvae and juvenile clown anemonefish. There are theoretically a number of possible interpretations for this observation. Inaccuracies in our analysis seem unlikely given that our experimental approaches for behavioural and gene expression analysis follow well established protocols that have been applied to many other fish species, including the well-studied zebrafish^37^. Furthermore, given that the kinetics of clock gene expression has been firmly linked with the timing of clock regulatory targets^38^ it also seems unlikely that the phase of rhythmic clock gene expression observed *in vivo* in clownfish larvae and juveniles has no functional consequence. However, given that our analysis of gene expression patterns was performed using whole fish extracts and thus represented a mixture of different peripheral tissues, it is possible that changes in the phase of rhythmic clock gene expression did occur in small collections of cells in the central nervous system, that might be important for directing these changes in locomotor activity. In this regard, our results are consistent with other studies comparing clock gene expression in diurnal and nocturnal species. No general shift in clock gene expression was found between diurnal and nocturnal mammals (reviewed in^47^). Furthermore, in the case of fish, depending on the precise timing of regular feeding time, the gilthead sea bream (*S. aurata*) can be nocturnal or diurnal, but importantly, clock gene expression within the brain remains identical^40^. Similar patterns were also found in *Dicentrarchus labrax*^48^. Thus, our findings provide additional evidence that the switch between nocturnal and diurnal behaviour might be controlled downstream of the core clock or alternatively might result from switches in the phase of the core clock rhythmicity occurring in a subregion of the brain.

The light-driven, rhythmic expression of genes involved in repair of UV-damaged DNA also show a very similar expression pattern over ontogeny. While UV irradiance is very high in coral reefs causing damage (e.g. decreased growth rate, DNA damage and oxidative stress)^22^, UV light might be used e.g. by reef fish larvae for navigation during foraging^49^. Adult clownfish are closely linked to anemones, which are located in the photic zone of coral reefs. We found higher expression of genes of the photoreactivation DNA repair mechanism during the day, compared to night in both, larval and juvenile clown anemonefish. This expression pattern was similar in our embryonic, clownfish derived cell line. These findings provide basic evidence that these fish might rely on photoreactivation DNA repair as a major strategy to survive DNA damage, beside other methods such as the production of UV-absorbing substances^22,23^. During the night, anemones close with clownfish hiding inside. Anemones and their photosynthetic active zooxanthellae are dependent on sunlight and open up during the day and thereby facilitate diurnal activity in adult clownfish. The mutualism is highly advantageous for all three partners, fish, anemone and zooxanthellae. On the one hand, anemones provide a safe habitat for the fish where they can live and breed^36^, which might facilitate remarkable longevity of anemonefish in comparison to other pomacentrids^50^. On the other hand, anemones are protected from predators by anemonefishes^51^ and they perform worse if they do not have a symbiotic partner^52^. Part of the three-way mutualism is a nitrogen and carbon flux circulating between fish, anemone and its embedded zooxanthellae^53,54^. Our study provides a first basis to study a fascinating, complex and multi-layered interaction between clocks from organisms which evolved together over 10 million years^55^.

## Methods

All animal procedures were approved by the Animal Care and Use Committees of the Niedersächsisches Landesamt für Verbraucherschutz und Lebensmittelsicherheit (LAVES, Oldenburg, Germany), Az.: 33.19-42502-04-20/3338 and performed in accordance with the relevant guidelines and regulations and are reported in compliance with the ARRIVE guidelines.

### Husbandry of clown anemonefish

Adult clownfish were kept for breeding and observation in a circulating 800 l water system. Each pair was separated in 40–60 l aquaria with at least one anemone *E. quadricolor*, one living rock and one terracotta pot. The salinity of water was approximately 33, the water temperature was 28 °C. Aquaria were illuminated with a 13:11 h LD cycle by a Fluval Marine & Reef 2.0 LED light system (HAGEN Deutschland, Holm, Germany) with 33.3 W/m^2^. Light intensity was measured using X1-3 optometer with XD-45-HB sensor (Gigahertz Optik, Türkenfeld, Germany) directly under the light source. Clownfish were fed with frozen food once per day.

Eggs from two breeding pairs, attached to a terracotta pot, were transferred before hatching into a 25 l tank at 28 °C where eggs were aerated. Tanks were illuminated using a white LED system (185 mW/m^2^) with a LD cycle of 12:12 h. Larvae hatched within two days. Thereafter, the terracotta pot was removed. Larvae were fed with *Brachionus* sp. until 6 dph and artemia nauplii (*Artemia* sp. (Sanders, South Ogden, USA)), starting from 3 dph, enriched with *Nannochloropsis salina* and Easy DHA Selco (Inve, Salt Lake City, USA). Larvae were used for behavioural tests and gene expression analysis.

### Establishment and cultivation of the embryonic *A. ocellaris* cell line

We established an embryonic *A. ocellaris* (EAO) cell line to analyse clock gene expression in living cells and facilitate further analysis, so not being dependent on sacrificing valuable adults. The EAO cell line was prepared using a method previously applied with zebrafish^56^. Eggs were scraped from one terracotta pot three days post fertilization and disrupted mechanically followed by 15 min incubation with Trypsin/EDTA (Gibco by Thermo Fisher Scientific, Paisley, United Kingdom). The cell suspension was transferred to a 6-well plate (Cellstar by Greiner Bio-One, Frickenhausen, Germany) and cultivated in Leibovitz’s L-15 medium supplemented with 15% fetal bovine serum (FBS), 100 U/ml penicillin/100 µg/ml streptomycin and 25 µg/ml gentamicin (all Gibco) at 27 °C. After one week, cell debris was removed and cells were washed with 1× PBS (Gibco) and were further incubated in culture medium. For passaging, a confluent cell monolayer was washed with 1× PBS, detached from the culture substrate by incubation with Trypsin/EDTA at 37 °C and then reseeded in culture medium in 25 cm^2^ flasks (Cellstar by Greiner Bio-One).

### Behavioural experiments

To analyse potential differences in activity patterns during development, we studied the diel activity of *A. ocellaris* larvae (7–23 dph), juveniles (ca. 52–106 dph), and breeding pairs (several years old). We observed activity of 45 larvae and 36 juveniles and 4 adults. Animals which were observed for less than 24 h were not further analysed (three larvae). For recording behaviour, all clownfish tanks were illuminated with infrared light at 940 nm wavelength, which is not seen by fish^57^ and activity was recorded using webcams (Logitech c920) adapted by removing the infrared cut-off filter.

#### Activity recordings of clownfish larvae and juveniles

Clownfish larvae were observed individually in 6-well plates with 15 ml water volume and within 30 ml tanks with squared base area, where larvae behaved similarly. Juvenile clownfish were observed in 150 ml tanks with a square base area. Water temperature was maintained at 28 °C. Visual light was supplied by CCFL backlight from a striped TFT monitor (787 mW/m^2^) with a 12:12 h LD cycle. Every day at a different time, one third of the water was exchanged and the animals were fed with live artemia.

The activity of larvae and juveniles was recorded with Multiviewer (resolution: 640:480 pixel, one frame per second) (Computer System Department, University of Murcia) and compressed using VirtualDub 1.10.4 and xvid codec. The movement of larvae was tracked with FishTracker (Computer System Department, University of Murcia). To verify reliable tracking, data was also analysed with EthoVision XT (Noldus Information Technology).

#### Activity recordings of adult clownfish

Activity of adult clownfish was recorded with two cameras using Yawcam 0.6.2 (one picture per second with a resolution of 1920:1080 pixel), one camera from above and one at the front. The position of fish was tracked manually per second every 30 min for 1 min with ImageJ^58^ to calculate their three-dimensional movement. *A. ocellaris* undertake intensive egg care, therefore breeding pairs were observed with as well as without a clutch off eggs in their keeping aquarium to avoid handling stress. During video recording, the room was entered only once for daily fish care. Fish were not fed during recording.

#### Data analysis

Data analysis was performed with R 3.6.1^59^. The distance larval and juvenile clownfish moved per second was calculated from x- and y-coordinates, obtained from FishTracker using Pythagorean theorem. The distance covered was summed up individually for each fish per 6 min and translated from pixel to centimetres. The mean distance covered was calculated for animals grouped according to age for graphical representation with ggplot2^60^. For adult clownfish the three-dimensional movement was calculated based on x-, y- and z-coordinates within Excel (Microsoft) and the movement per second was summed up per analysed one minute.

Data was statistically analysed with RAIN (Rhythmicity Analysis Incorporating Nonparametric methods)^61^ to identify significant rhythmicity. Therefore, distance moved was summed up per hour. Animals were counted as diurnal for ZT between 0 and 11 and as nocturnal for ZT between 12 and 23 h. The diurnality was calculated as an index, based on^26^. This is an index from +1 (all animals are diurnal) to -1 (all animals are nocturnal) and was calculated with this formula: ${\text{D}} = \frac{{N_{diurnal} {-} N_{nocturnal} }}{{N_{diurnal} + N_{nocturnal} }}$.

### Analysis of core clock and photoreactivation DNA repair genes

To analyse the core clock mechanism of *A. ocellaris*, gene expression of six genes was analysed by qRTPCR: *bmal1*, *clocka*, *cry1b*, *per1b*, *per2*, *per3*. Additionally, we analysed the expression of three genes of the photoreactivation DNA repair mechanism: *cpd photolyase*, *cry dash*, *6-4-photolyase* (also known as *cry5*). Primers were designed using Primer3^62–64^ for an annealing temperature of 60 °C and checked for specificity with Primer-BLAST^65^. Sequences for all genes were obtained from a genome hybrid assembly of an *A. ocellaris* genome using Oxford Nanopore and Illumina^27^. See table 1 for primer sequences and accession numbers.

**Table 1:** Primers used for quantitative real time PCR to analyse gene expression.

Primer	Sequence (5′–3′)	Accession number
ef1a fwd	AAGAAGCTTGAGGATGGGCC	ENSAOCT00000026175
ef1a rev	ACAGTCTGCCTCATGTCACG	
rpl13a fwd	GCAGGCAGAAAAGAACGTCG	ENSAOCT00000016640
rpl13a rev	GCCAGTGGTAGCTAGCTCAG	
cry1b fwd	TGCATCTACATCCTGGACCCT	ENSAOCT00000032644
cry1b rev	GACAATGCAGCAGAAACCTCC	
clocka fwd	TATCTGACCCGGCCTCATCA	ENSAOCT00000012399
clocka rev	GTGACGTCATCTCCACAGCA	
bmal1 fwd	TGAAATGCAACCGACCCTCA	ENSAOCT00000006452
bmal1 rev	AGCTCTTACGGTCCGCTTTT	
per1b fwd	AGTCTCCCCTCTTCCAGTCC	ENSAOCT00000018592
per1b rev	ATTGGCTGAGCTCTGTCCAC	
per2 fwd	GCAGCAAAGCCAGACCTAGA	ENSAOCT00000004079
per2 rev	CACCACCGCTAGACTCTTGT	
per3 fwd	TCATCAACCCTTGGAGTCGC	ENSAOCT00000015706
per3 rev	TTGAACGAGCAGCAAACACG	
6–4-photolyase fwd	TCATCAACCCTTGGAGTCGC	ENSAOCT00000010406
6-4-photolyase rev	TTGAACGAGCAGCAAACACG	
cry dash fwd	TCATCAACCCTTGGAGTCGC	ENSAOCT00000006663
cry dash rev	TTGAACGAGCAGCAAACACG	
cpd photolyase fwd	CCTCCCTCTGCATGTGTGTT	ENSAOCT00000031071
cpd photolyase rev	CACCGTGAAGCAAGTGGAAC	

Primers were designed using Primer3 based on sequences obtained from ensembl.org (Accession Number) originating from^27^. *Ef1a* and *rpl13a* are used as housekeeping genes, *cry1b*, *clocka*, *bmal1*, *per1b*, *per2* and *per3* are elements of the core clock mechanism. *6-4-photolyase*, *cry dash* and *cpd photolyase* constitute the photoreactivation DNA repair mechanism.

#### Gene expression analysis using qRTPCR

Larval (10 dph) and juvenile clownfish (105 dph) were sampled at 3, 9, 15 and 21 h after light was switched on from two separate tanks. Before sampling, animals were left undisturbed for 12 h. Larval and juvenile clownfish were euthanized by an overdose of MS-222 (Pharmaq, Hampshire, UK) in 4 °C cold salt water according to the ethical protocol standards. Samples were stored in RNA-stabilising buffer^66^ at −80 °C until RNA extraction.

To analyse gene expression of the EAO cell line, cells were seeded in 25 cm^2^ flasks and incubated for one week under a 12:12 h LD cycle (452 mW/m^2^). Confluent cell monolayers were harvested, by scraping in culture medium using a cell scraper. The cell suspension was then centrifuged at 1500 g for 5 min at 4 °C. The pellet was washed twice with 1× PBS and stored at −80 °C until RNA extraction.

Total RNA was extracted using Monarch Total RNA Miniprep Kit (New England Biolabs, Frankfurt am Main, Germany) following the manufacturer’s instructions for cultured mammalian cells and tissue including DNase treatment. Deviating from that protocol, whole animals were homogenized in 1× DNA/RNA Protection Reagent using a bead beater (MagNa Lyser, Roche Diagnostics, Mannheim, Germany) with glass beads (diameter 1.25–1.65 mm, Carl Roth, Karlsruhe, Germany) at 5000 rpm for up to 6 min.

The amount of RNA and its purity was determined using an optical spectrometer (BioSpectrometer basic, Eppendorf, Hamburg, Germany) at 230, 260 and 280 nm. Samples were treated using TURBO DNA-free Kit (Invitrogen by Thermo Fisher Scientific Baltics, Vilnius, Lithuania) following the manufacturer’s instructions.

Extracted RNA was reverse transcribed to cDNA using iScript cDNA Synthesis Kit (Bio-Rad Laboratories, Feldkirchen, Germany). Quantitative PCR was conducted with GoTaq qPCR Master Mix (Promega, Madison, USA) in a 10 µl reaction volume on a LightCycler 480 Instrument II (Roche). Three technical replicates per gene and timepoint were performed. Efficiency was calculated using a dilution series ensuring efficiencies between 1.86 and 2.09. Data were analysed with LightCycler 480 Software, Version 1.5 (Roche). Cycle of quantification (C_*q*_) was calculated using the 2nd derivative maximum method.

Relative gene expression was calculated using expression of *rpl13a* and *ef1a* as housekeeping genes. Each experimental group, consisting of one animal per timepoint, which were handled together during the whole experimental procedure, was normalized separately using Excel (Microsoft). The mean was calculated with the normalized data for larvae, juveniles and cells. Rhythmicity was analysed using RAIN^61^.

#### In vivo* luciferase assay*

EAO cells were transiently transfected using FuGene HD (Promega) transfection reagent according to the manufacturer’s instructions. One day before transfection, 2 × 10^4^ cells per well were seeded in a white 96-well plate (Nunc by Thermo Fisher Scientific, Roskilde, Denmark) and incubated at 27 °C for 24 h. Each well was transfected independently with a 4:1 ratio of reagent:DNA with 100 ng plasmid DNA. The cells were transfected with the reporter construct zf*Per1b*-Luc^29^ or D-box_*Cry1a*_-Luc based on^31^. As a control, zebrafish PAC-2 cells^30^ were transiently transfected in the same 96-well plate. Non-transfected cells were used as negative control for each cell line. After incubation for 24 h, the transfection solution was replaced by culture medium supplemented with 2 mM D-Luciferin (Biosynth, Bratislava, Slovakia). Cells were exposed to four 12:12 h LD cycles followed by 24 h DD. The bioluminescence was measured with a Topcount NXT counter (PerkinElmer, Shelton, USA) as previously described^29^. We imported the data into Excel (Microsoft) by using the "Import and Analysis" macro (S. Kay, Scripps Research Institute). Mean and standard deviation of eight independently transfected wells were calculated with R 3.6.1^59^ and shown using ggplot2^60^.

## Supplementary Information

Supplementary Information

## Data Availability

All data generated and analysed during the current study, including the used R-script, are included in this published article and its Supplementary Information files. Raw video files are available on request.
