# Supplementary material for: Finding Nemo’s clock reveals switch from nocturnal to diurnal activity
Source: Sci Rep. 2021 Mar 24;11:6801. doi: 10.1038/s41598-021-86244-9 (PMC7990958; doi:10.1038/s41598-021-86244-9)
Supplement: Supplementary file 1 — Supplementary Information [file 41598_2021_86244_MOESM1_ESM.pdf]

Finding Nemo’s clock reveals switch from nocturnal to diurnal activity

Gregor Schalm, Kristina Bruns, Nina Drachenberg, Nathalie Geyer, Nicholas S. Foulkes, Cristiano Bertolucci and Gabriele Gerlach

Table of Contents

1 Preface ... 2
2 Supplementary figure 1 ... 3
3 Supplementary table 1 ... 11
4 Load necessary packages ... 11
5 Gene expression of clock and photoreactivation DNA repair genes in larval and juvenile clownfish (figure 3) ... 12
5.1 Load data and adjust the data frame ... 12
5.2 Draw diagram ... 13
5.3 Statistics (RAIN) ... 14
6 Analysis of Behavioural Data ... 16
6.1 Calculate distance moved from position data ... 16
6.1.1 Larvae and Juveniles ... 16
6.1.1.1 Larvae 1 ... 17
6.1.1.2 Larvae 2 ... 18
6.1.1.3 Larvae 3 ... 20
6.1.1.4 Larvae 4 ... 22
6.1.1.5 Larvae 5 ... 23
6.1.1.6 Larvae 7 ... 26
6.1.1.7 Juvenile 1 ... 27
6.1.1.8 Juvenile 2 ... 29
6.1.1.9 Juvenile 3 ... 31
6.1.1.10 Juvenile 4 ... 32
6.1.2 Adults ... 34
6.2 Statistical analysis of behavioural data using RAIN ... 34
6.2.1 Data preparation ... 34
6.2.2 Statistcs ... 36
6.2.2.1 Calculation of diurnality index ... 50

6.2.3 Graphics 51
6.2.3.1 Figure 1 51
6.2.3.2 Additional: Seperate graph per animal 53
7 Luciferase assay 62

1 Preface

The included R-scripts are fully executable and were used to produce the figures and to calculate statistics included in the manuscript entitled "Clownfish circadian clocks: Switch from nocturnal to diurnal activity during development." by Gregor Schalm, Kristina Bruns, Nina Drachenberg, Nathalie Geyer, Nicholas S. Foulkes, Cristiano Bertolucci and Gabriele Gerlach. Following data is provided in the respective folders and can be downloaded here: https://cloudsync.uol.de/s/wzLRqarQt8LcF9b.

Folder	Data
./position/	Position data of behavioural observations
./position/time*.txt	files necessary for analysis of experiments "juvenile1" and "larvae5"
./Relquant.csv	Results of qRTPCR analysis
./adult_movement_data.csv	Movement data of adult clown anemonefish
./luciferase.txt	Data of luciferase assay
./results/	The results and analysed data derived using this script

Attention: A full run of the script takes about 1-2 days, especially because of the RAIN analysis of the behavioural data. Several files are stored during the run.

2 Supplementary figure 1

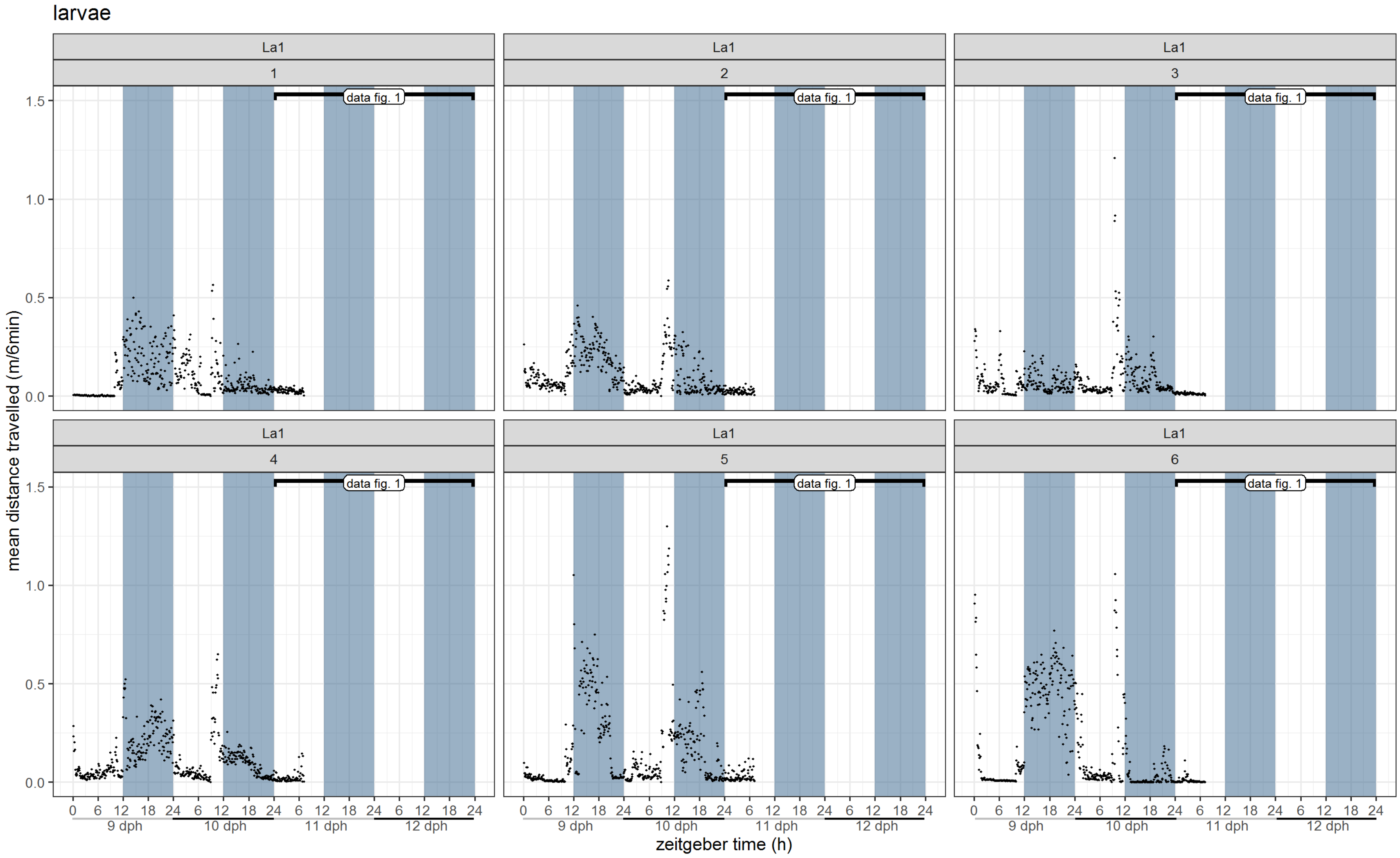

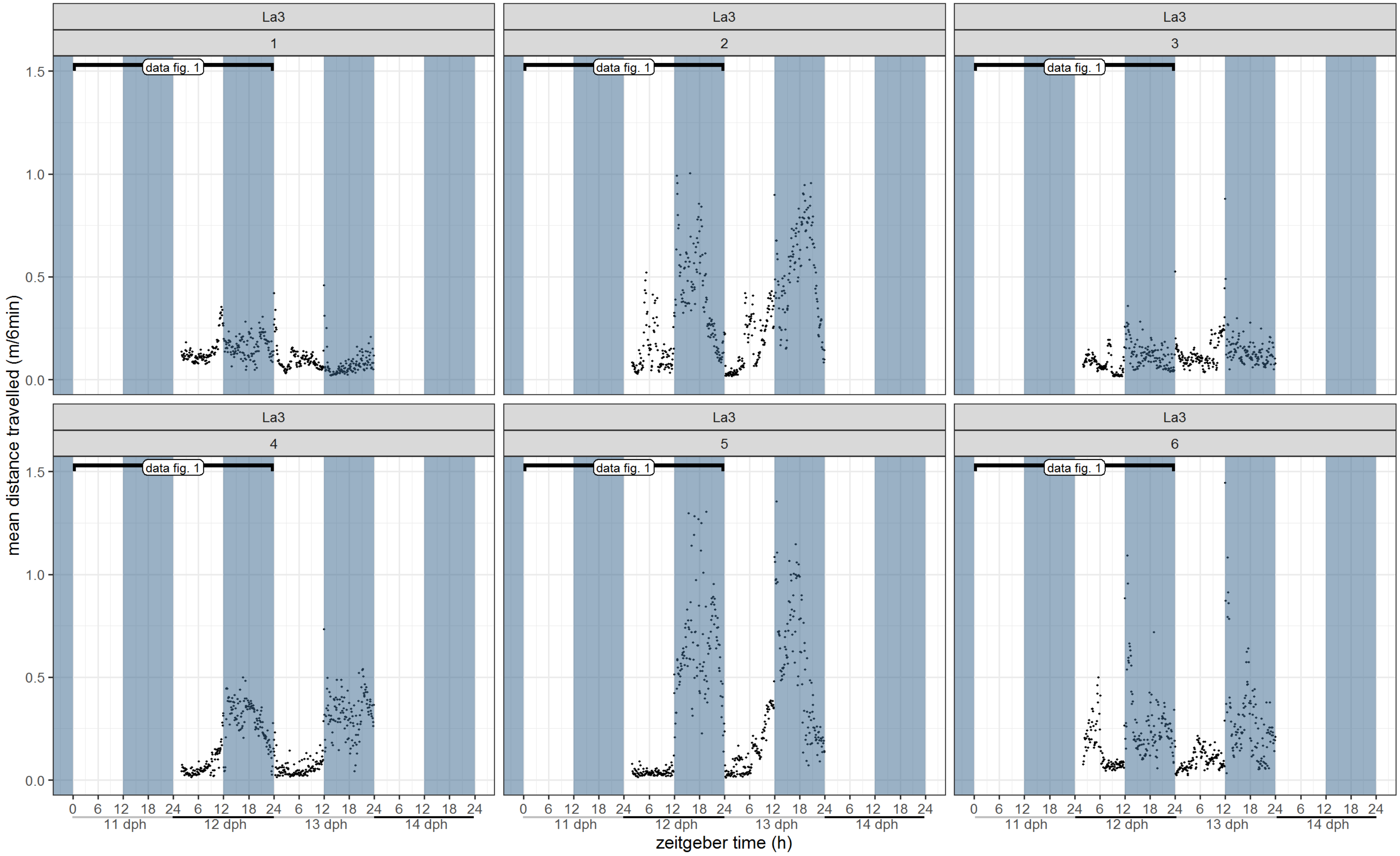
La3
1
2
3
4
5
6
data fig. 1
mean distance travelled (m/6min)
0.0
0.5
1.0
1.5
0
6
12
18
24
11 dph
12 dph
13 dph
14 dph
zeitgeber time (h)

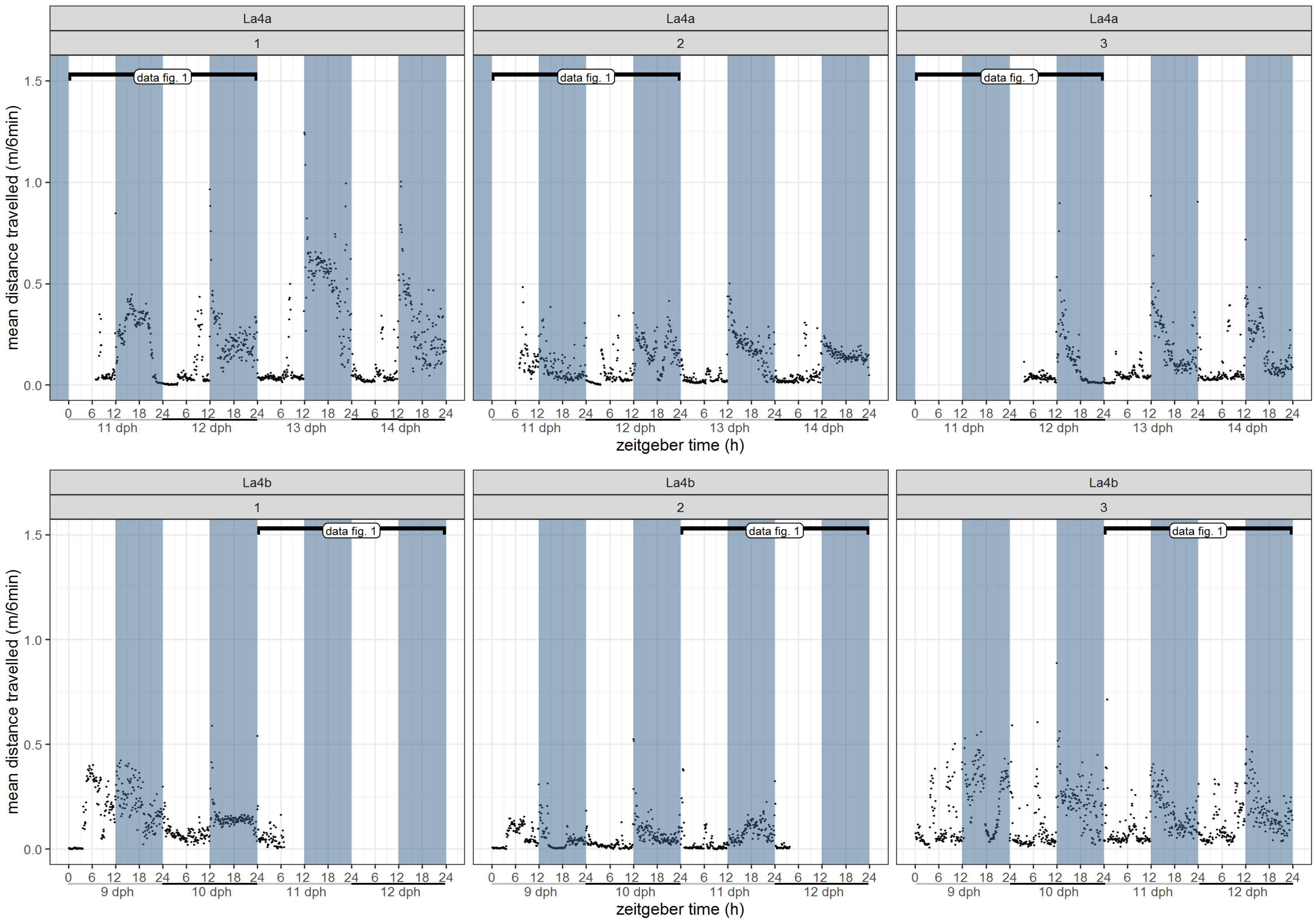

La4a
1
2
3
La4b
data fig. 1
mean distance travelled (m/6min)
0.0
0.5
1.0
1.5
0 6 12 18 24 6 12 18 24 6 12 18 24 6 12 18 24
11 dph
12 dph
13 dph
14 dph
9 dph
10 dph
zeitgeber time (h)

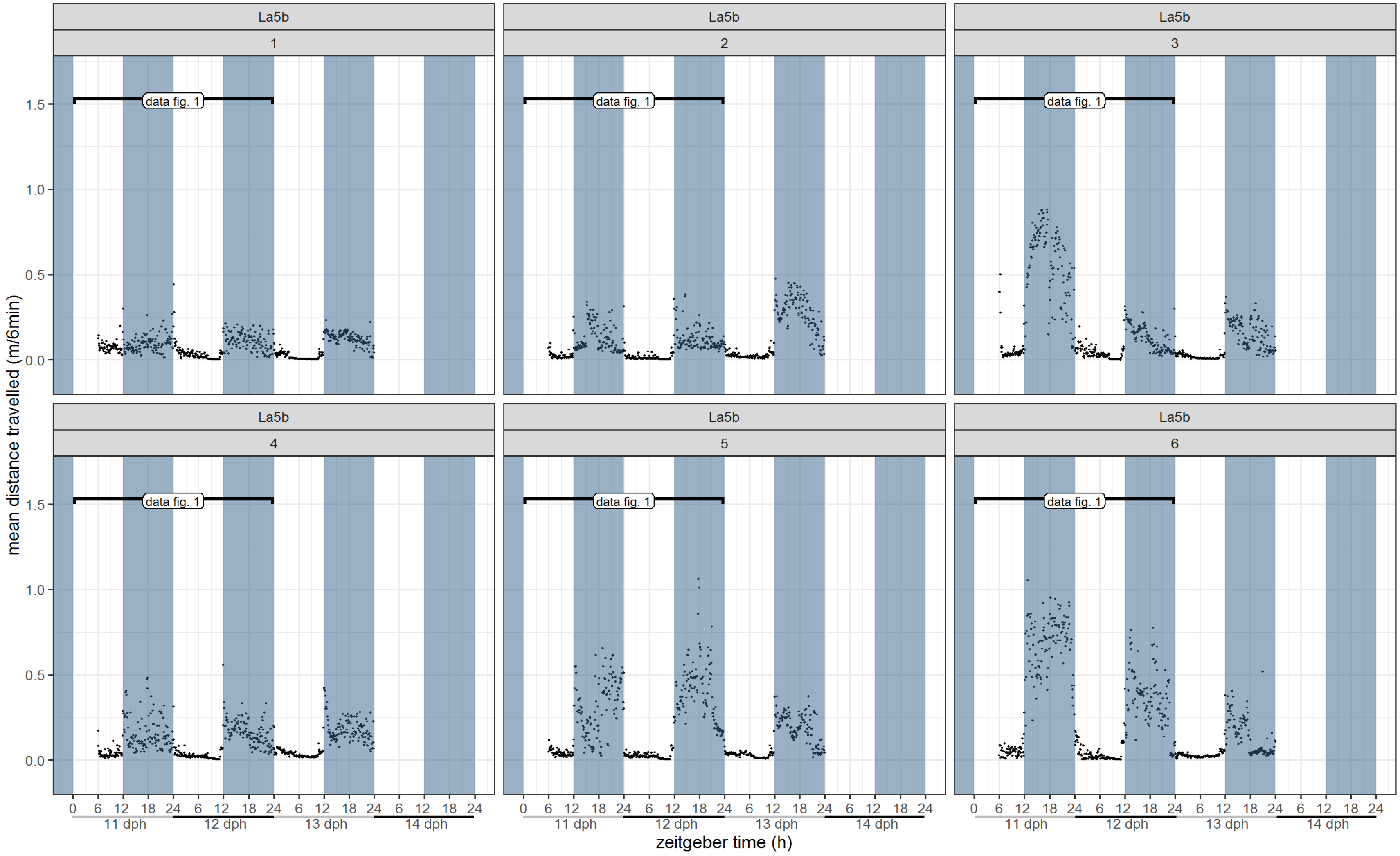
La5b
1
2
3
4
5
6
data fig. 1
mean distance travelled (m/6min)
0.0
0.5
1.0
1.5
0 6 12 18 24 6 12 18 24 6 12 18 24 6 12 18 24
11 dph
12 dph
13 dph
14 dph
zeitgeber time (h)

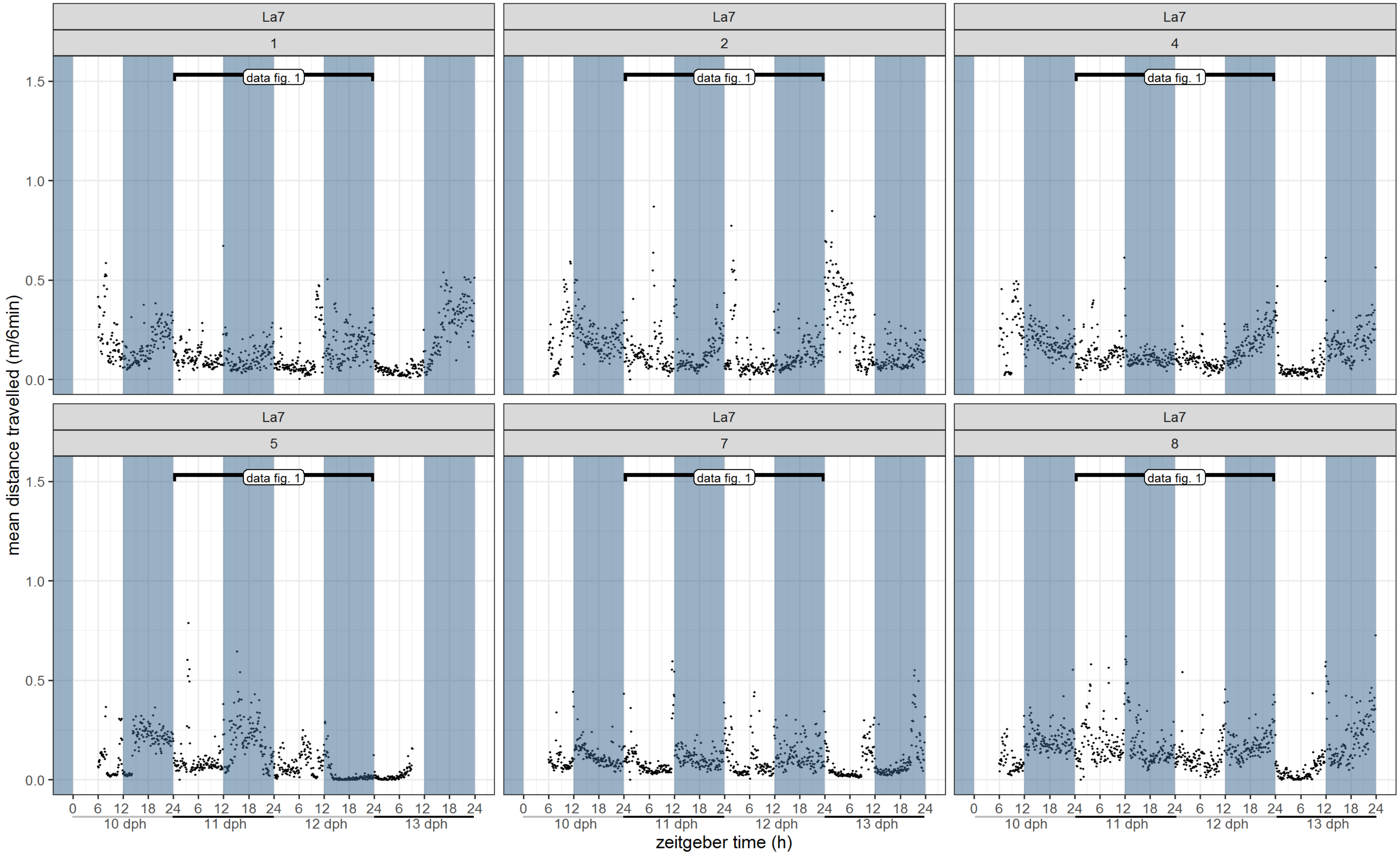
La7
1
2
4
5
7
8
data fig. 1
mean distance travelled (m/6min)
0.0
0.5
1.0
1.5
0 6 12 18 24 6 12 18 24 6 12 18 24 6 12 18 24
10 dph
11 dph
12 dph
13 dph
zeitgeber time (h)

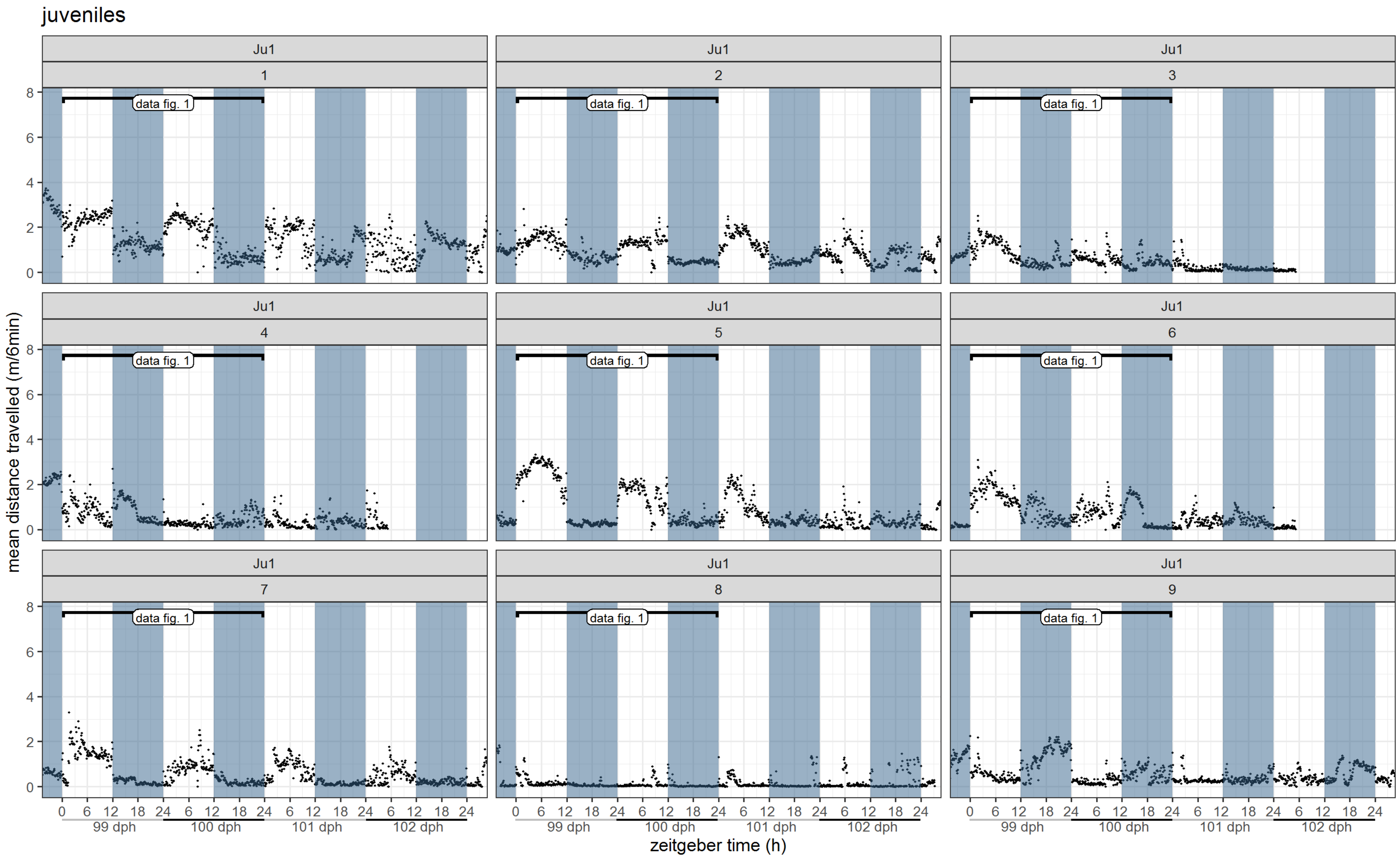

juveniles
Ju1
1
2
3
4
5
6
7
8
9
data fig. 1
mean distance travelled (m/6min)
0
2
4
6
8
0 6 12 18 24 6 12 18 24 6 12 18 24 6 12 18 24
99 dph
100 dph
101 dph
102 dph
zeitgeber time (h)

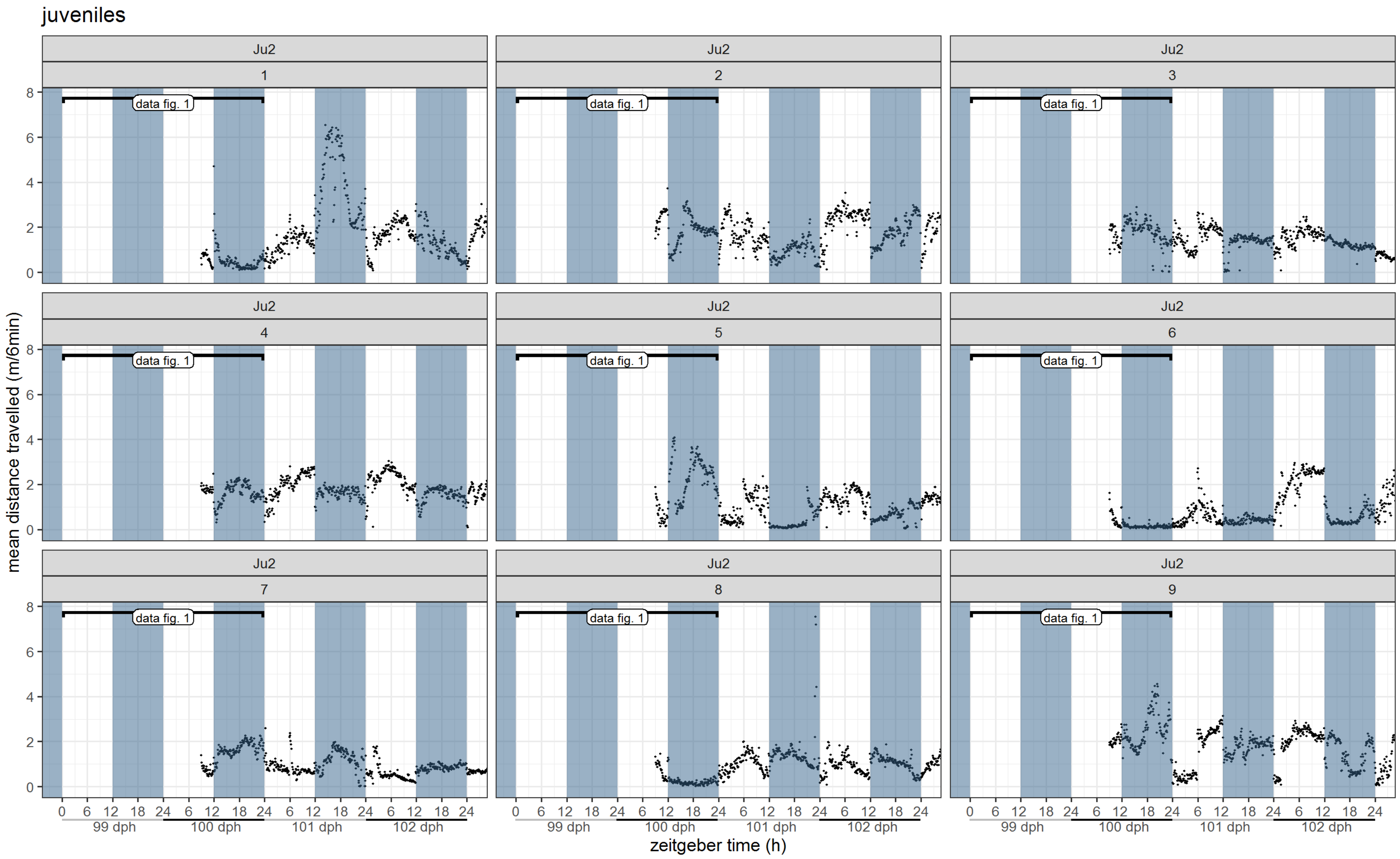

juveniles
Ju2
1
2
3
4
5
6
7
8
9
data fig. 1
mean distance travelled (m/6min)
0
2
4
6
8
0 6 12 18 24 6 12 18 24 6 12 18 24 6 12 18 24
99 dph
100 dph
101 dph
102 dph
zeitgeber time (h)

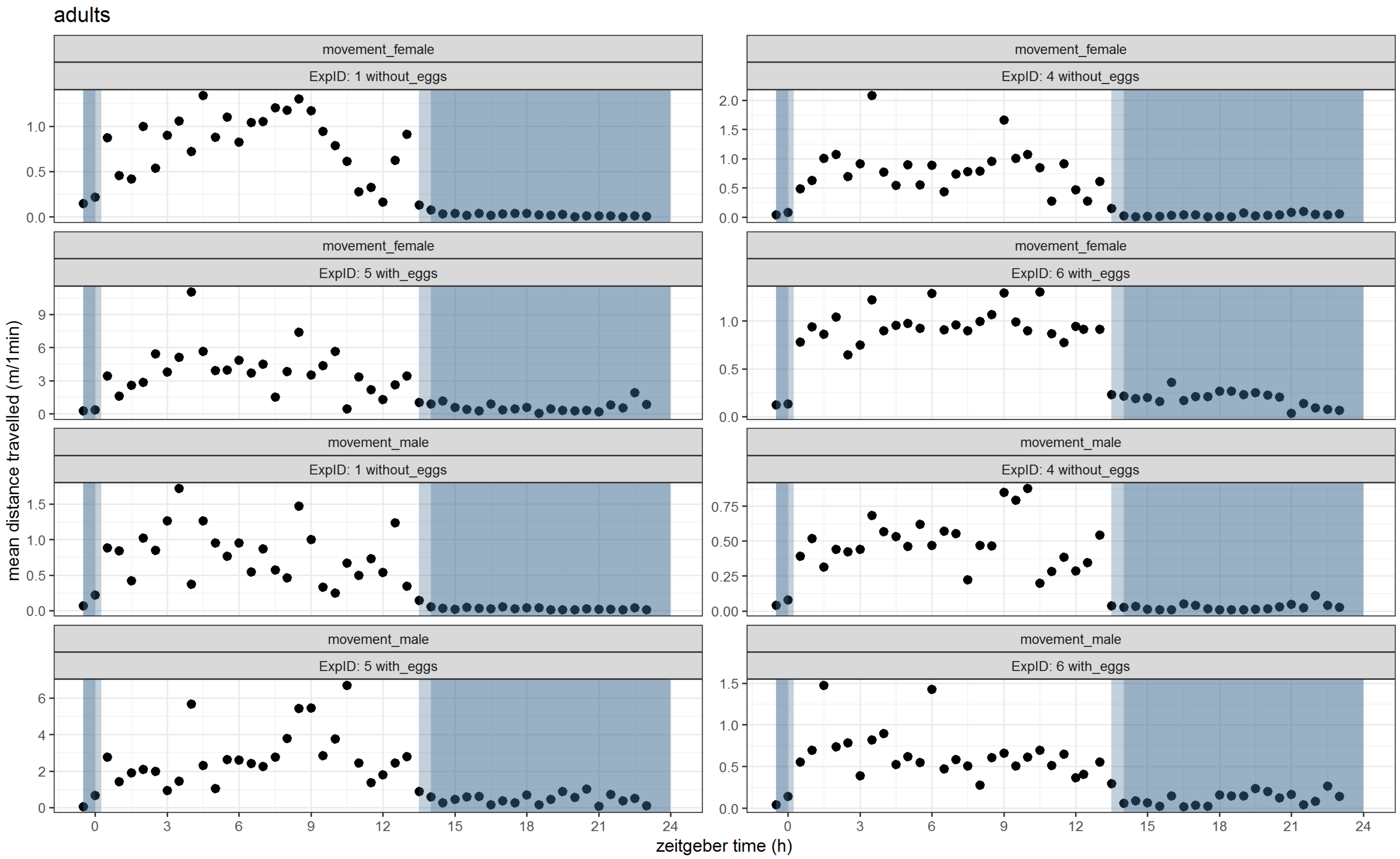
adults
movement_female
ExpID: 1 without_eggs
movement_female
ExpID: 4 without_eggs
movement_female
ExpID: 5 with_eggs
movement_female
ExpID: 6 with_eggs
movement_male
ExpID: 1 without_eggs
movement_male
ExpID: 4 without_eggs
movement_male
ExpID: 5 with_eggs
movement_male
ExpID: 6 with_eggs
mean distance travelled (m/1min)
zeitgeber time (h)
0
3
6
9
12
15
18
21
24

3 Supplementary table 1

	pVal	phase	peak.shape	period	sample type
clocka	0.001	9	12	24	larvae
bmal1	0.001	9	12	24	larvae
cry1b	0.001	3	12	24	larvae
per1b	0.000	21	18	24	larvae
per2	0.001	3	12	24	larvae
per3	0.000	3	12	24	larvae
cpd photolyase	0.003	3	12	24	larvae
6-4-photolyase	0.014	3	12	24	larvae
cry dash	0.000	3	12	24	larvae
clocka	0.023	9	18	24	juveniles
bmal1	0.013	15	6	24	juveniles
cry1b	0.000	3	12	24	juveniles
per1b	0.000	21	18	24	juveniles
per2	0.003	3	18	24	juveniles
per3	0.000	3	12	24	juveniles
cpd photolyase	0.006	3	12	24	juveniles
6-4-photolyase	0.000	3	12	24	juveniles
cry dash	0.000	3	12	24	juveniles
clocka	0.963	9	18	24	cells
bmal1	0.088	21	6	24	cells
cry1b	0.158	3	18	24	cells
per1b	0.417	3	12	24	cells
per2	0.040	3	12	24	cells
per3	0.004	3	12	24	cells
cpd photolyase	0.213	9	6	24	cells
6-4-photolyase	0.422	9	6	24	cells
cry dash	0.314	9	6	24	cells

4 Load necessary packages

```
#data sorting/data frame manipulation
library(reshape2)
library(tidyr)
library(dplyr)
library(scales)

#statistics
library(rain)
```

```
#graphics
library(ggplot2)
library(cowplot)
library(gridExtra)
library(magick)
library(ggforce)

## Warning: package 'ggforce' was built under R version 4.0.3
```

5 Gene expression of clock and photoreactivation DNA repair genes in larval and juvenile clownfish (figure 3)

5.1 Load data and adjust the data frame

Quantitative real time PCR was analysed for Cp value (using the 2nd derivative maximum method) and relative expression with LightCycler 480 Software, Version 1.5 (Roche) ("Target_Ref" in the data frame). The normalization was done in Excel separately for each sample group/experiment. The table was exported into csv and imported here.

```
#Load data
Relquant <- read.csv("./data/Relquant.csv", row.names=1, sep=";")

#sort the factors (important for order of the facets of the plot)
Relquant$Targets <- factor(Relquant$Targets, levels =
c("bmal1","clocka","cry1b","per1b","per2","per3","cpd photolyase","cry
dash","6-4-photolyase"))

#melt data from wide to long format and calculate mean/standard deviation for
larvae, juveniles and cells
mean_Relq <- cbind(

melt(tapply(Relquant$Normalized,list(Relquant$Targets,Relquant$samplegroup,Re
lquant$ZT,Relquant$References),mean)),

melt(tapply(Relquant$Normalized,list(Relquant$Targets,Relquant$samplegroup,Re
lquant$ZT,Relquant$References),sd))[5])

colnames(mean_Relq) <-
c("Targets","samplegroup","ZT","References","Target_ref_mean","sd")

#add grouping variables to differentiate between clock and photolyase genes
mean_Relq$gene_group <- gsub("bmal1", "clock",mean_Relq$Targets)
mean_Relq$gene_group <- gsub("clocka", "clock",mean_Relq$gene_group)
mean_Relq$gene_group <- gsub("cry1b", "clock",mean_Relq$gene_group)
mean_Relq$gene_group <- gsub("per1b", "clock",mean_Relq$gene_group)
mean_Relq$gene_group <- gsub("per2", "clock",mean_Relq$gene_group)
mean_Relq$gene_group <- gsub("per3", "clock",mean_Relq$gene_group)
```

```
mean_Relq$gene_group <- gsub("cpd photolyase",
"photolyase",mean_Relq$gene_group)
mean_Relq$gene_group <- gsub("cry dash", "photolyase",mean_Relq$gene_group)
mean_Relq$gene_group <- gsub("6-4-photolyase",
"photolyase",mean_Relq$gene_group)
```

5.2 Draw diagram

The diagram is included in the manuscript as figure 3.

```
#choose colors for the diagram
cbPalette <- c("coral", "darkblue")

#sort the factors for the diagram
mean_Relq$samplegroup <- factor(mean_Relq$samplegroup, levels =
c("larvae","juveniles","cells"))

#draw diagram
ggplot(data=mean_Relq)+
  facet_grid(vars(samplegroup),vars(Targets))+
  annotate('rect',xmin=12,xmax=24,ymin=-Inf,ymax=Inf,fill = "steelblue4",
           alpha = 0.4)+
  geom_point(aes(x=ZT,y=Target_ref_mean,color = gene_group),size = 1)+
  geom_line(aes(x=ZT,y=Target_ref_mean,color = gene_group))+
  geom_errorbar(aes(ymin=Target_ref_mean-sd, ymax=Target_ref_mean+sd,x=ZT),
                size=.2,width=1.5)+
  scale_y_continuous(breaks = c(seq(0,1,0.25)))+
  scale_x_continuous(breaks = c(3,9,15,21), limits = c(0,24))+
  scale_colour_manual(values=cbPalette)+
  ylab("normalized relative expression")+
  xlab("zeitgeber time (h)")+
  theme_bw(base_size = 8)+
  theme(legend.position="none",strip.text.x = element_text(face = "italic"))
```

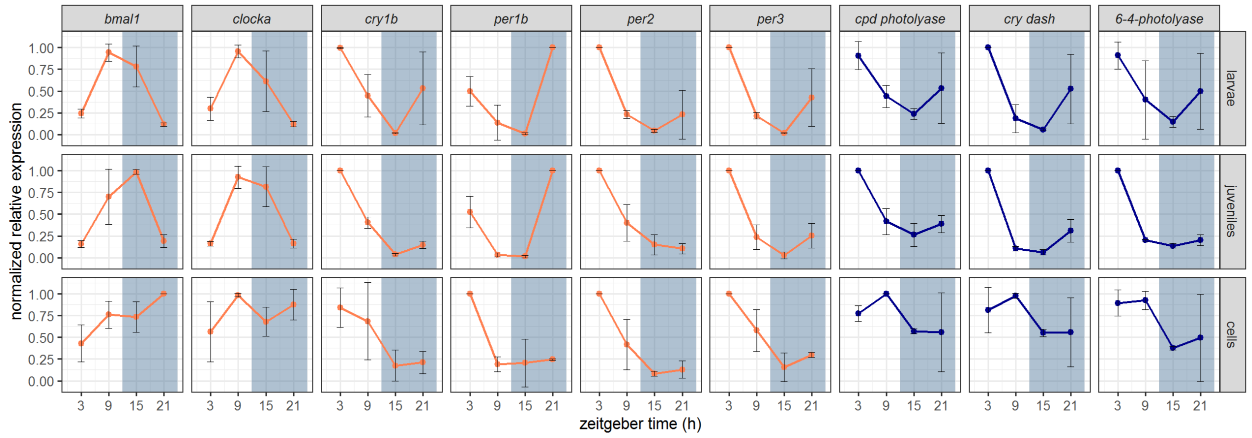


```
#save figure
ggsave("./results/gene_expression.pdf", width = 17, height = 6, units ="cm",
dpi = 600,device = "pdf")
```

5.3 Statistics (RAIN)

The statistical analysis was done with the RAIN package based on the normalized values. A data frame was prepared with one column per target gene. Each column contains the replicates for each time one after each other.

```
#Prepare data frame for analysis with RAIN, first for cells
cell_rain <- cbind(
  "clocka_cells" = subset(Relquant, samplegroup == "cells" & Targets ==
"clocka")[order(subset(Relquant, samplegroup == "cells" & Targets ==
"clocka")$Order),18],
  "bmal1_cells" = subset(Relquant, samplegroup == "cells" & Targets ==
"bmal1")[order(subset(Relquant, samplegroup == "cells" & Targets ==
"bmal1")$Order),18],
  "cry1b_cells" = subset(Relquant, samplegroup == "cells" & Targets ==
"cry1b")[order(subset(Relquant, samplegroup == "cells" & Targets ==
"cry1b")$Order),18],
  "per1b_cells" =subset(Relquant, samplegroup == "cells" & Targets ==
"per1b")[order(subset(Relquant, samplegroup == "cells" & Targets ==
"per1b")$Order),18],
  "per2_cells" = subset(Relquant, samplegroup == "cells" & Targets ==
"per2")[order(subset(Relquant, samplegroup == "cells" & Targets ==
"per2")$Order),18],
  "per3_cells" = subset(Relquant, samplegroup == "cells" & Targets ==
"per3")[order(subset(Relquant, samplegroup == "cells" & Targets ==
"per3")$Order),18],
  "cpd photolyase_cells" = subset(Relquant, samplegroup == "cells" & Targets
== "cpd photolyase")[order(subset(Relquant, samplegroup == "cells" & Targets
== "cpd photolyase")$Order),18],
  "6-4-photolyase_cells" = subset(Relquant, samplegroup == "cells" & Targets
== "6-4-photolyase")[order(subset(Relquant, samplegroup == "cells" & Targets
== "6-4-photolyase")$Order),18],
  "cry dash_cells" = subset(Relquant, samplegroup == "cells" & Targets ==
"cry dash")[order(subset(Relquant, samplegroup == "cells" & Targets == "cry
dash")$Order),18]
)

#Prepare data frame for larvae and juveniles
animals_rain <- cbind(
  "clocka_larvae" = subset(Relquant, samplegroup == "larvae" & Targets ==
"clocka")[order(subset(Relquant, samplegroup == "larvae" & Targets ==
"clocka")$Order),18],
  "bmal1_larvae" = subset(Relquant, samplegroup == "larvae" & Targets ==
"bmal1")[order(subset(Relquant, samplegroup == "larvae" & Targets ==
"bmal1")$Order),18],
  "cry1b_larvae" = subset(Relquant, samplegroup == "larvae" & Targets ==
"cry1b")[order(subset(Relquant, samplegroup == "larvae" & Targets ==
"cry1b")$Order),18],
  "per1b_larvae" =subset(Relquant, samplegroup == "larvae" & Targets ==
"per1b")[order(subset(Relquant, samplegroup == "larvae" & Targets ==
"per1b")$Order),18],
```

```
  "per2_larvae" = subset(Relquant, samplegroup == "larvae" & Targets ==
"per2")[order(subset(Relquant, samplegroup == "larvae" & Targets ==
"per2")$Order),18],
  "per3_larvae" = subset(Relquant, samplegroup == "larvae" & Targets ==
"per3")[order(subset(Relquant, samplegroup == "larvae" & Targets ==
"per3")$Order),18],
  "cpd photolyase_larvae" = subset(Relquant, samplegroup == "larvae" &
Targets == "cpd photolyase")[order(subset(Relquant, samplegroup == "larvae" &
Targets == "cpd photolyase")$Order),18],
  "6-4-photolyase_larvae" = subset(Relquant, samplegroup == "larvae" &
Targets == "6-4-photolyase")[order(subset(Relquant, samplegroup == "larvae" &
Targets == "6-4-photolyase")$Order),18],
  "cry dash_larvae" = subset(Relquant, samplegroup == "larvae" & Targets ==
"cry dash")[order(subset(Relquant, samplegroup == "larvae" & Targets == "cry
dash")$Order),18],

  "clocka_juveniles" = subset(Relquant, samplegroup == "juveniles" & Targets
== "clocka")[order(subset(Relquant, samplegroup == "juveniles" & Targets ==
"clocka")$Order),18],
  "bmal1_juveniles" = subset(Relquant, samplegroup == "juveniles" & Targets
== "bmal1")[order(subset(Relquant, samplegroup == "juveniles" & Targets ==
"bmal1")$Order),18],
  "cry1b_juveniles" = subset(Relquant, samplegroup == "juveniles" & Targets
== "cry1b")[order(subset(Relquant, samplegroup == "juveniles" & Targets ==
"cry1b")$Order),18],
  "per1b_juveniles" =subset(Relquant, samplegroup == "juveniles" & Targets ==
"per1b")[order(subset(Relquant, samplegroup == "juveniles" & Targets ==
"per1b")$Order),18],
  "per2_juveniles" = subset(Relquant, samplegroup == "juveniles" & Targets ==
"per2")[order(subset(Relquant, samplegroup == "juveniles" & Targets ==
"per2")$Order),18],
  "per3_juveniles" = subset(Relquant, samplegroup == "juveniles" & Targets ==
"per3")[order(subset(Relquant, samplegroup == "juveniles" & Targets ==
"per3")$Order),18],
  "cpd photolyase_juveniles" = subset(Relquant, samplegroup == "juveniles" &
Targets == "cpd photolyase")[order(subset(Relquant, samplegroup ==
"juveniles" & Targets == "cpd photolyase")$Order),18],
  "6-4-photolyase_juveniles" = subset(Relquant, samplegroup == "juveniles" &
Targets == "6-4-photolyase")[order(subset(Relquant, samplegroup ==
"juveniles" & Targets == "6-4-photolyase")$Order),18],
  "cry dash_juveniles" = subset(Relquant, samplegroup == "juveniles" &
Targets == "cry dash")[order(subset(Relquant, samplegroup == "juveniles" &
Targets == "cry dash")$Order),18]
)

#statistical analysis
rain(cell_rain ,deltat = 6, period = 24, nr.series = 2, method =
"independent") -> rain.result.cell
rain(animals_rain ,deltat = 6, period = 24, nr.series = 3, method =
"independent") -> rain.result.animal
```

```
#adapt acrophase to ZT of first timepoint by substracting 3h from phase
rain.result.cell$phase - 3 -> rain.result.cell$phase
rain.result.animal$phase - 3 -> rain.result.animal$phase

rain.result.cell

##                               pVal phase peak.shape period
## clocka_cells         0.963137472     9         18     24
## bmal1_cells          0.088137472    21          6     24
## cry1b_cells          0.157733898     3         18     24
## per1b_cells          0.416574279     3         12     24
## per2_cells           0.040396341     3         12     24
## per3_cells           0.003878702     3         12     24
## cpd photolyase_cells 0.212860310     9          6     24
## 6-4-photolyase_cells 0.421808886     9          6     24
## cry dash_cells       0.314174894     9          6     24

rain.result.animal

##                                    pVal phase peak.shape period
## clocka_larvae             1.038591e-03     9         12     24
## bmal1_larvae              1.038591e-03     9         12     24
## cry1b_larvae              8.497564e-04     3         12     24
## per1b_larvae              2.227336e-04    21         18     24
## per2_larvae               8.497564e-04     3         12     24
## per3_larvae               1.440265e-05     3         12     24
## cpd photolyase_larvae     2.512462e-03     3         12     24
## 6-4-photolyase_larvae     1.434664e-02     3         12     24
## cry dash_larvae           1.440265e-05     3         12     24
## clocka_juveniles          2.309257e-02     9         18     24
## bmal1_juveniles           1.315442e-02    15          6     24
## cry1b_juveniles           1.440265e-05     3         12     24
## per1b_juveniles           7.172778e-05    21         18     24
## per2_juveniles            3.488235e-03     3         18     24
## per3_juveniles            1.440265e-05     3         12     24
## cpd photolyase_juveniles 5.985101e-03     3         12     24
## 6-4-photolyase_juveniles 2.736504e-04     3         12     24
## cry dash_juveniles        3.344615e-04     3         12     24
```

6 Analysis of Behavioural Data

6.1 Calculate distance moved from position data

6.1.1 Larvae and Juveniles

The position of clownfish larvae and juveniles was tracked with Fishtracker. The position datasets include the x and y coordinate as separate column for each animal. Additionally, we added NAs for breaks during water exchange/feeding. Using the position data, the

distance larvae moved were calculated with the Pythagoras' theorem. The movement data was summed up for every 6 minutes, for what we set the following variable:

```
SumUp <- 360
```

If larvae died or were removed from the experiment, data was filled with NA during analysis.

Additionally, the zeitgeber time was added including the age of the animal for better overview. Therefore, we used the following function to add a column to the resulting data frame: It includes the age of the larvae (7~dph) and the offset, how many hours after the zeitgeber (light on) the experiment started (12~h).

```
offset <- 7 * 24 + 12
cbind(
  time = c(seq(offset, offset + length(RIS[,1])/2 - 0.5, 0.5)),
  RIS
) -> RIS
```

6.1.1.1 Larvae 1

```
#load data table
read.table("./position/larvae1.txt", sep=" ") -> tab_13

## Calculate distance animals travelled per second
  #number of columns
  length(tab_13[1,]) -> coln
  #count rows
  length(tab_13[,1]) -> rown

  #make new matrix with the same number of rows but half of the columns
  #will be result table
  result<-matrix(NA,nrow=rown,ncol=coln/2)

  x <- 1                              #counter for the columns
  t <- 1                              #counter for the columns (result)
  for (x in seq(1,coln,2)){
    z <- 1                        #counter for rows
    for (z in 1:rown)   {
      tab_13[z,x] -> X1
      tab_13[z+1,x] -> X2
      tab_13[z,x+1] -> Y1
      tab_13[z+1,x+1] -> Y2
      pitagora <- sqrt((X1-X2)^2 + (Y1-Y2)^2)
      pitagora -> result[z,t]
      z <- z+1                    #go one row down
    }
    t <- t+1
  }
```

```
#delete last row and save as tab_9
result[-(length(result[,1])),] -> tab_9

#adapt beginning to full hour by deleting the first minutes
tab_9 <- tab_9[-c(1:906),]

##sum up the distance larvae travelled per hour
  lr <- length(tab_9[,1])     #count rows of tab_9
  len <- lr/SumUp            #how many groups - adding up every x rows
  i = 1
  s = 1

  #prepare data frame
  RIS <- matrix(ncol=6, nrow=(len+1))
  for (i in 1: len) {
    print(s)
    print(i)
    if (s+SumUp < lr)   {
      apply(tab_9[(s:(s+(SumUp-1))), ], 2 , sum,na.rm=TRUE) -> RIS[i, ]
      RIS
      i = i+1
      s = s+SumUp
      print(s)
    }
    if (s+SumUp > lr)   {
      print(i)
      apply(tab_9[(s:lr), ], 2 , sum) -> RIS[i, ]
      RIS;
    }
  }

#add zeitgeber time (h) and age (days) by adding the starting timepoint.
  offset <- 7*24 + 12
cbind(
  time = c(seq(offset, offset + length(RIS[,1])/10 - 0.1 ,0.1)),
  RIS
) -> RIS

colnames(RIS)[c(2:7)]<-c("La1.1","La1.2","La1.3","La1.4","La1.5","La1.6")

#save as table
write.table(RIS,"./results/larvae1_RIS_6min.txt", quote=F, row.names=F)
```

6.1.1.2 Larvae 2

```
#load data table
read.table("./position/larvae2.txt", sep=" ") -> tab_13

## Calculate distance animals travelled per second
  #number of columns
  length(tab_13[1,]) -> coln
  #count rows
```

```
  length(tab_13[,1]) -> rown

  #make new matrix with the same number of rows but half of the columns
  #will be result table
  result<-matrix(NA,nrow=rown,ncol=coln/2)

  x <- 1                          #counter for the columns
  t <- 1                          #counter for the columns (result)
  for (x in seq(1,coln,2)){
    z <- 1                        #counter for rows
    for (z in 1:rown)   {
      tab_13[z,x] -> X1
      tab_13[z+1,x] -> X2
      tab_13[z,x+1] -> Y1
      tab_13[z+1,x+1] -> Y2
      pitagora <- sqrt((X1-X2)^2 + (Y1-Y2)^2)
      pitagora -> result[z,t]
      z <- z+1                    #go one row down
    }
    t <- t+1
  }

#delete last row and save as tab_9
result[-(length(result[,1])),] -> tab_9

#adapt beginning to full hour by deleting the first minutes
tab_9 <- tab_9[-c(1:1694),]

###sum up the distance larvae travelled per hour
  lr <- length(tab_9[,1])      #count rows of tab_9
  len <- lr/SumUp               #how many groups - adding up every x rows
  i = 1
  s = 1

  #prepare data frame
  RIS <- matrix(ncol=6, nrow=(len+1))
  for (i in 1: len) {
    print(s)
    print(i)
    if (s+SumUp < lr)   {
      apply(tab_9[(s:(s+(SumUp-1))), ], 2 , sum,na.rm=TRUE) -> RIS[i, ]
      RIS
      i = i+1
      s = s+SumUp
      print(s)
    }
    if (s+SumUp > lr)   {
      print(i)
      apply(tab_9[(s:lr), ], 2 , sum) -> RIS[i, ]
      RIS;
    }
```

```r
  }

#add zeitgeber time (h) and age (days) by adding the starting timepoint.
  offset <- 15*24 + 11
cbind(
  time = c(seq(offset, offset + length(RIS[,1])/10 - 0.1 ,0.1)),
  RIS
) -> RIS

#fill data for dead larvae with NA
RIS[(RIS[,1] > (15 + 2.751) * 24 + 11),5]<-NA #delete dead larvae
RIS[(RIS[,1] < (15 + 0.7625) * 24 + 11),7]<-NA #dead larvae was replaced with a new one

colnames(RIS)[c(2:7)]<-c("La2.1","La2.2","La2.3","La2.4","La2.5","La2.6")

#save as table
write.table(RIS,"./results/larvae2_RIS_6min.txt", quote=F, row.names=F)
```

6.1.1.3 Larvae 3

```r
#load data table
read.table("./position/larvae3.txt", sep=" ") -> tab_13

## Calculate distance animals travelled per second
  #number of columns
  length(tab_13[1,]) -> coln
  #count rows
  length(tab_13[,1]) -> rown

  #make new matrix with the same number of rows but half of the columns
  #will be result table
  result<-matrix(NA,nrow=rown,ncol=coln/2)

  x <- 1                              #counter for the columns
  t <- 1                              #counter for the columns (result)
  for (x in seq(1,coln,2)){
    z <- 1                        #counter for rows
    for (z in 1:rown)   {
      tab_13[z,x] -> X1
      tab_13[z+1,x] -> X2
      tab_13[z,x+1] -> Y1
      tab_13[z+1,x+1] -> Y2
      pitagora <- sqrt((X1-X2)^2 + (Y1-Y2)^2)
      pitagora -> result[z,t]
      z <- z+1                    #go one row down
    }
    t <- t+1
  }

#delete last row and save as tab_9
```

```r
result[-(length(result[,1])),] -> tab_9

#adapt beginning to full hour by deleting the first minutes
tab_9 <- tab_9[-c(1:364),]

##sum up the distance larvae travelled per hour
  lr <- length(tab_9[,1])     #count rows of tab_9
  len <- lr/SumUp           #how many groups - adding up every x rows
  #len
  i = 1
  s = 1

  #prepare data frame
  RIS <- matrix(ncol=6, nrow=(len+1))
  for (i in 1: len) {
    print(s)
    print(i)
    if (s+SumUp < lr)   {
      apply(tab_9[(s:(s+(SumUp-1))), ], 2 , sum,na.rm=TRUE) -> RIS[i, ]
      RIS
      i = i+1
      s = s+SumUp
      print(s)
    }
    if (s+SumUp > lr)   {
      print(i)
      apply(tab_9[(s:lr), ], 2 , sum) -> RIS[i, ]
      RIS;
    }
  }

#add zeitgeber time (h) and age (days) by adding the starting timepoint.
  offset <- 12 * 24 + 2
cbind(
  time = c(seq(offset, offset + length(RIS[,1])/10 - 0.1 ,0.1)),
  RIS
) -> RIS

#fill data for dead larvae with NA
RIS[(RIS[,1] > (12 + 3.9896) * 24 + 2),7]<-NA #remove dead larvae
RIS[(RIS[,1] > (12 + 5.152) * 24 + 2),2]<-NA #remove dead larvae
RIS[(RIS[,1] > (12 + 6.198) * 24 + 2),c(3,6)]<-NA #remove dead larvae

colnames(RIS)[c(2:7)]<-c("La3.1","La3.2","La3.3","La3.4","La3.5","La3.6")

#save as table
write.table(RIS,"./results/larvae3_RIS_6min.txt", quote=F, row.names=F)
```

6.1.1.4 Larvae 4

For this experiment larvae of two different ages were used, therefore the data was split into larvae4a and 4b after calculation of distance moved.

```
#load data table
read.table("./position/larvae4.txt", sep=" ") -> tab_13

## Calculate distance animals travelled per second
  #number of columns
  length(tab_13[1,]) -> coln
  #count rows
  length(tab_13[,1]) -> rown

  #make new matrix with the same number of rows but half of the columns
  #will be result table
  result<-matrix(NA,nrow=rown,ncol=coln/2)

  x <- 1                          #counter for the columns
  t <- 1                          #counter for the columns (result)
  for (x in seq(1,coln,2)){
    z <- 1                    #counter for rows
    for (z in 1:rown)   {
      tab_13[z,x] -> X1
      tab_13[z+1,x] -> X2
      tab_13[z,x+1] -> Y1
      tab_13[z+1,x+1] -> Y2
      pitagora <- sqrt((X1-X2)^2 + (Y1-Y2)^2)
      pitagora -> result[z,t]
      z <- z+1                #go one row down
    }
    t <- t+1
  }

#delete last row and save as tab_9
result[-(length(result[,1])),] -> tab_9

#adapt beginning to full hour by deleting the first minutes
tab_9 <- tab_9[-c(1:174),]

##sum up the distance larvae travelled per hour
  lr <- length(tab_9[,1])     #count rows of tab_9
  len <- lr/SumUp             #how many groups - adding up every x rows
  #len
  i = 1
  s = 1

  #prepare data frame
  RIS <- matrix(ncol=6, nrow=(len+1))
  for (i in 1: len) {
    print(s)
```

```
    print(i)
    if (s+SumUp < lr)   {
      apply(tab_9[(s:(s+(SumUp-1))), ], 2 , sum,na.rm=TRUE) -> RIS[i, ]
      RIS
      i = i+1
      s = s+SumUp
      print(s)
    }
    if (s+SumUp > lr)   {
      print(i)
      apply(tab_9[(s:lr), ], 2 , sum) -> RIS[i, ]
      RIS;
    }
  }

#add zeitgeber time (h) and age (days) by adding the starting timepoint.
Because different aged larvae were observed, data was split.
  offset <- 11*24 + 7
  cbind(
    time = c(seq(offset, offset + length(RIS[,1])/10 - 0.1 ,0.1)),
    RIS[,c(1:3)]
  ) -> RIS_a

  offset <- 8*24 + 7
  cbind(
    time = c(seq(offset, offset + length(RIS[,1])/10 - 0.1 ,0.1)),
    RIS[,c(4:6)]
  ) -> RIS_b

#fill data for dead larvae with NA

RIS_a[(RIS_a[,1] < (11+0.8618)*24 + 7),4]<-NA #delete dead larvae

RIS_b[(RIS_b[,1] > (8+2.992)*24 + 7),2]<-NA #delete dead larvae
RIS_b[(RIS_b[,1] > (8+3.866)*24 + 7),3]<-NA #delete dead larvae

colnames(RIS_a)[c(2:4)]<-c("La4a.1","La4a.2","La4a.3")
colnames(RIS_b)[c(2:4)]<-c("La4b.1","La4b.2","La4b.3")

#save as table
write.table(RIS_a,"./results/larvae4a_RIS_6min.txt", quote=F, row.names=F)
write.table(RIS_b,"./results/larvae4b_RIS_6min.txt", quote=F, row.names=F)
```

6.1.1.5 Larvae 5

For this experiment the larvae were recorded with Yawcam and occasionally frames were dropped. We used the timestamps to correct for it. Therefore, we converted the timestamps into a sequence of numbers from 0 to 613664, where the numbers of the frames were missing. We combined it with the calculated distance and by merging it with a consecutive

sequence from 0 to 613664 we were able to add NAs were necessary. Within the experiment we observed different aged larvae and therefore we separated the data into larvae5a and b.

```
#load data table
read.table("./position/larvae5.txt", sep=" ") -> tab_13

## Calculate distance animals travelled per second
  #number of columns
  length(tab_13[1,]) -> coln
  #count rows
  length(tab_13[,1]) -> rown

  #make new matrix with the same number of rows but half of the columns
  #will be result table
  result<-matrix(NA,nrow=rown,ncol=coln/2)

  x <- 1                              #counter for the columns
  t <- 1                              #counter for the columns (result)
  for (x in seq(1,coln,2)){
    z <- 1                        #counter for rows
    for (z in 1:rown)   {
      tab_13[z,x] -> X1
      tab_13[z+1,x] -> X2
      tab_13[z,x+1] -> Y1
      tab_13[z+1,x+1] -> Y2
      pitagora <- sqrt((X1-X2)^2 + (Y1-Y2)^2)
      pitagora -> result[z,t]
      z <- z+1                    #go one row down
    }
    t <- t+1
  }

#delete last row and save as tab_9
result[-(length(result[,1])),] -> tab_9

#adapt beginning to full hour by deleting the first minutes
#not necessary, it started at full hour

#problem: sometimes a frame is dropped by the programme - we have to correct
for it:
  time <- read.table("./position/time_larvae5.txt", quote="\"",
comment.char="")
  cbind(time,tab_9) -> tab_9
  data.frame(1:max(tab_9$V1)) -> tmp
  colnames(tab_9)[1]<-"x"
  colnames(tmp)<-"x"
  left_join(tmp, tab_9, by = "x") -> tab_9
  tab_9[,-1] -> tab_9
```

```
##sum up the distance larvae travelled per hour
  lr <- length(tab_9[,1])     #count rows of tab_9
  len <- lr/SumUp           #how many groups - adding up every x rows
  #len
  i = 1
  s = 1

  #prepare data frame
  RIS <- matrix(ncol=12, nrow=(len+1))
  for (i in 1: len) {
    print(s)
    print(i)
    if (s+SumUp < lr)   {
      apply(tab_9[(s:(s+(SumUp-1))), ], 2 , sum,na.rm=TRUE) -> RIS[i, ]
      RIS
      i = i+1
      s = s+SumUp
      print(s)
    }
    if (s+SumUp > lr)   {
      print(i)
      apply(tab_9[(s:lr), ], 2 , sum) -> RIS[i, ]
      RIS;
    }
  }

#add zeitgeber time (h) and age (days) by adding the starting timepoint.
Because different aged larvae were observed, data was split.
  offset <- 14 * 24 + 6
  cbind(
    time = c(seq(offset, offset + length(RIS[,1])/10 - 0.1 ,0.1)),
    RIS[,c(1:6)]
  ) -> RIS_a

  offset <- 11 * 24 + 6
  cbind(
    time = c(seq(offset, offset + length(RIS[,1])/10 - 0.1 ,0.1)),
    RIS[,c(7:12)]
  ) -> RIS_b

#fill data for dead larvae with NA
RIS_a[(RIS_a[,1] > (14+1.0945)*24 +6),3]<-NA #delete dead larva
RIS_a[(RIS_a[,1] > (14+4.8258)*24 +6),c(4,5)]<-NA #delete dead larvae
RIS_a[(RIS_a[,1] > (14+6.06)*24 +6),6]<-NA #delete dead larva

RIS_b[(RIS_b[,1] > (11+4.83)*24 + 6),c(2,7,4)]<-NA #delete dead larvae
RIS_b[(RIS_b[,1] > (11+6.06)*24 + 6),5]<-NA #delete dead larva

colnames(RIS_a)[c(2:7)]<-
c("La5a.1","La5a.2","La5a.3","La5a.4","La5a.5","La5a.6")
colnames(RIS_b)[c(2:7)]<-
```

```
c("La5b.1","La5b.2","La5b.3","La5b.4","La5b.5","La5b.6")

#save as table
write.table(RIS_a,"./results/larvae5a_RIS_6min.txt", quote=F, row.names=F)
write.table(RIS_b,"./results/larvae5b_RIS_6min.txt", quote=F, row.names=F)
```

6.1.1.6 Larvae 7

```
#load data table
read.table("./position/larvae7.txt", sep=" ") -> tab_13

## Calculate distance animals travelled per second
  #number of columns
  length(tab_13[1,]) -> coln
  #count rows
  length(tab_13[,1]) -> rown

  #make new matrix with the same number of rows but half of the columns
  #will be result table
  result<-matrix(NA,nrow=rown,ncol=coln/2)

  x <- 1                        #counter for the columns
  t <- 1                        #counter for the columns (result)
  for (x in seq(1,coln,2)){
    z <- 1                    #counter for rows
    for (z in 1:rown)   {
      tab_13[z,x] -> X1
      tab_13[z+1,x] -> X2
      tab_13[z,x+1] -> Y1
      tab_13[z+1,x+1] -> Y2
      pitagora <- sqrt((X1-X2)^2 + (Y1-Y2)^2)
      pitagora -> result[z,t]
      z <- z+1                #go one row down
    }
    t <- t+1
  }

#delete last row and save as tab_9
result[-(length(result[,1])),] -> tab_9

#adapt beginning to full hour by deleting the first minutes
tab_9 <- tab_9[-c(1:1655),] #removes 27min and 35s

##sum up the distance larvae travelled per hour
  lr <- length(tab_9[,1])     #count rows of tab_9
  len <- lr/SumUp             #how many groups - adding up every x rows
  #len
  i = 1
  s = 1

  #prepare data frame
```

```
  RIS <- matrix(ncol=9, nrow=(len+1))
  for (i in 1: len) {
    print(s)
    print(i)
    if (s+SumUp < lr)   {
      apply(tab_9[(s:(s+(SumUp-1))), ], 2 , sum,na.rm=TRUE) -> RIS[i, ]
      RIS
      i = i+1
      s = s+SumUp
      print(s)
    }
    if (s+SumUp > lr)   {
      print(i)
      apply(tab_9[(s:lr), ], 2 , sum) -> RIS[i, ]
      RIS;
    }
  }

#add zeitgeber time (h) and age (days) by adding the starting timepoint.
  offset <- 10 * 24 + 6
cbind(
  time = c(seq(offset, offset + length(RIS[,1])/10 - 0.1 ,0.1)),
  RIS
) -> RIS

#fill data for dead larvae with NA
RIS[,c(4,7,10)] <-NA #not 24 h of observation
RIS[(RIS[,1] > (10 + 3.13) * 24 + 6),6]<-NA

colnames(RIS)[c(2:10)]<-
c("La7.1","La7.2","La7.3","La7.4","La7.5","La7.6","La7.7","La7.8","La7.9")

#save as table
write.table(RIS,"./results/larvae7_RIS_6min.txt", quote=F, row.names=F)
```

6.1.1.7 Juvenile 1

The first experiment with juveniles were recorded using Yawcam and that is why occasionally frames were dropped. See Larvae 5 (4.1.1.5) for additional comments.

```
#load data table
read.table("./position/juvenile1.txt", sep=" ") -> tab_13

## Calculate distance animals travelled per second
  #number of columns
  length(tab_13[1,]) -> coln
  #count rows
  length(tab_13[,1]) -> rown

  #make new matrix with the same number of rows but half of the columns
  #will be result table
```

```r
  result<-matrix(NA,nrow=rown,ncol=coln/2)

  x <- 1                              #counter for the columns
  t <- 1                              #counter for the columns (result)
  for (x in seq(1,coln,2)){
    z <- 1                        #counter for rows
    for (z in 1:rown)   {
      tab_13[z,x] -> X1
      tab_13[z+1,x] -> X2
      tab_13[z,x+1] -> Y1
      tab_13[z+1,x+1] -> Y2
      pitagora <- sqrt((X1-X2)^2 + (Y1-Y2)^2)
      pitagora -> result[z,t]
      z <- z+1                    #go one row down
    }
    t <- t+1
  }

#delete last row (NA) and save as tab_9
as.data.frame(result[-(length(result[,1])),]) -> tab_9

#Problem: sometimes a frame is dropped by the programme - We have to correct
for it:
  time <- read.table("./position/time_juv1.txt", quote="\"", comment.char="")
  cbind(time,tab_9) -> tab_9
  data.frame(1:max(tab_9[,1])) -> tmp
  colnames(tab_9)[1]<-"x"
  colnames(tmp)<-"x"
  left_join(tmp, tab_9, by = "x") -> tab_9
  tab_9[,-1] -> tab_9

#adapt beginning to full hour by deleting the first minutes
tab_9 <- tab_9[-c(1:1200),]

##sum up the distance larvae travelled per hour
  lr <- length(tab_9[,1])      #count rows of tab_9
  len <- lr/SumUp             #how many groups - adding up every x rows
  #len
  i = 1
  s = 1

  #prepare data frame
  RIS <- matrix(ncol=9, nrow=(len+1))
  for (i in 1: len) {
    print(s)
    print(i)
    if (s+SumUp < lr)   {
      apply(tab_9[(s:(s+(SumUp-1))), ], 2 , sum,na.rm=TRUE) -> RIS[i, ]
      RIS
      i = i+1
      s = s+SumUp
```

```
      print(s)
    }
    if (s+SumUp > lr)   {
      print(i)
      apply(tab_9[(s:lr), ], 2 , sum) -> RIS[i, ]
      RIS;
    }
  }

#add zeitgeber time (h) and age (days) by adding the starting timepoint.
  offset <- 98 * 24 + 9
cbind(
  time = c(seq(offset, offset + length(RIS[,1])/10 - 0.1 ,0.1)),
  RIS
) -> RIS

#fill data for dead juveniles with NA
RIS[(RIS[,1] > (98 + 3.84375) * 24 + 9),c(4,5,7)]<-NA
RIS[(RIS[,1] > (98 + 4.76453) * 24 + 9),9]<-NA

colnames(RIS)[c(2:10)]<-
c("Ju1.1","Ju1.2","Ju1.3","Ju1.4","Ju1.5","Ju1.6","Ju1.7","Ju1.8","Ju1.9")

#save as table
write.table(RIS,file="./results/juvenile1_RIS_6min.txt")
```

6.1.1.8 Juvenile 2

```
#load data table
read.table("./position/juvenile2.txt", sep=" ") -> tab_13

## Calculate distance animals travelled per second
  #number of columns
  length(tab_13[1,]) -> coln
  #count rows
  length(tab_13[,1]) -> rown

  #make new matrix with the same number of rows but half of the columns
  #will be result table
  result<-matrix(NA,nrow=rown,ncol=coln/2)

  x <- 1                        #counter for the columns
  t <- 1                        #counter for the columns (result)
  for (x in seq(1,coln,2)){
    z <- 1                    #counter for rows
    for (z in 1:rown)   {
      tab_13[z,x] -> X1
      tab_13[z+1,x] -> X2
      tab_13[z,x+1] -> Y1
      tab_13[z+1,x+1] -> Y2
      pitagora <- sqrt((X1-X2)^2 + (Y1-Y2)^2)
      pitagora -> result[z,t]
```

```r
      z <- z+1              #go one row down
    }
    t <- t+1
  }

#delete last row and save as tab_9
result[-(length(result[,1])),] -> tab_9

#adapt beginning to full hour by deleting the first minutes
tab_9 <- tab_9[-c(1:506),]

##sum up the distance larvae travelled per hour
  lr <- length(tab_9[,1])     #count rows of tab_9
  len <- lr/SumUp           #how many groups - adding up every x rows
  #len
  i = 1
  s = 1

  #prepare data frame
  RIS <- matrix(ncol=9, nrow=(len+1))
  for (i in 1: len) {
    print(s)
    print(i)
    if (s+SumUp < lr)   {
      apply(tab_9[(s:(s+(SumUp-1))), ], 2 , sum,na.rm=TRUE) -> RIS[i, ]
      RIS
      i = i+1
      s = s+SumUp
      print(s)
    }
    if (s+SumUp > lr)   {
      print(i)
      apply(tab_9[(s:lr), ], 2 , sum) -> RIS[i, ]
      RIS;
    }
  }

#add zeitgeber time (h) and age (days) by adding the starting timepoint.
  offset <- 100*24 + 9
cbind(
  time = c(seq(offset, offset + length(RIS[,1])/10 - 0.1 ,0.1)),
  RIS
) -> RIS

colnames(RIS)[c(2:10)]<-
c("Ju2.1","Ju2.2","Ju2.3","Ju2.4","Ju2.5","Ju2.6","Ju2.7","Ju2.8","Ju2.9")

#save as table
write.table(RIS,"./results/juvenile2_RIS_6min.txt", quote=F, row.names=F)
```

6.1.1.9 Juvenile 3

```
#load data table
read.table("./position/juvenile3.txt", sep=" ") -> tab_13

## Calculate distance animals travelled per second
  #number of columns
  length(tab_13[1,]) -> coln
  #count rows
  length(tab_13[,1]) -> rown

  #make new matrix with the same number of rows but half of the columns
  #will be result table
  result<-matrix(NA,nrow=rown,ncol=coln/2)

  x <- 1                         #counter for the columns
  t <- 1                         #counter for the columns (result)
  for (x in seq(1,coln,2)){
    z <- 1                     #counter for rows
    for (z in 1:rown)   {
      tab_13[z,x] -> X1
      tab_13[z+1,x] -> X2
      tab_13[z,x+1] -> Y1
      tab_13[z+1,x+1] -> Y2
      pitagora <- sqrt((X1-X2)^2 + (Y1-Y2)^2)
      pitagora -> result[z,t]
      z <- z+1               #go one row down
    }
    t <- t+1
  }

#delete last row and save as tab_9
result[-(length(result[,1])),] -> tab_9

#adapt beginning to full hour by deleting the first minutes
tab_9 <- tab_9[-c(1:803),]

##sum up the distance larvae travelled per hour
  lr <- length(tab_9[,1])     #count rows of tab_9
  len <- lr/SumUp             #how many groups - adding up every x rows
  #len
  i = 1
  s = 1

  #prepare data frame
  RIS <- matrix(ncol=9, nrow=(len+1))
  for (i in 1: len) {
    print(s)
    print(i)
    if (s+SumUp < lr)   {
      apply(tab_9[(s:(s+(SumUp-1))), ], 2 , sum,na.rm=TRUE) -> RIS[i, ]
```

```
      RIS
      i = i+1
      s = s+SumUp
      print(s)
    }
    if (s+SumUp > lr)   {
      print(i)
      apply(tab_9[(s:lr), ], 2 , sum) -> RIS[i, ]
      RIS;
    }
  }

#add zeitgeber time (h) and age (days) by adding the starting timepoint.
  offset <- 52 * 24 + 9
cbind(
  time = c(seq(offset, offset + length(RIS[,1])/10 - 0.1 ,0.1)),
  RIS
) -> RIS

#fill data for dead juveniles with NA
RIS[(RIS[,1] > (52 + 3) * 24 + 9),3]<-NA

colnames(RIS)[c(2:10)]<-
c("Ju3.1","Ju3.2","Ju3.3","Ju3.4","Ju3.5","Ju3.6","Ju3.7","Ju3.8","Ju3.9")

#Due to software problems, one video was recorded with unknown fps and
therefore data after this timepoint was excluded to avoid errors in
statistical analysis.
RIS <- RIS[c(RIS[,1] < 100.0333 + 52*24+9),]

#save as table
write.table(RIS,"./results/juvenile3_RIS_6min.txt", quote=F, row.names=F)
```

6.1.1.10 Juvenile 4

```
#load data table
read.table("./position/juvenile4.txt", sep=" ") -> tab_13

## Calculate distance animals travelled per second
  #number of columns
  length(tab_13[1,]) -> coln
  #count rows
  length(tab_13[,1]) -> rown

  #make new matrix with the same number of rows but half of the columns
  #will be result table
  result<-matrix(NA,nrow=rown,ncol=coln/2)

  x <- 1                              #counter for the columns
  t <- 1                              #counter for the columns (result)
  for (x in seq(1,coln,2)){
```

```
    z <- 1                  #counter for rows
    for (z in 1:rown)   {
      tab_13[z,x] -> X1
      tab_13[z+1,x] -> X2
      tab_13[z,x+1] -> Y1
      tab_13[z+1,x+1] -> Y2
      pitagora <- sqrt((X1-X2)^2 + (Y1-Y2)^2)
      pitagora -> result[z,t]
      z <- z+1              #go one row down
    }
    t <- t+1
  }

#delete last row and save as tab_9
result[-(length(result[,1])),] -> tab_9

#The video capture programme stopped working for two occasions during this
observations (the recording was frozen). We excluded the whole time to avoid
errors in statistical analysis. Additionally, we adapted it to start at full
hour.
tab_9 <- tab_9[-c(1:262764),] #Removes the first 3d and ~1h

##sum up the distance larvae travelled per hour
  lr <- length(tab_9[,1])     #count rows of tab_9
  len <- lr/SumUp           #how many groups - adding up every x rows
  #len
  i = 1
  s = 1

  #prepare data frame
  RIS <- matrix(ncol=9, nrow=(len+1))
  for (i in 1: len) {
    print(s)
    print(i)
    if (s+SumUp < lr)   {
      apply(tab_9[(s:(s+(SumUp-1))), ], 2 , sum,na.rm=TRUE) -> RIS[i, ]
      RIS
      i = i+1
      s = s+SumUp
      print(s)
    }
    if (s+SumUp > lr)   {
      print(i)
      apply(tab_9[(s:lr), ], 2 , sum) -> RIS[i, ]
      RIS;
    }
  }

#add zeitgeber time (h) and age (days) by adding the starting timepoint.
  offset <- 86 * 24 + 5
cbind(
```

```
  time = c(seq(offset, offset + length(RIS[,1])/10 - 0.1 ,0.1)),
  RIS
) -> RIS

#One larvae jumped from one container to another. Therefore the data was
removed after that moment.
RIS[(RIS[,1] > (86 + 3.075)*24 + 5),c(5,6)]<-NA

colnames(RIS)[c(2:10)]<-
c("Ju4.1","Ju4.2","Ju4.3","Ju4.4","Ju4.5","Ju4.6","Ju4.7","Ju4.8","Ju4.9")

#save as table
write.table(RIS,"./results/juvenile4_RIS_6min.txt", quote=F, row.names=F)
```

6.1.2 Adults

Distance moved for adult animals was calculated via Excel as distance between two points (Pt and Pt+1) at the timepoint t and t plus one second.

$$\overrightarrow{P_t P_{t+1}} = \sqrt{(x_t - x_{t+1})^2 + (y_t - y_{t+1})^2 + (z_t - z_{t+1})^2}$$

Therefore, the difference was calculated per coordinate (e.g. X_dist_f_pix, see adult_movement.xlsx) and converted into cm by multiplication of using the conversion factor, how many cm are represented per pixel. See Adult_movement.xlsx for exact formulas.

6.2 Statistical analysis of behavioural data using RAIN

6.2.1 Data preparation

```
#load data
larvae1 <- read.csv("./results/larvae1_RIS_6min.txt", sep="")
larvae2 <- read.csv("./results/larvae2_RIS_6min.txt", sep="")
larvae3 <- read.csv("./results/larvae3_RIS_6min.txt", sep="")
larvae4a <- read.csv("./results/larvae4a_RIS_6min.txt", sep="")
larvae4b <- read.csv("./results/larvae4b_RIS_6min.txt", sep="")
larvae5a <- read.csv("./results/larvae5a_RIS_6min.txt", sep="")
larvae5b <- read.csv("./results/larvae5b_RIS_6min.txt", sep="")
larvae7 <- read.csv("./results/larvae7_RIS_6min.txt", sep="")

juvenile1 <- read.csv("./results/juvenile1_RIS_6min.txt", sep = "")
juvenile2 <- read.csv("./results/juvenile2_RIS_6min.txt", sep = "")
juvenile3 <- read.csv("./results/juvenile3_RIS_6min.txt", sep = "")
juvenile4 <- read.csv("./results/juvenile4_RIS_6min.txt", sep = "")

#combine loaded data frames
merge(x = larvae1, y = larvae2, by = "time", all = TRUE) -> data
merge(x = data, y = larvae3, by = "time", all = TRUE) -> data
merge(x = data, y = larvae4a, by = "time", all = TRUE) -> data
merge(x = data, y = larvae4b, by = "time", all = TRUE) -> data
```

```
merge(x = data, y = larvae5a, by = "time", all = TRUE) -> data
merge(x = data, y = larvae5b, by = "time", all = TRUE) -> data
merge(x = data, y = larvae7, by = "time", all = TRUE) -> data
merge(x = data, y = juvenile1, by = "time", all = TRUE) -> data
merge(x = data, y = juvenile2, by = "time", all = TRUE) -> data
merge(x = data, y = juvenile3, by = "time", all = TRUE) -> data
merge(x = data, y = juvenile4, by = "time", all = TRUE) -> data

#delete not used data frames
rm(larvae1,larvae2,larvae3,larvae4a,larvae4b,larvae5a,larvae5b,larvae7,juveni
le1,juvenile2,juvenile3,juvenile4)

#transform pixel to cm data by using the diameter of the tanks:
factor_round <- 1.7
factor_sq_small <- 3
factor_sq_big <- 7.2

data[,c(2:7)]*factor_round -> data[,c(2:7)]         #larvae 1
data[,c(8:13)]*factor_round -> data[,c(8:13)]       #larvae 2
data[,c(14:19)]*factor_round -> data[,c(14:19)]     #larvae 3
data[,c(20:25)]*factor_round -> data[,c(20:25)]     #larvae 4 (a and b)
data[,c(26:37)]*factor_round -> data[,c(26:37)]     #larvae 5 (a and b)
data[,c(38:46)]*factor_sq_small -> data[,c(38:46)]  #larvae 7
data[,c(47:82)]*factor_sq_big -> data[,c(47:82)]    #juvenile 1-4

#calculate mean movement, standard deviation and sample number over all
similar aged animals:
data$mean <- apply(data[,c(2:82)], 1, mean, na.rm = TRUE)
data$sd  <- apply(data[,c(2:82)], 1, sd, na.rm = TRUE)
data$number <-apply(data[,c(2:82)],MARGIN = 1, function(x) sum(!is.na(x)))
data$stderr <- data$sd / sqrt(data$number)

#load and prepare adult movement data
adult_movement_data <- read.csv("./data/adult_movement_data.csv",sep = ";")

  #prepare two additional data frames which include a combination of hour +
min and the ZT-time
  (adult_movement_data$hour*60+adult_movement_data$min)/60 ->
adult_movement_data$h_min_real
  (adult_movement_data$hour*60+adult_movement_data$min)/60 - 6.5 ->
adult_movement_data$ZT
  #sum up data for each analysed timepoint (1min per 30min), necessary for
individual plots
  adult_movement_data %>%
    group_by(ZT, ExperimentID, breeding) %>%
    summarise_at(c("movement_female","movement_male"),sum, na.rm = TRUE) -
>adult_individual
  #switch from wide to long table format
  melt(adult_individual, id.vars = c("ZT","ExperimentID","breeding"),
measure.vars = c("movement_female","movement_male"), value.name =
"movement")->adult_individual
```

```
  #calculate mean and standard deviation (necessary for plot)
  adult_individual %>%
    group_by(ZT, breeding) %>%
    summarise_at(vars(c("movement")),list(~mean(., na.rm = T),~sd(., na.rm =
T))
    ) -> adult.mean


#Data preparation for individual graphs per animal
  #melt data frame into long format
  #separate the animal ID ("variable") into experimentID and number of animal
  melt(data = data, id.vars = c("time")) %>%
  separate(variable, c("ExperimentID","Animal"), remove = FALSE) ->
melted.movement.cpl

## Warning: Expected 2 pieces. Missing pieces filled with `NA` in 31836 rows
## [644680, 644681, 644682, 644683, 644684, 644685, 644686, 644687, 644688,
## 644689, 644690, 644691, 644692, 644693, 644694, 644695, 644696, 644697,
644698,
## 644699, ...].

  #remove all rows which include NA
  melted.movement.cpl[complete.cases(melted.movement.cpl),]-
>melted.movement.cpl

#summarize data per hour for rain analysis
  #adjust downward to get all the full hour
  floor(data$time) -> data$h
  #calculate sum of movement per hour
  data_hourly <- data %>%
  group_by(h) %>%
  summarise_all(list(sum))
  #delete unnecessary columns
  data_hourly <- data_hourly[,-c(2,84:87)]
  colnames(data_hourly)[1] <- "time"
```

6.2.2 Statistcs

The statistical analysis was done using the RAIN package. Each animal was analysed individually and additionally the mean distance travelled of similar aged animal was analysed. The animals were named for the experiment (e.g. La1) and consecutively numbered. 3 Larvae were excluded before analysis because only data for less than one day was available (La7.3, La7.6, La7.9).

```
#The analysis is extremely time consuming. Therefore we decided to sum up per
hour and calculate

#calculate statistics
rain.data <- rbind(
```

```
  rain(subset(data_hourly, !is.na(La1.1))$La1.1, period = 24, deltat =
1,method = "longitudinal"),
  rain(subset(data_hourly, !is.na(La1.2))$La1.2, period = 24, deltat =
1,method = "longitudinal"),
  rain(subset(data_hourly, !is.na(La1.3))$La1.3, period = 24, deltat =
1,method = "longitudinal"),
  rain(subset(data_hourly, !is.na(La1.4))$La1.4, period = 24, deltat =
1,method = "longitudinal"),
  rain(subset(data_hourly, !is.na(La1.5))$La1.5, period = 24, deltat =
1,method = "longitudinal"),
  rain(subset(data_hourly, !is.na(La1.6))$La1.6, period = 24, deltat =
1,method = "longitudinal"),
  rain(subset(data_hourly, !is.na(La2.1))$La2.1, period = 24, deltat =
1,method = "longitudinal"),
  rain(subset(data_hourly, !is.na(La2.2))$La2.2, period = 24, deltat =
1,method = "longitudinal"),
  rain(subset(data_hourly, !is.na(La2.3))$La2.3, period = 24, deltat =
1,method = "longitudinal"),
  rain(subset(data_hourly, !is.na(La2.4))$La2.4, period = 24, deltat =
1,method = "longitudinal"),
  rain(subset(data_hourly, !is.na(La2.5))$La2.5, period = 24, deltat =
1,method = "longitudinal"),
  rain(subset(data_hourly, !is.na(La2.6))$La2.6, period = 24, deltat =
1,method = "longitudinal"),
  rain(subset(data_hourly, !is.na(La3.1))$La3.1, period = 24, deltat =
1,method = "longitudinal"),
  rain(subset(data_hourly, !is.na(La3.2))$La3.2, period = 24, deltat =
1,method = "longitudinal"),
  rain(subset(data_hourly, !is.na(La3.3))$La3.3, period = 24, deltat =
1,method = "longitudinal"),
  rain(subset(data_hourly, !is.na(La3.4))$La3.4, period = 24, deltat =
1,method = "longitudinal"),
  rain(subset(data_hourly, !is.na(La3.5))$La3.5, period = 24, deltat =
1,method = "longitudinal"),
  rain(subset(data_hourly, !is.na(La3.6))$La3.6, period = 24, deltat =
1,method = "longitudinal"),
  rain(subset(data_hourly, !is.na(La4a.1))$La4a.1, period = 24, deltat =
1,method = "longitudinal"),
  rain(subset(data_hourly, !is.na(La4a.2))$La4a.2, period = 24, deltat =
1,method = "longitudinal"),
  rain(subset(data_hourly, !is.na(La4a.3))$La4a.3, period = 24, deltat =
1,method = "longitudinal"),
  rain(subset(data_hourly, !is.na(La4b.1))$La4b.1, period = 24, deltat =
1,method = "longitudinal"),
  rain(subset(data_hourly, !is.na(La4b.2))$La4b.2, period = 24, deltat =
1,method = "longitudinal"),
  rain(subset(data_hourly, !is.na(La4b.3))$La4b.3, period = 24, deltat =
1,method = "longitudinal"),
  rain(subset(data_hourly, !is.na(La5a.1))$La5a.1, period = 24, deltat =
1,method = "longitudinal"),
  rain(subset(data_hourly, !is.na(La5a.2))$La5a.2, period = 24, deltat =
```

```
1,method = "longitudinal"),
  rain(subset(data_hourly, !is.na(La5a.3))$La5a.3, period = 24, deltat =
1,method = "longitudinal"),
  rain(subset(data_hourly, !is.na(La5a.4))$La5a.4, period = 24, deltat =
1,method = "longitudinal"),
  rain(subset(data_hourly, !is.na(La5a.5))$La5a.5, period = 24, deltat =
1,method = "longitudinal"),
  rain(subset(data_hourly, !is.na(La5a.6))$La5a.6, period = 24, deltat =
1,method = "longitudinal"),
  rain(subset(data_hourly, !is.na(La5b.1))$La5b.1, period = 24, deltat =
1,method = "longitudinal"),
  rain(subset(data_hourly, !is.na(La5b.2))$La5b.2, period = 24, deltat =
1,method = "longitudinal"),
  rain(subset(data_hourly, !is.na(La5b.3))$La5b.3, period = 24, deltat =
1,method = "longitudinal"),
  rain(subset(data_hourly, !is.na(La5b.4))$La5b.4, period = 24, deltat =
1,method = "longitudinal"),
  rain(subset(data_hourly, !is.na(La5b.5))$La5b.5, period = 24, deltat =
1,method = "longitudinal"),
  rain(subset(data_hourly, !is.na(La5b.6))$La5b.6, period = 24, deltat =
1,method = "longitudinal"),
  rain(subset(data_hourly, !is.na(La7.1))$La7.1, period = 24, deltat =
1,method = "longitudinal"),
  rain(subset(data_hourly, !is.na(La7.2))$La7.2, period = 24, deltat =
1,method = "longitudinal"),
  #rain(subset(data_hourly, !is.na(La7.3))$La7.3, period = 24, deltat =
1,method = "longitudinal"),
  rain(subset(data_hourly, !is.na(La7.4))$La7.4, period = 24, deltat =
1,method = "longitudinal"),
  rain(subset(data_hourly, !is.na(La7.5))$La7.5, period = 24, deltat =
1,method = "longitudinal"),
  #rain(subset(data_hourly, !is.na(La7.6))$La7.6, period = 24, deltat =
1,method = "longitudinal"),
  rain(subset(data_hourly, !is.na(La7.7))$La7.7, period = 24, deltat =
1,method = "longitudinal"),
  rain(subset(data_hourly, !is.na(La7.8))$La7.8, period = 24, deltat =
1,method = "longitudinal"),
  #rain(subset(data_hourly, !is.na(La7.9))$La7.9, period = 24, deltat =
1,method = "longitudinal"),
  rain(subset(data_hourly, !is.na(Ju1.1))$Ju1.1, period = 24, deltat =
1,method = "longitudinal"),
  rain(subset(data_hourly, !is.na(Ju1.2))$Ju1.2, period = 24, deltat =
1,method = "longitudinal"),
  rain(subset(data_hourly, !is.na(Ju1.3))$Ju1.3, period = 24, deltat =
1,method = "longitudinal"),
  rain(subset(data_hourly, !is.na(Ju1.4))$Ju1.4, period = 24, deltat =
1,method = "longitudinal"),
  rain(subset(data_hourly, !is.na(Ju1.5))$Ju1.5, period = 24, deltat =
1,method = "longitudinal"),
  rain(subset(data_hourly, !is.na(Ju1.6))$Ju1.6, period = 24, deltat =
1,method = "longitudinal"),
```

```
  rain(subset(data_hourly, !is.na(Ju1.7))$Ju1.7, period = 24, deltat =
1,method = "longitudinal"),
  rain(subset(data_hourly, !is.na(Ju1.8))$Ju1.8, period = 24, deltat =
1,method = "longitudinal"),
  rain(subset(data_hourly, !is.na(Ju1.9))$Ju1.9, period = 24, deltat =
1,method = "longitudinal"),
  rain(subset(data_hourly, !is.na(Ju2.1))$Ju2.1, period = 24, deltat =
1,method = "longitudinal"),
  rain(subset(data_hourly, !is.na(Ju2.2))$Ju2.2, period = 24, deltat =
1,method = "longitudinal"),
  rain(subset(data_hourly, !is.na(Ju2.3))$Ju2.3, period = 24, deltat =
1,method = "longitudinal"),
  rain(subset(data_hourly, !is.na(Ju2.4))$Ju2.4, period = 24, deltat =
1,method = "longitudinal"),
  rain(subset(data_hourly, !is.na(Ju2.5))$Ju2.5, period = 24, deltat =
1,method = "longitudinal"),
  rain(subset(data_hourly, !is.na(Ju2.6))$Ju2.6, period = 24, deltat =
1,method = "longitudinal"),
  rain(subset(data_hourly, !is.na(Ju2.7))$Ju2.7, period = 24, deltat =
1,method = "longitudinal"),
  rain(subset(data_hourly, !is.na(Ju2.8))$Ju2.8, period = 24, deltat =
1,method = "longitudinal"),
  rain(subset(data_hourly, !is.na(Ju2.9))$Ju2.9, period = 24, deltat =
1,method = "longitudinal"),
  rain(subset(data_hourly, !is.na(Ju3.1))$Ju3.1, period = 24, deltat =
1,method = "longitudinal"),
  rain(subset(data_hourly, !is.na(Ju3.2))$Ju3.2, period = 24, deltat =
1,method = "longitudinal"),
  rain(subset(data_hourly, !is.na(Ju3.3))$Ju3.3, period = 24, deltat =
1,method = "longitudinal"),
  rain(subset(data_hourly, !is.na(Ju3.4))$Ju3.4, period = 24, deltat =
1,method = "longitudinal"),
  rain(subset(data_hourly, !is.na(Ju3.5))$Ju3.5, period = 24, deltat =
1,method = "longitudinal"),
  rain(subset(data_hourly, !is.na(Ju3.6))$Ju3.6, period = 24, deltat =
1,method = "longitudinal"),
  rain(subset(data_hourly, !is.na(Ju3.7))$Ju3.7, period = 24, deltat =
1,method = "longitudinal"),
  rain(subset(data_hourly, !is.na(Ju3.8))$Ju3.8, period = 24, deltat =
1,method = "longitudinal"),
  rain(subset(data_hourly, !is.na(Ju3.9))$Ju3.9, period = 24, deltat =
1,method = "longitudinal"),
  rain(subset(data_hourly, !is.na(Ju4.1))$Ju4.1, period = 24, deltat =
1,method = "longitudinal"),
  rain(subset(data_hourly, !is.na(Ju4.2))$Ju4.2, period = 24, deltat =
1,method = "longitudinal"),
  rain(subset(data_hourly, !is.na(Ju4.3))$Ju4.3, period = 24, deltat =
1,method = "longitudinal"),
  rain(subset(data_hourly, !is.na(Ju4.4))$Ju4.4, period = 24, deltat =
1,method = "longitudinal"),
  rain(subset(data_hourly, !is.na(Ju4.5))$Ju4.5, period = 24, deltat =
```

```
1,method = "longitudinal"),
  rain(subset(data_hourly, !is.na(Ju4.6))$Ju4.6, period = 24, deltat =
1,method = "longitudinal"),
  rain(subset(data_hourly, !is.na(Ju4.7))$Ju4.7, period = 24, deltat =
1,method = "longitudinal"),
  rain(subset(data_hourly, !is.na(Ju4.8))$Ju4.8, period = 24, deltat =
1,method = "longitudinal"),
  rain(subset(data_hourly, !is.na(Ju4.9))$Ju4.9, period = 24, deltat =
1,method = "longitudinal")
  )

write.table(rain.data, "./results/rain.data.raw.txt")

#proportion of significantly rhythmic animals
length(rain.data[rain.data$pVal < 0.05, 1])/length(rain.data[, 1])

## [1] 0.9615385

#prepare data frame
start_time <- rbind(
      subset(data,!is.na(La1.1))$time[1],
      subset(data,!is.na(La1.2))$time[1],
      subset(data,!is.na(La1.3))$time[1],
      subset(data,!is.na(La1.4))$time[1],
      subset(data,!is.na(La1.5))$time[1],
      subset(data,!is.na(La1.6))$time[1],
      subset(data,!is.na(La2.1))$time[1],
      subset(data,!is.na(La2.2))$time[1],
      subset(data,!is.na(La2.3))$time[1],
      subset(data,!is.na(La2.4))$time[1],
      subset(data,!is.na(La2.5))$time[1],
      subset(data,!is.na(La2.6))$time[1],
      subset(data,!is.na(La3.1))$time[1],
      subset(data,!is.na(La3.2))$time[1],
      subset(data,!is.na(La3.3))$time[1],
      subset(data,!is.na(La3.4))$time[1],
      subset(data,!is.na(La3.5))$time[1],
      subset(data,!is.na(La3.6))$time[1],
      subset(data,!is.na(La4a.1))$time[1],
      subset(data,!is.na(La4a.2))$time[1],
      subset(data,!is.na(La4a.3))$time[1],
      subset(data,!is.na(La4b.1))$time[1],
      subset(data,!is.na(La4b.2))$time[1],
      subset(data,!is.na(La4b.3))$time[1],
      subset(data,!is.na(La5a.1))$time[1],
      subset(data,!is.na(La5a.2))$time[1],
      subset(data,!is.na(La5a.3))$time[1],
      subset(data,!is.na(La5a.4))$time[1],
      subset(data,!is.na(La5a.5))$time[1],
      subset(data,!is.na(La5a.6))$time[1],
      subset(data,!is.na(La5b.1))$time[1],
      subset(data,!is.na(La5b.2))$time[1],
```

```
      subset(data,!is.na(La5b.3))$time[1],
      subset(data,!is.na(La5b.4))$time[1],
      subset(data,!is.na(La5b.5))$time[1],
      subset(data,!is.na(La5b.6))$time[1],
      subset(data,!is.na(La7.1))$time[1],
      subset(data,!is.na(La7.2))$time[1],
#      subset(data,!is.na(La7.3))$time[1],   #was removed (no data)
      subset(data,!is.na(La7.4))$time[1],
      subset(data,!is.na(La7.5))$time[1],
#      subset(data,!is.na(La7.6))$time[1],   #was removed (no data)
      subset(data,!is.na(La7.7))$time[1],
      subset(data,!is.na(La7.8))$time[1],
#      subset(data,!is.na(La7.9))$time[1],   #was removed (no data)
      subset(data,!is.na(Ju1.1))$time[1],
      subset(data,!is.na(Ju1.2))$time[1],
      subset(data,!is.na(Ju1.3))$time[1],
      subset(data,!is.na(Ju1.4))$time[1],
      subset(data,!is.na(Ju1.5))$time[1],
      subset(data,!is.na(Ju1.6))$time[1],
      subset(data,!is.na(Ju1.7))$time[1],
      subset(data,!is.na(Ju1.8))$time[1],
      subset(data,!is.na(Ju1.9))$time[1],
      subset(data,!is.na(Ju2.1))$time[1],
      subset(data,!is.na(Ju2.2))$time[1],
      subset(data,!is.na(Ju2.3))$time[1],
      subset(data,!is.na(Ju2.4))$time[1],
      subset(data,!is.na(Ju2.5))$time[1],
      subset(data,!is.na(Ju2.6))$time[1],
      subset(data,!is.na(Ju2.7))$time[1],
      subset(data,!is.na(Ju2.8))$time[1],
      subset(data,!is.na(Ju2.9))$time[1],
      subset(data,!is.na(Ju3.1))$time[1],
      subset(data,!is.na(Ju3.2))$time[1],
      subset(data,!is.na(Ju3.3))$time[1],
      subset(data,!is.na(Ju3.4))$time[1],
      subset(data,!is.na(Ju3.5))$time[1],
      subset(data,!is.na(Ju3.6))$time[1],
      subset(data,!is.na(Ju3.7))$time[1],
      subset(data,!is.na(Ju3.8))$time[1],
      subset(data,!is.na(Ju3.9))$time[1],
      subset(data,!is.na(Ju4.1))$time[1],
      subset(data,!is.na(Ju4.2))$time[1],
      subset(data,!is.na(Ju4.3))$time[1],
      subset(data,!is.na(Ju4.4))$time[1],
      subset(data,!is.na(Ju4.5))$time[1],
      subset(data,!is.na(Ju4.6))$time[1],
      subset(data,!is.na(Ju4.7))$time[1],
      subset(data,!is.na(Ju4.8))$time[1],
      subset(data,!is.na(Ju4.9))$time[1]
)
```

```
#combine the statistics with the start time and label of animals
rain.data <- cbind(rain.data, start_time,
      animal =
c("La1.1","La1.2","La1.3","La1.4","La1.5","La1.6","La2.1","La2.2","La2.3","La
2.4","La2.5","La2.6","La3.1","La3.2","La3.3","La3.4","La3.5","La3.6","La4a.1"
,"La4a.2","La4a.3","La4b.1","La4b.2","La4b.3","La5a.1","La5a.2","La5a.3","La5
a.4","La5a.5","La5a.6","La5b.1","La5b.2","La5b.3","La5b.4","La5b.5","La5b.6",
"La7.1","La7.2","La7.4","La7.5","La7.7","La7.8","Ju1.1","Ju1.2","Ju1.3","Ju1.
4","Ju1.5","Ju1.6","Ju1.7","Ju1.8","Ju1.9","Ju2.1","Ju2.2","Ju2.3","Ju2.4","J
u2.5","Ju2.6","Ju2.7","Ju2.8","Ju2.9","Ju3.1","Ju3.2","Ju3.3","Ju3.4","Ju3.5"
,"Ju3.6","Ju3.7","Ju3.8","Ju3.9","Ju4.1","Ju4.2","Ju4.3","Ju4.4","Ju4.5","Ju4
.6","Ju4.7","Ju4.8","Ju4.9")
      )

#exclude unrhythmic animals
rain.data <- rain.data[rain.data$pVal < 0.05, ]

#Experiments started at different timepoints, so ZT has to be added to the
phase calculated via RAIN. Therefore the time has to be added to the data
frame and we prepare the data frame:

#Distill experiment ID from animal
rain.data <- rain.data %>% separate(animal,c("experiment"), remove = FALSE,
extra = "drop")

#make a new data frame which include the age in days, when the animals were
tested
experimental_start <- data.frame(
  "experiment" =
c("La1","La2","La3","La4a","La4b","La5a","La5b","La7","Ju1","Ju2","Ju3","Ju4"
),
  "start_age" = c(7, 15, 12, 11, 8, 14, 11, 10, 98, 100, 52, 86)
)

#combine the data frames
rain.data <- merge(rain.data, experimental_start, by.x = "experiment", by.y =
"experiment")

#calculate the ZT-start by subtraction the start time of the experiment and
the age converted into hours
rain.data$start_ZT <- rain.data$start_time - rain.data$start_age*24

#calculate corrected phase
rain.data$corrected_phase <- rain.data$phase + rain.data$start_ZT

#ZT cannot be larger than 24h - so we correct for it.
rain.data[rain.data$corrected_phase > 24,]$corrected_phase <-
rain.data[rain.data$corrected_phase > 24,]$corrected_phase - 24

#add the developmental level (larvae or juveniles)
```

```
rain.data$devel <- substr(rain.data$experiment,1,2)

#replace La and Ju for better readability
rain.data$devel <- gsub("Ju", "juvenile",rain.data$devel)
rain.data$devel <- gsub("La", "larvae",rain.data$devel)

#display the data frame, devel refers to developmental level (larvae,
juvenile, adult)
rain.data

##    experiment         pVal phase peak.shape period start_time animal
start_age
## 1         Ju1 2.779217e-05    17         16     24     2361.0  Ju1.1
98
## 2         Ju1 5.165747e-17    22         10     24     2361.0  Ju1.2
98
## 3         Ju1 5.407938e-06    16         15     24     2361.0  Ju1.3
98
## 4         Ju1 1.028819e-09     9         15     24     2361.0  Ju1.4
98
## 5         Ju1 4.132000e-13    21         17     24     2361.0  Ju1.5
98
## 6         Ju1 1.836715e-06    21         17     24     2361.0  Ju1.6
98
## 7         Ju1 2.553277e-36    21         17     24     2361.0  Ju1.7
98
## 8         Ju1 1.977023e-02    21         17     24     2361.0  Ju1.8
98
## 9         Ju1 9.190455e-15    12         11     24     2361.0  Ju1.9
98
## 10        Ju2 5.087044e-22     6         10     24     2409.0  Ju2.1
100
## 11        Ju2 4.972522e-02     9          7     24     2409.0  Ju2.2
100
## 12        Ju2 4.052121e-08    23         16     24     2409.0  Ju2.3
100
## 13        Ju2 8.630393e-17    23         17     24     2409.0  Ju2.4
100
## 14        Ju2 1.098300e-24    23          9     24     2409.0  Ju2.6
100
## 15        Ju2 9.023979e-14    22         16     24     2409.0  Ju2.8
100
## 16        Ju2 9.539811e-19     5         11     24     2409.0  Ju2.9
100
## 17        Ju3 9.799979e-08     3         13     24     1257.0  Ju3.1
52
## 18        Ju3 3.201043e-11     5          8     24     1257.0  Ju3.2
52
## 19        Ju3 8.495998e-07     1          7     24     1257.0  Ju3.3
52
## 20        Ju3 3.717096e-04     5         17     24     1257.0  Ju3.4
```

```
52
## 21         Ju3 1.723601e-21    21          15      24      1257.0  Ju3.5
52
## 22         Ju3 1.464589e-02    23          17      24      1257.0  Ju3.6
52
## 23         Ju3 2.708211e-04    10          17      24      1257.0  Ju3.7
52
## 24         Ju3 5.482518e-04    18          16      24      1257.0  Ju3.8
52
## 25         Ju3 1.473042e-02     4          16      24      1257.0  Ju3.9
52
## 26         Ju4 3.049646e-19    16          10      24      2069.0  Ju4.1
86
## 27         Ju4 4.102800e-18    23          15      24      2069.0  Ju4.2
86
## 28         Ju4 2.830534e-16    13          17      24      2069.0  Ju4.4
86
## 29         Ju4 1.997169e-02     2           7      24      2069.0  Ju4.5
86
## 30         Ju4 6.845130e-30    14          10      24      2069.0  Ju4.6
86
## 31         Ju4 4.600745e-15     9          17      24      2069.0  Ju4.7
86
## 32         Ju4 1.329364e-05    17          17      24      2069.0  Ju4.8
86
## 33         Ju4 1.895617e-26    15          12      24      2069.0  Ju4.9
86
## 34         La1 1.032696e-06     4          17      24       180.0  La1.1
7
## 35         La1 1.153286e-11     6          12      24       180.0  La1.2
7
## 36         La1 6.127098e-06     1          17      24       180.0  La1.3
7
## 37         La1 1.157353e-05     7           8      24       180.0  La1.4
7
## 38         La1 4.341683e-12     5          11      24       180.0  La1.5
7
## 39         La1 1.272998e-07     8          13      24       180.0  La1.6
7
## 40         La2 1.236181e-32     7          11      24       371.0  La2.1
15
## 41         La2 8.014852e-22     6          12      24       371.0  La2.2
15
## 42         La2 7.561642e-31     2          16      24       371.0  La2.3
15
## 43         La2 2.131104e-09     2          15      24       371.0  La2.4
15
## 44         La2 9.614748e-30     6          12      24       371.0  La2.5
15
## 45         La2 3.006486e-26     9          17      24       389.3  La2.6
15
```

```
## 46         La3 9.482422e-04    11           17      24       290.0  La3.1
12
## 47         La3 4.290938e-26    16            9      24       290.0  La3.2
12
## 48         La3 1.271032e-21    11           17      24       290.0  La3.3
12
## 49         La3 1.563812e-47    12           17      24       290.0  La3.4
12
## 50         La3 5.058246e-31    16           11      24       290.0  La3.5
12
## 51         La3 6.590367e-16    11           17      24       290.0  La3.6
12
## 52        La4a 2.082534e-26     6           17      24       271.0 La4a.1
11
## 53        La4a 7.234247e-22     7           14      24       271.0 La4a.2
11
## 54        La4a 5.361783e-21     9           13      24       291.7 La4a.3
11
## 55        La4b 1.561720e-07     6           15      24       199.0 La4b.1
8
## 56        La4b 3.638779e-02     6           15      24       199.0 La4b.2
8
## 57        La4b 3.223605e-13     6           15      24       199.0 La4b.3
8
## 58        La5a 1.092709e-13     7           17      24       342.0 La5a.1
14
## 59        La5a 6.722303e-04    15            9      24       342.0 La5a.2
14
## 60        La5a 1.988161e-07    14            9      24       342.0 La5a.3
14
## 61        La5a 4.113971e-25     8           17      24       342.0 La5a.4
14
## 62        La5a 6.105571e-18     7           17      24       342.0 La5a.5
14
## 63        La5a 7.705110e-13     9           17      24       342.0 La5a.6
14
## 64        La5b 5.528313e-27    12           16      24       270.0 La5b.1
11
## 65        La5b 2.776923e-22     9           17      24       270.0 La5b.2
11
## 66        La5b 2.024615e-23     9           17      24       270.0 La5b.3
11
## 67        La5b 1.477588e-47    10           16      24       270.0 La5b.4
11
## 68        La5b 1.984207e-29    12           14      24       270.0 La5b.5
11
## 69        La5b 1.804695e-18    11           15      24       270.0 La5b.6
11
## 70         La7 8.093459e-26     8           17      24       246.0  La7.1
10
## 71         La7 5.251799e-10     8           17      24       246.0  La7.2
```

```
10
## 72        La7 5.915935e-15     9         17      24       246.0  La7.4
10
## 73        La7 7.843705e-03    13         11      24       246.0  La7.5
10
## 74        La7 5.122455e-18     7         17      24       246.0  La7.7
10
## 75        La7 6.741957e-21     8         16      24       246.0  La7.8
10
##    start_ZT corrected_phase    devel
## 1       9.0             2.0 juvenile
## 2       9.0             7.0 juvenile
## 3       9.0             1.0 juvenile
## 4       9.0            18.0 juvenile
## 5       9.0             6.0 juvenile
## 6       9.0             6.0 juvenile
## 7       9.0             6.0 juvenile
## 8       9.0             6.0 juvenile
## 9       9.0            21.0 juvenile
## 10      9.0            15.0 juvenile
## 11      9.0            18.0 juvenile
## 12      9.0             8.0 juvenile
## 13      9.0             8.0 juvenile
## 14      9.0             8.0 juvenile
## 15      9.0             7.0 juvenile
## 16      9.0            14.0 juvenile
## 17      9.0            12.0 juvenile
## 18      9.0            14.0 juvenile
## 19      9.0            10.0 juvenile
## 20      9.0            14.0 juvenile
## 21      9.0             6.0 juvenile
## 22      9.0             8.0 juvenile
## 23      9.0            19.0 juvenile
## 24      9.0             3.0 juvenile
## 25      9.0            13.0 juvenile
## 26      5.0            21.0 juvenile
## 27      5.0             4.0 juvenile
## 28      5.0            18.0 juvenile
## 29      5.0             7.0 juvenile
## 30      5.0            19.0 juvenile
## 31      5.0            14.0 juvenile
## 32      5.0            22.0 juvenile
## 33      5.0            20.0 juvenile
## 34     12.0            16.0   larvae
## 35     12.0            18.0   larvae
## 36     12.0            13.0   larvae
## 37     12.0            19.0   larvae
## 38     12.0            17.0   larvae
## 39     12.0            20.0   larvae
## 40     11.0            18.0   larvae
## 41     11.0            17.0   larvae
```

```
## 42     11.0            13.0   larvae
## 43     11.0            13.0   larvae
## 44     11.0            17.0   larvae
## 45     29.3            14.3   larvae
## 46      2.0            13.0   larvae
## 47      2.0            18.0   larvae
## 48      2.0            13.0   larvae
## 49      2.0            14.0   larvae
## 50      2.0            18.0   larvae
## 51      2.0            13.0   larvae
## 52      7.0            13.0   larvae
## 53      7.0            14.0   larvae
## 54     27.7            12.7   larvae
## 55      7.0            13.0   larvae
## 56      7.0            13.0   larvae
## 57      7.0            13.0   larvae
## 58      6.0            13.0   larvae
## 59      6.0            21.0   larvae
## 60      6.0            20.0   larvae
## 61      6.0            14.0   larvae
## 62      6.0            13.0   larvae
## 63      6.0            15.0   larvae
## 64      6.0            18.0   larvae
## 65      6.0            15.0   larvae
## 66      6.0            15.0   larvae
## 67      6.0            16.0   larvae
## 68      6.0            18.0   larvae
## 69      6.0            17.0   larvae
## 70      6.0            14.0   larvae
## 71      6.0            14.0   larvae
## 72      6.0            15.0   larvae
## 73      6.0            19.0   larvae
## 74      6.0            13.0   larvae
## 75      6.0            14.0   larvae
```

```
write.table(rain.data, "./results/rain.data.txt")

#calculate statistics for adults
rbind(
    rain(as.data.frame(tapply(subset(adult_movement_data, ExperimentID ==
1)$movement_male, subset(adult_movement_data, ExperimentID == 1)$hour,mean,
na.rm = TRUE)), period = 24, deltat = 1),
    rain(as.data.frame(tapply(subset(adult_movement_data, ExperimentID ==
4)$movement_male, subset(adult_movement_data, ExperimentID == 4)$hour,mean,
na.rm = TRUE)), period = 24, deltat = 1),
    rain(as.data.frame(tapply(subset(adult_movement_data, ExperimentID ==
5)$movement_male, subset(adult_movement_data, ExperimentID == 5)$hour,mean,
na.rm = TRUE)), period = 24, deltat = 1),
    rain(as.data.frame(tapply(subset(adult_movement_data, ExperimentID ==
6)$movement_male, subset(adult_movement_data, ExperimentID == 6)$hour,mean,
na.rm = TRUE)), period = 24, deltat = 1),
```

```
    rain(as.data.frame(tapply(subset(adult_movement_data, ExperimentID ==
1)$movement_female, subset(adult_movement_data, ExperimentID == 1)$hour,mean,
na.rm = TRUE)), period = 24, deltat = 1),
    rain(as.data.frame(tapply(subset(adult_movement_data, ExperimentID ==
4)$movement_female, subset(adult_movement_data, ExperimentID == 4)$hour,mean,
na.rm = TRUE)), period = 24, deltat = 1),
    rain(as.data.frame(tapply(subset(adult_movement_data, ExperimentID ==
5)$movement_female, subset(adult_movement_data, ExperimentID == 5)$hour,mean,
na.rm = TRUE)), period = 24, deltat = 1),
    rain(as.data.frame(tapply(subset(adult_movement_data, ExperimentID ==
6)$movement_female, subset(adult_movement_data, ExperimentID == 6)$hour,mean,
na.rm = TRUE)), period = 24, deltat = 1)
)-> result.adult.rain

cbind(
  result.adult.rain,
  animal =
c("adult1","adult2","adult2","adult1","adult3","adult4","adult4","adult3"),
  sex = c("male","male","male","male","female","female","female","female"),
  breedingstatus =
c("without_eggs","without_eggs","eggs","eggs","without_eggs","without_eggs","
eggs","eggs")
)->result.adult.rain

rownames(result.adult.rain)<- c()

result.adult.rain

##           pVal phase peak.shape period animal    sex breedingstatus
## 1 2.838400e-06     4         17     24 adult1   male   without_eggs
## 2 2.789742e-06    11          9     24 adult2   male   without_eggs
## 3 1.216629e-05    10          8     24 adult2   male           eggs
## 4 7.582963e-04     3         15     24 adult1   male           eggs
## 5 1.053577e-11    10         13     24 adult3 female   without_eggs
## 6 9.621190e-06     5         14     24 adult4 female   without_eggs
## 7 3.936250e-08     5         17     24 adult4 female           eggs
## 8 1.896783e-05     7         17     24 adult3 female           eggs

write.table(result.adult.rain, "./results/results.adult.rain.txt")

#calculate summary how many animals are nocturnal (ZT larger than 12) or
diurnal (ZT less or equal 12).
cbind(
  rbind(
  #larvae
  length(subset(rain.data, devel == "larvae" & corrected_phase >= 0 &
corrected_phase <= 11)$animal),
  length(subset(rain.data, devel == "larvae" & corrected_phase >= 12 &
corrected_phase <= 23)$animal),
```

```
  #young juveniles (start age 52 dph), experiment: Juvenile 3
  length(subset(rain.data, start_age == 52 & corrected_phase >= 0 &
corrected_phase <= 11)$animal),
  length(subset(rain.data, start_age == 52 & corrected_phase >= 12 &
corrected_phase <= 23)$animal),

  #intermediate juvenile (start age 86 dph), experiment: Juvenile 4
  length(subset(rain.data, start_age == 86 & corrected_phase >= 0 &
corrected_phase <= 11)$animal),
  length(subset(rain.data, start_age == 86 & corrected_phase >= 12 &
corrected_phase <= 23)$animal),

  #old juvenile (start age 97 and 100 dph), experiments: Juvenile 1 + 2
  length(subset(rain.data, devel == "juvenile" & start_age > 97 &
corrected_phase >= 0 & corrected_phase <= 11)$animal),
  length(subset(rain.data, devel == "juvenile" & start_age > 97 &
corrected_phase >= 12 & corrected_phase <= 23)$animal),

  #adults
  length(subset(result.adult.rain, phase >= 0 & phase <= 11)$animal),
  length(subset(result.adult.rain, phase >= 12 & phase <= 23)$animal)
  ),

  rbind(
  #larvae
  round(length(subset(rain.data, devel == "larvae" & corrected_phase >= 0 &
corrected_phase <= 11)$animal)/length(subset(rain.data, devel ==
"larvae")$animal),4),
  round(length(subset(rain.data, devel == "larvae" & corrected_phase >= 12 &
corrected_phase <= 23)$animal)/length(subset(rain.data, devel ==
"larvae")$animal),4),

  #young juveniles (start age 52 dph), experiment: Juvenile 3
  round(length(subset(rain.data, start_age == 52 & corrected_phase >= 0 &
corrected_phase <= 11)$animal)/ length(subset(rain.data, start_age ==
52)$animal),4),
  round(length(subset(rain.data, start_age == 52 & corrected_phase >= 12 &
corrected_phase <= 23)$animal)/ length(subset(rain.data, start_age ==
52)$animal),4),

  #intermediate juvenile (start age 86 dph), experiment: Juvenile 4
  round(length(subset(rain.data, start_age == 86 & corrected_phase >= 0 &
corrected_phase <= 11)$animal)/length(subset(rain.data, start_age ==
86)$animal),4),
  round(length(subset(rain.data, start_age == 86 & corrected_phase >= 12 &
corrected_phase <= 23)$animal)/length(subset(rain.data, start_age ==
86)$animal),4),

  #old juvenile (start age 97 and 100 dph), experiments: Juvenile 1 + 2
  round(length(subset(rain.data, devel == "juvenile" & start_age > 97 &
```

```
corrected_phase >= 0 & corrected_phase <=
11)$animal)/length(subset(rain.data, devel == "juvenile" & start_age >
97)$animal),4),
  round(length(subset(rain.data, devel == "juvenile" & start_age > 97 &
corrected_phase >= 12 & corrected_phase <=
23)$animal)/length(subset(rain.data, devel == "juvenile" & start_age >
97)$animal),4),

  #adults
  round(length(subset(result.adult.rain, phase >= 0 & phase <=
11)$animal)/length(result.adult.rain$animal),4),
  round(length(subset(result.adult.rain, phase >= 12 & phase <=
23)$animal)/length(result.adult.rain$animal),4)

  ))-> overview

#add labels to the data frame
cbind(sample = c("larvae","larvae","juvenile 1","juvenile 1","juvenile 2",
"juvenile 2", "juvenile 3","juvenile 3", "adult", "adult"),
      age = c("7-24 \n larvae","7-24 \n larvae","52-57 \n juveniles", "52-57
\n juveniles", "86-91 \n juveniles", "86-91 \n juveniles", "98-106 \n
juveniles","98-106 \n juveniles", ">365 \n adults",">365 \n adults"),
      daytime =
c("day","night","day","night","day","night","day","night","day","night"),
      proportion = as.data.frame(overview)) -> overview

colnames(overview)[c(4,5)] <- c("number","proportion")

#define levels
overview$sample <- factor(overview$sample, levels = c("larvae","juvenile
1","juvenile 2","juvenile 3","adult"))
overview$age <- factor(overview$age, levels = unique(overview$age))
```

6.2.2.1 Calculation of diurnality index

```
diurnality_index <- data.frame(
        sample = as.character(unique(overview$sample)),
        age = as.character(unique(overview$age)),
        index = c((overview[1,4] - overview[2,4])/(overview[1,4] +
overview[2,4]),
                (overview[3,4] - overview[4,4])/(overview[3,4] +
overview[4,4]),
                (overview[5,4] - overview[6,4])/(overview[5,4] +
overview[6,4]),
                (overview[7,4] - overview[8,4])/(overview[7,4] +
overview[8,4]),
                (overview[9,4] - overview[10,4])/(overview[9,4] +
overview[10,4])
        ))

#define levels
diurnality_index$sample <- factor(diurnality_index$sample, levels =
```

```
c("larvae","juvenile 1","juvenile 2","juvenile 3","adult"))
diurnality_index$age <- factor(diurnality_index$age, levels =
unique(diurnality_index$age))

diurnality_index
```

```
##       sample                  age      index
## 1      larvae      7-24 \n larvae -1.0000000
## 2 juvenile 1  52-57 \n juveniles -0.1111111
## 3 juvenile 2  86-91 \n juveniles -0.5000000
## 4 juvenile 3 98-106 \n juveniles  0.3750000
## 5       adult       >365 \n adults  1.0000000
```

6.2.3 Graphics

6.2.3.1 Figure 1

The graphic is included as figure 1 in the manuscript.

```
#define on and offset of light:
light <- data.frame(
  lightoff =seq(12,120*24,24),
  lighton = seq(24,120.5*24,24)
)

#prepare general plot
q<- ggplot(data, aes(x = time/24, y = mean/100)) + theme_bw()

#plot larval movement data
larv_plot1 <- q +
  annotate('rect', xmin=light$lighton/24,xmax=light$lightoff/24,ymin=-
Inf,ymax=Inf,fill = "steelblue4", alpha = 0.5)+
  geom_point(size = 1)+
  geom_errorbar(aes(ymin=mean/100-stderr/100,
ymax=mean/100+stderr/100,x=time/24), size=.01,width=0.03)+
  scale_x_continuous(limits = c(10.991,13.001),breaks =
seq(11,13,0.125),labels = c((seq(11,12,0.125)-11)*24,(seq(12.125,13,0.125)-
12)*24))+
  scale_y_continuous(limits = c(-0,0.5))+
  ylab("")+
  xlab(NULL)+
  theme(plot.margin = unit(c(1,0.1,0.1,1.5), "cm"))

#plot juvenile movement data
juv_plot1 <-
  q+
  annotate('rect', xmin=light$lighton/24,xmax=light$lightoff/24,ymin=-
Inf,ymax=Inf,fill = "steelblue4", alpha = 0.5)+
  geom_point(size = 1)+
  geom_errorbar(aes(ymin=mean/100-stderr/100,
ymax=mean/100+stderr/100,x=time/24), size=.01,width=0.02)+
```

```
  scale_y_continuous(limits = c(NA,2.5))+
  scale_x_continuous(limits = c(98.991,101.001),breaks =
seq(99,101,0.125),labels = c((seq(99,100,0.125)-
99)*24,(seq(100.125,101,0.125)-100)*24))+
  xlab(NULL)+
  ylab(NULL)+
  theme(plot.margin = unit(c(1,0.1,0.1,1.98), "cm"))

#plot adult movement data
adult.plot <-
  ggplot(adult.mean)+
  geom_point(aes(x= ZT, y = mean/100))+
  geom_errorbar(aes(ymin=mean/100-(sd/sqrt(4))/100,
ymax=mean/100+sd/100/sqrt(4),x=ZT), size=.01,width=1)+
  annotate("rect", xmin=-0.5,xmax=0, ymin=-Inf,ymax=Inf,fill =
"steelblue4",alpha = 0.5)+
  annotate("rect", xmin=0,xmax=0.25, ymin=-Inf,ymax=Inf,fill =
"steelblue4",alpha = 0.3)+
  annotate("rect", xmin=13.5,xmax=14, ymin=-Inf,ymax=Inf, fill =
"steelblue4",alpha = 0.3)+
  annotate("rect", xmin=14,xmax=24, ymin=-Inf,ymax=Inf, fill =
"steelblue4",alpha = 0.5)+
  facet_wrap(. ~ breeding, ncol = 2)+
  scale_x_continuous(limits=c(NA,24.1),breaks = c(seq(0,24,3)))+
  xlab("zeitgeber time (h)")+
  ylab(NULL)+
  theme_bw()+
  theme(plot.margin = unit(c(1,0.1,0.1,2.25), "cm"))

activityplot<-
plot_grid(larv_plot1,juv_plot1,adult.plot,labels=c("larvae","juveniles","adul
ts"),ncol = 1,label_x = 0, align = "h", axis = "r",  hjust = c(-1.29,-0.92,-
1.3))+
  draw_label("mean distance travelled \n (m/6min)", x=  0.01, y=0.67, vjust=
1.5, angle=90)+
  draw_label("mean distance travelled \n (m/1min)", x=  0.01, y=0.176, vjust=
1.5, angle=90)

## Warning: Removed 118 rows containing missing values (geom_rect).

## Warning: Removed 7476 rows containing missing values (geom_point).

## Warning: Removed 118 rows containing missing values (geom_rect).

## Warning: Removed 7476 rows containing missing values (geom_point).

#library magick is necessary
ggdraw(activityplot)+
  draw_image("./illustration/larvae.png",  x = 0.41, y = 0.4, scale = .18)+
  draw_image("./illustration/juv.png",  x = 0.41, y = 0.07, scale = .18)+
  draw_image("./illustration/adult_turned.png",  x = 0.41, y = -0.30, scale =
.15)
```

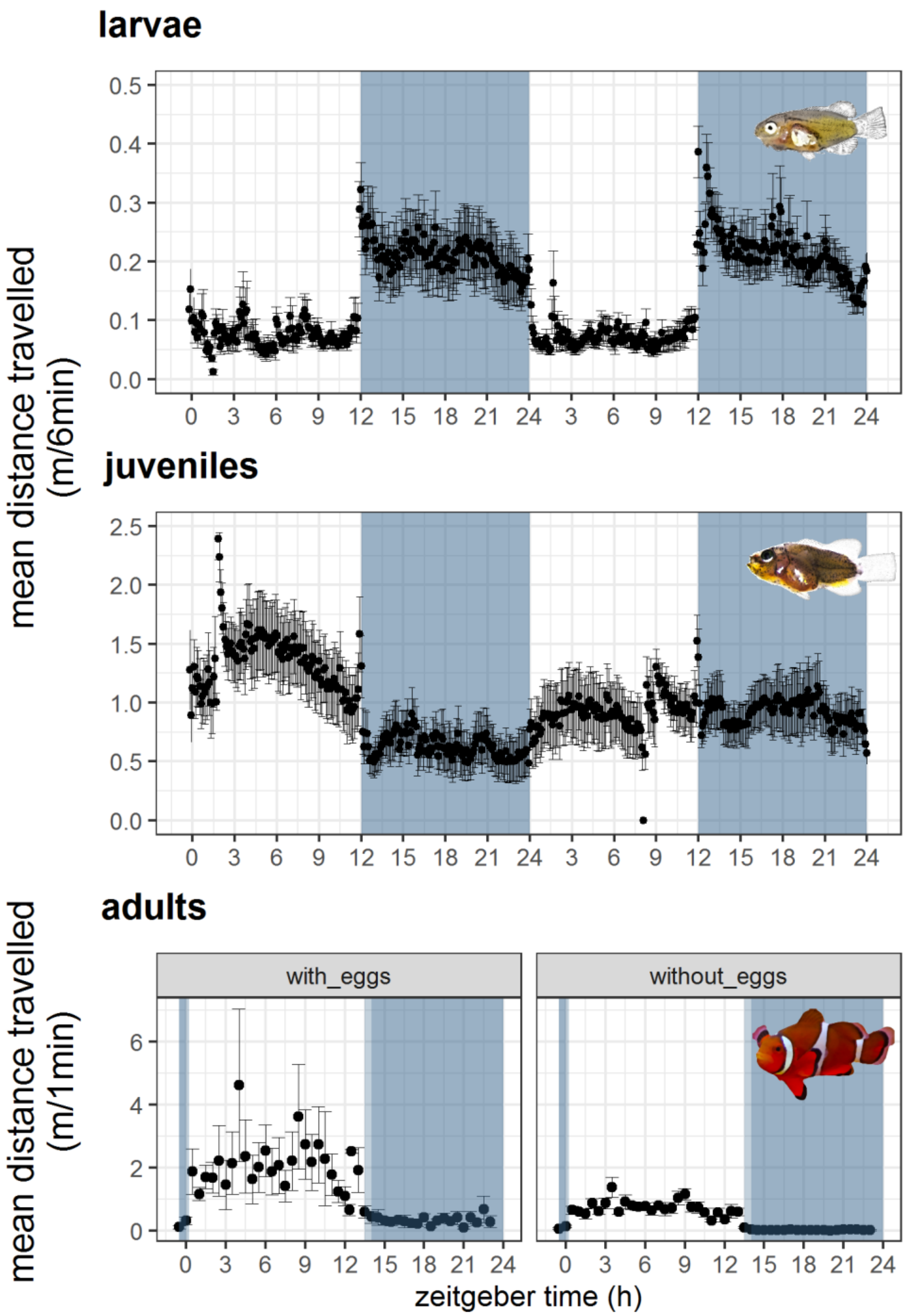


```
ggsave("./results/activity_plot.png",width = 16, height = 17, units = "cm")
ggsave("./results/activity_plot.pdf",width = 16, height = 17, units = "cm")
```

6.2.3.2 Additional: Seperate graph per animal

```
#single graphs

#prepare separated graphs for each tested individual


adult_individual$plot <- paste("ExpID:",
adult_individual$ExperimentID,adult_individual$breeding)
```

```
#Larvae
subset(melted.movement.cpl,
       ExperimentID == "La1" |
         ExperimentID == "La2" |
         ExperimentID == "La3" |
         ExperimentID == "La5a" |
         ExperimentID == "La5b")  -> melted.mov.compl.larv

subset(melted.mov.compl.larv, time > 9*24 & time < 14*24) -
>melted.mov.compl.larv

light <- data.frame(
  lightoff =seq(9*24 + 12,120*24,24),
  lighton = seq(9*24 + 24,120.5*24,24)
)
#only necessary for visualization
x <- 0.32
y <- 0.641

png("./addition/individual_behaviour_a%03d.png", res = 320, width = 22.75,
height = 14, unit="cm") #activate if separate pdf output is necessary
#larvae1
temp <- ggplot(melted.mov.compl.larv, aes(x= time, y = value/100))+
  annotate('rect', xmin=light$lighton,xmax=light$lightoff,ymin=-
Inf,ymax=Inf,fill = "steelblue4", alpha = 0.5)+
  geom_point(size = 0.01)+
  facet_wrap_paginate(ExperimentID ~ Animal, ncol = 3, nrow = 2, page = 1)+
  theme_bw(base_size = 8)+
  xlab("zeitgeber time (h)")+
  ylab("mean distance travelled (m/6min)")+
  ggtitle("larvae")+
  coord_cartesian(xlim=c(9*24,13*24), ylim = c(0,1.5))+
  scale_x_continuous(breaks = seq(9*24,13.1*24,0.25*24),
                     labels = c("0", "6","12 \n 9 dph", "18", "24",
                                "6", "12 \n 10 dph", "18", "24",
                                "6", "12 \n 11 dph", "18", "24",
                                "6", "12 \n 12 dph", "18", "24"))+
  annotate('rect', xmin=11*24, xmax=11.03*24, ymin=1.54, ymax = 1.5, fill =
'black')+
  annotate('rect', xmin=12.97*24, xmax=13*24, ymin=1.54, ymax = 1.5, fill =
'black')+
  annotate('rect', xmin=11*24, xmax=13*24, ymin=1.54, ymax = 1.52, fill =
'black')+
  annotate("label", x = 12*24, y = 1.52, label = "data fig. 1", size = 2,
label.padding = unit(0.1, "lines"))+
  annotate("segment", x = c(0,48), xend = c(24,72), y = -0.115, yend = -
0.115)+
  annotate("segment", x = c(24,72), xend = c(48,96), y = -0.115, yend = -
0.115, color = "grey")
```

```r
plot_grid(temp)+
#first facet
  draw_line(x = c(0.054, 0.1252), y = c(0.05, 0.05),color = "grey")+
  draw_line(x = c(0.1252,0.197), y = c(0.05, 0.05))+
  draw_line(x = c(0.197, 0.268), y = c(0.05, 0.05),color = "grey")+
  draw_line(x = c(0.268, 0.339), y = c(0.05, 0.05))+
#2nd facet
  draw_line(x = c(0.054+x, 0.1252+x), y = c(0.05, 0.05),color = "grey")+
  draw_line(x = c(0.1252+x,0.197+x), y = c(0.05, 0.05))+
  draw_line(x = c(0.197+x, 0.268+x), y = c(0.05, 0.05),color = "grey")+
  draw_line(x = c(0.268+x, 0.339+x), y = c(0.05, 0.05))+
#3rd facet
  draw_line(x = c(0.054+y, 0.1252+y), y = c(0.05, 0.05),color = "grey")+
  draw_line(x = c(0.1252+y,0.197+y), y = c(0.05, 0.05))+
  draw_line(x = c(0.197+y, 0.268+y), y = c(0.05, 0.05),color = "grey")+
  draw_line(x = c(0.268+y, 0.339+y), y = c(0.05, 0.05))

#larvae3
temp <- ggplot(melted.mov.compl.larv, aes(x= time, y = value/100))+
  geom_point(size = 0.01)+
  facet_wrap_paginate(ExperimentID ~ Animal, ncol = 3, nrow = 2, page = 2)+
  annotate('rect', xmin=light$lighton,xmax=light$lightoff,ymin=-
Inf,ymax=Inf,fill = "steelblue4", alpha = 0.5)+
  theme_bw(base_size = 8)+
  xlab("zeitgeber time (h)")+
  ylab("mean distance travelled (m/6min)")+
  coord_cartesian(xlim=c(11*24,15*24), ylim = c(0,1.5))+
  scale_x_continuous(breaks = seq(11*24,15.1*24,0.25*24),
                     labels = c("0", "6","12 \n 11 dph", "18", "24",
                                "6", "12 \n 12 dph", "18", "24",
                                "6", "12 \n 13 dph", "18", "24",
                                "6", "12 \n 14 dph", "18", "24"))+
  annotate('rect', xmin=11*24, xmax=11.03*24, ymin=1.54, ymax = 1.5, fill =
'black')+
  annotate('rect', xmin=12.97*24, xmax=13*24, ymin=1.54, ymax = 1.5, fill =
'black')+
  annotate('rect', xmin=11*24, xmax=13*24, ymin=1.54, ymax = 1.52, fill =
'black')+
  annotate("label", x = 12*24, y = 1.52, label = "data fig. 1", size = 2,
label.padding = unit(0.1, "lines"))+
  annotate("segment", x = c(0,48), xend = c(24,72), y = -0.115, yend = -
0.115)+
  annotate("segment", x = c(24,72), xend = c(48,96), y = -0.115, yend = -
0.115, color = "grey")

plot_grid(temp)+
  #first facet
  draw_line(x = c(0.054, 0.1252), y = c(0.05, 0.05),color = "grey")+
  draw_line(x = c(0.1252,0.197), y = c(0.05, 0.05))+
  draw_line(x = c(0.197, 0.268), y = c(0.05, 0.05),color = "grey")+
  draw_line(x = c(0.268, 0.339), y = c(0.05, 0.05))+
```

```
  #2nd facet
  draw_line(x = c(0.054+x, 0.1252+x), y = c(0.05, 0.05),color = "grey")+
  draw_line(x = c(0.1252+x,0.197+x), y = c(0.05, 0.05))+
  draw_line(x = c(0.197+x, 0.268+x), y = c(0.05, 0.05),color = "grey")+
  draw_line(x = c(0.268+x, 0.339+x), y = c(0.05, 0.05))+
  #3rd facet
  draw_line(x = c(0.054+y, 0.1252+y), y = c(0.05, 0.05),color = "grey")+
  draw_line(x = c(0.1252+y,0.197+y), y = c(0.05, 0.05))+
  draw_line(x = c(0.197+y, 0.268+y), y = c(0.05, 0.05),color = "grey")+
  draw_line(x = c(0.268+y, 0.339+y), y = c(0.05, 0.05))
dev.off()
```

```
## png
##   2
```

```
png("./addition/individual_behaviour_b%03d.png", res = 320, width = 22.75,
height = 8, unit="cm") #activate if separate pdf output is necessary
#larvae4a
temp <- ggplot(subset(melted.movement.cpl, time > 11*24 & time < 15*24 &
ExperimentID == "La4a"), aes(x= time, y = value/100))+
  geom_point(size = 0.01)+
  facet_wrap(ExperimentID ~ Animal, ncol = 3, nrow = 24)+
  annotate('rect', xmin=light$lighton,xmax=light$lightoff,ymin=-
Inf,ymax=Inf,fill = "steelblue4", alpha = 0.5)+
  theme_bw(base_size = 8)+
  xlab("zeitgeber time (h)")+
  ylab("mean distance travelled (m/6min)")+
  coord_cartesian(xlim=c(11*24,15*24))+
  scale_y_continuous(limits = c(0,1.55))+
  scale_x_continuous(breaks = seq(11*24,15.1*24,0.25*24),
                     labels = c("0", "6","12 \n 11 dph", "18", "24",
                                "6", "12 \n 12 dph", "18", "24",
                                "6", "12 \n 13 dph", "18", "24",
                                "6", "12 \n 14 dph", "18", "24"))+
  annotate('rect', xmin=11*24, xmax=11.03*24, ymin=1.54, ymax = 1.5, fill =
'black')+
  annotate('rect', xmin=12.97*24, xmax=13*24, ymin=1.54, ymax = 1.5, fill =
'black')+
  annotate('rect', xmin=11*24, xmax=13*24, ymin=1.54, ymax = 1.52, fill =
'black')+
  annotate("label", x = 12*24, y = 1.52, label = "data fig. 1", size = 2,
label.padding = unit(0.1, "lines"))+
  annotate("segment", x = c(0,48), xend = c(24,72), y = -0.115, yend = -
0.115)+
  annotate("segment", x = c(24,72), xend = c(48,96), y = -0.115, yend = -
0.115, color = "grey")

plot_grid(temp)+
  #first facet
  draw_line(x = c(0.054, 0.1252), y = c(0.093, 0.091),color = "grey")+
  draw_line(x = c(0.1252,0.197), y = c(0.093, 0.091))+
  draw_line(x = c(0.197, 0.268), y = c(0.093, 0.091),color = "grey")+
```

```
  draw_line(x = c(0.268, 0.339), y = c(0.093, 0.091))+
  #2nd facet
  draw_line(x = c(0.054+x, 0.1252+x), y = c(0.091, 0.091),color = "grey")+
  draw_line(x = c(0.1252+x,0.197+x), y = c(0.091, 0.091))+
  draw_line(x = c(0.197+x, 0.268+x), y = c(0.091, 0.091),color = "grey")+
  draw_line(x = c(0.268+x, 0.339+x), y = c(0.091, 0.091))+
  #3rd facet
  draw_line(x = c(0.054+y, 0.1252+y), y = c(0.091, 0.091),color = "grey")+
  draw_line(x = c(0.1252+y,0.197+y), y = c(0.091, 0.091))+
  draw_line(x = c(0.197+y, 0.268+y), y = c(0.091, 0.091),color = "grey")+
  draw_line(x = c(0.268+y, 0.339+y), y = c(0.091, 0.091))

## Warning: Removed 6 rows containing missing values (geom_segment).

## Warning: Removed 6 rows containing missing values (geom_segment).

#larvae4b
temp <- ggplot(subset(melted.movement.cpl, time > 9*24 & time < 13*24 &
ExperimentID == "La4b"), aes(x= time, y = value/100))+
  geom_point(size = 0.01)+
  facet_wrap(ExperimentID ~ Animal, ncol = 3, nrow = 24)+
  annotate('rect', xmin=light$lighton,xmax=light$lightoff,ymin=-
Inf,ymax=Inf,fill = "steelblue4", alpha = 0.5)+
  theme_bw(base_size = 8)+
  xlab("zeitgeber time (h)")+
  ylab("mean distance travelled (m/6min)")+
  coord_cartesian(xlim=c(11*24,13*24))+
  scale_y_continuous(limits = c(0,1.55))+
  coord_cartesian(xlim=c(9*24,13*24), ylim = c(0,1.5))+
  scale_x_continuous(breaks = seq(9*24,13.1*24,0.25*24),
                     labels = c("0", "6","12 \n 9 dph", "18", "24",
                                "6", "12 \n 10 dph", "18", "24",
                                "6", "12 \n 11 dph", "18", "24",
                                "6", "12 \n 12 dph", "18", "24"))+
  annotate('rect', xmin=11*24, xmax=11.03*24, ymin=1.54, ymax = 1.5, fill =
'black')+
  annotate('rect', xmin=12.97*24, xmax=13*24, ymin=1.54, ymax = 1.5, fill =
'black')+
  annotate('rect', xmin=11*24, xmax=13*24, ymin=1.54, ymax = 1.52, fill =
'black')+
  annotate("label", x = 12*24, y = 1.52, label = "data fig. 1", size = 2,
label.padding = unit(0.1, "lines"))+
  annotate("segment", x = c(0,48), xend = c(24,72), y = -0.115, yend = -
0.115)+
  annotate("segment", x = c(24,72), xend = c(48,96), y = -0.115, yend = -
0.115, color = "grey")

## Coordinate system already present. Adding new coordinate system, which
will replace the existing one.

plot_grid(temp)+
  #first facet
```

```
  draw_line(x = c(0.054, 0.1252), y = c(0.091, 0.091),color = "grey")+
  draw_line(x = c(0.1252,0.197), y = c(0.091, 0.091))+
  draw_line(x = c(0.197, 0.268), y = c(0.091, 0.091),color = "grey")+
  draw_line(x = c(0.268, 0.339), y = c(0.091, 0.091))+
  #2nd facet
  draw_line(x = c(0.054+x, 0.1252+x), y = c(0.091, 0.091),color = "grey")+
  draw_line(x = c(0.1252+x,0.197+x), y = c(0.091, 0.091))+
  draw_line(x = c(0.197+x, 0.268+x), y = c(0.091, 0.091),color = "grey")+
  draw_line(x = c(0.268+x, 0.339+x), y = c(0.091, 0.091))+
  #3rd facet
  draw_line(x = c(0.054+y, 0.1252+y), y = c(0.091, 0.091),color = "grey")+
  draw_line(x = c(0.1252+y,0.197+y), y = c(0.091, 0.091))+
  draw_line(x = c(0.197+y, 0.268+y), y = c(0.091, 0.091),color = "grey")+
  draw_line(x = c(0.268+y, 0.339+y), y = c(0.091, 0.091))
```

```
## Warning: Removed 6 rows containing missing values (geom_segment).

## Warning: Removed 6 rows containing missing values (geom_segment).
```

```
dev.off()
```

```
## png
##   2
```

```
png("./addition/individual_behaviour_c%03d.png", res = 320, width = 22.75,
height = 14, unit="cm") #activate if separate pdf output is necessary
#larvae5b
temp <- ggplot(melted.mov.compl.larv, aes(x= time, y = value/100))+
  geom_point(size = 0.01)+
  facet_wrap_paginate(ExperimentID ~ Animal, ncol = 3, nrow = 2, page = 3)+
  annotate('rect', xmin=light$lighton,xmax=light$lightoff,ymin=-
Inf,ymax=Inf,fill = "steelblue4", alpha = 0.5)+
  theme_bw(base_size = 8)+
  xlab("zeitgeber time (h)")+
  ylab("mean distance travelled (m/6min)")+
  coord_cartesian(xlim=c(11*24,15*24))+
  scale_x_continuous(breaks = seq(11*24,15.1*24,0.25*24),
                     labels = c("0", "6","12 \n 11 dph", "18", "24",
                                "6", "12 \n 12 dph", "18", "24",
                                "6", "12 \n 13 dph", "18", "24",
                                "6", "12 \n 14 dph", "18", "24"))+
  annotate('rect', xmin=11*24, xmax=11.03*24, ymin=1.54, ymax = 1.5, fill =
'black')+
  annotate('rect', xmin=12.97*24, xmax=13*24, ymin=1.54, ymax = 1.5, fill =
'black')+
  annotate('rect', xmin=11*24, xmax=13*24, ymin=1.54, ymax = 1.52, fill =
'black')+
  annotate("label", x = 12*24, y = 1.52, label = "data fig. 1", size = 2,
label.padding = unit(0.1, "lines"))+
  annotate("segment", x = c(0,48), xend = c(24,72), y = -0.115, yend = -
0.115)+
  annotate("segment", x = c(24,72), xend = c(48,96), y = -0.115, yend = -
```

```
0.115, color = "grey")

plot_grid(temp)+
  #first facet
  draw_line(x = c(0.054, 0.1252), y = c(0.05, 0.05),color = "grey")+
  draw_line(x = c(0.1252,0.197), y = c(0.05, 0.05))+
  draw_line(x = c(0.197, 0.268), y = c(0.05, 0.05),color = "grey")+
  draw_line(x = c(0.268, 0.339), y = c(0.05, 0.05))+
  #2nd facet
  draw_line(x = c(0.054+x, 0.1252+x), y = c(0.05, 0.05),color = "grey")+
  draw_line(x = c(0.1252+x,0.197+x), y = c(0.05, 0.05))+
  draw_line(x = c(0.197+x, 0.268+x), y = c(0.05, 0.05),color = "grey")+
  draw_line(x = c(0.268+x, 0.339+x), y = c(0.05, 0.05))+
  #3rd facet
  draw_line(x = c(0.054+y, 0.1252+y), y = c(0.05, 0.05),color = "grey")+
  draw_line(x = c(0.1252+y,0.197+y), y = c(0.05, 0.05))+
  draw_line(x = c(0.197+y, 0.268+y), y = c(0.05, 0.05),color = "grey")+
  draw_line(x = c(0.268+y, 0.339+y), y = c(0.05, 0.05))

#larvae7
temp <- ggplot(subset(melted.movement.cpl, ExperimentID == "La7" & time >
10*24 & time < 14*24), aes(x= time, y = value/100))+
  geom_point(size = 0.01)+
  facet_wrap(ExperimentID ~ Animal, ncol = 3, nrow = 24)+
  annotate('rect', xmin=light$lighton,xmax=light$lightoff,ymin=-
Inf,ymax=Inf,fill = "steelblue4", alpha = 0.5)+
  theme_bw(base_size = 8)+
  xlab("zeitgeber time (h)")+
  ylab("mean distance travelled (m/6min)")+
  coord_cartesian(xlim=c(10*24,14*24))+
  scale_y_continuous(limits = c(0,1.55))+
  scale_x_continuous(breaks = seq(10*24,14.1*24,0.25*24),
                     labels = c("0", "6","12 \n 10 dph", "18", "24",
                                "6", "12 \n 11 dph", "18", "24",
                                "6", "12 \n 12 dph", "18", "24",
                                "6", "12 \n 13 dph", "18", "24"))+
  annotate('rect', xmin=11*24, xmax=11.03*24, ymin=1.54, ymax = 1.5, fill =
'black')+
  annotate('rect', xmin=12.97*24, xmax=13*24, ymin=1.54, ymax = 1.5, fill =
'black')+
  annotate('rect', xmin=11*24, xmax=13*24, ymin=1.54, ymax = 1.52, fill =
'black')+
  annotate("label", x = 12*24, y = 1.52, label = "data fig. 1", size = 2,
label.padding = unit(0.1, "lines"))+
  annotate("segment", x = c(0,48), xend = c(24,72), y = -0.115, yend = -
0.115)+
  annotate("segment", x = c(24,72), xend = c(48,96), y = -0.115, yend = -
0.115, color = "grey")

plot_grid(temp)+
  #first facet
```

```
  draw_line(x = c(0.054, 0.1252), y = c(0.05, 0.05),color = "grey")+
  draw_line(x = c(0.1252,0.197), y = c(0.05, 0.05))+
  draw_line(x = c(0.197, 0.268), y = c(0.05, 0.05),color = "grey")+
  draw_line(x = c(0.268, 0.339), y = c(0.05, 0.05))+
  #2nd facet
  draw_line(x = c(0.054+x, 0.1252+x), y = c(0.05, 0.05),color = "grey")+
  draw_line(x = c(0.1252+x,0.197+x), y = c(0.05, 0.05))+
  draw_line(x = c(0.197+x, 0.268+x), y = c(0.05, 0.05),color = "grey")+
  draw_line(x = c(0.268+x, 0.339+x), y = c(0.05, 0.05))+
  #3rd facet
  draw_line(x = c(0.054+y, 0.1252+y), y = c(0.05, 0.05),color = "grey")+
  draw_line(x = c(0.1252+y,0.197+y), y = c(0.05, 0.05))+
  draw_line(x = c(0.197+y, 0.268+y), y = c(0.05, 0.05),color = "grey")+
  draw_line(x = c(0.268+y, 0.339+y), y = c(0.05, 0.05))

## Warning: Removed 12 rows containing missing values (geom_segment).

## Warning: Removed 12 rows containing missing values (geom_segment).

#juveniles
subset(melted.movement.cpl,
         ExperimentID == "Ju1" |
         ExperimentID == "Ju2") -> melted.mov.compl.juv

x <- 0.322
y <- 0.645

#juvenile 1
temp <- ggplot(melted.mov.compl.juv, aes(x= time, y = value/100))+
  geom_point(size = 0.01)+
  facet_wrap_paginate(ExperimentID ~ Animal, ncol = 3, nrow = 3, page = 1)+
  annotate('rect', xmin=light$lighton,xmax=light$lightoff,ymin=-
Inf,ymax=Inf,fill = "steelblue4", alpha = 0.5)+
  theme_bw(base_size = 8)+
  xlab("zeitgeber time (h)")+
  ylab("mean distance travelled (m/6min)")+
  ggtitle("juveniles")+
  coord_cartesian(xlim=c(99*24,103*24))+
  scale_x_continuous(breaks = seq(99*24,103*24,0.25*24),
                   labels = c("0", "6","12 \n 99 dph", "18", "24",
                              "6", "12 \n 100 dph", "18", "24",
                              "6", "12 \n 101 dph", "18", "24",
                              "6", "12 \n 102 dph", "18", "24"))+
  annotate('rect', xmin=99*24, xmax=99.03*24, ymin=7.7, ymax = 7.5, fill =
'black')+
  annotate('rect', xmin=100.97*24, xmax=101*24, ymin=7.7, ymax = 7.5, fill =
'black')+
  annotate('rect', xmin=99*24, xmax=101*24, ymin=7.8, ymax = 7.65, fill =
'black')+
  annotate("label", x = 100*24, y = 7.52, label = "data fig. 1", size = 2,
label.padding = unit(0.1, "lines"))+
  annotate("segment", x = c(0,48), xend = c(24,72), y = -0.115, yend = -
```

```
0.115)+
  annotate("segment", x = c(24,72), xend = c(48,96), y = -0.115, yend = -
0.115, color = "grey")

plot_grid(temp)+
  #first facet
  draw_line(x = c(0.0465, 0.1185), y = c(0.05, 0.05),color = "grey")+
  draw_line(x = c(0.1185, 0.1905), y = c(0.05, 0.05))+
  draw_line(x = c(0.1905, 0.2625), y = c(0.05, 0.05),color = "grey")+
  draw_line(x = c(0.2625, 0.3345), y = c(0.05, 0.05))+
  #2nd facet
  draw_line(x = c(0.0465+x, 0.1185+x), y = c(0.05, 0.05),color = "grey")+
  draw_line(x = c(0.1185+x, 0.1905+x), y = c(0.05, 0.05))+
  draw_line(x = c(0.1905+x, 0.2625+x), y = c(0.05, 0.05),color = "grey")+
  draw_line(x = c(0.2625+x, 0.3345+x), y = c(0.05, 0.05))+
  #3rd facet
  draw_line(x = c(0.0465+y, 0.1185+y), y = c(0.05, 0.05),color = "grey")+
  draw_line(x = c(0.1185+y, 0.1905+y), y = c(0.05, 0.05))+
  draw_line(x = c(0.1905+y, 0.2625+y), y = c(0.05, 0.05),color = "grey")+
  draw_line(x = c(0.2625+y, 0.3345+y), y = c(0.05, 0.05))

#juvenile 2
temp <- ggplot(melted.mov.compl.juv, aes(x= time, y = value/100))+
  geom_point(size = 0.01)+
  facet_wrap_paginate(ExperimentID ~ Animal, ncol = 3, nrow = 3, page = 2)+
  annotate('rect', xmin=light$lighton,xmax=light$lightoff,ymin=-
Inf,ymax=Inf,fill = "steelblue4", alpha = 0.5)+
  theme_bw(base_size = 8)+
  xlab("zeitgeber time (h)")+
  ylab("mean distance travelled (m/6min)")+
  ggtitle("juveniles")+
  coord_cartesian(xlim=c(99*24,103*24))+
  scale_x_continuous(breaks = seq(99*24,103*24,0.25*24),
                     labels = c("0", "6","12 \n 99 dph", "18", "24",
                                "6", "12 \n 100 dph", "18", "24",
                                "6", "12 \n 101 dph", "18", "24",
                                "6", "12 \n 102 dph", "18", "24"))+
  annotate('rect', xmin=99*24, xmax=99.03*24, ymin=7.7, ymax = 7.5, fill =
'black')+
  annotate('rect', xmin=100.97*24, xmax=101*24, ymin=7.7, ymax = 7.5, fill =
'black')+
  annotate('rect', xmin=99*24, xmax=101*24, ymin=7.8, ymax = 7.65, fill =
'black')+
  annotate("label", x = 100*24, y = 7.52, label = "data fig. 1", size = 2,
label.padding = unit(0.1, "lines"))+
  annotate("segment", x = c(0,48), xend = c(24,72), y = -0.115, yend = -
0.115)+
  annotate("segment", x = c(24,72), xend = c(48,96), y = -0.115, yend = -
0.115, color = "grey")

plot_grid(temp)+
```

```
  #first facet
  draw_line(x = c(0.0465, 0.1185), y = c(0.05, 0.05),color = "grey")+
  draw_line(x = c(0.1185, 0.1905), y = c(0.05, 0.05))+
  draw_line(x = c(0.1905, 0.2625), y = c(0.05, 0.05),color = "grey")+
  draw_line(x = c(0.2625, 0.3345), y = c(0.05, 0.05))+
  #2nd facet
  draw_line(x = c(0.0465+x, 0.1185+x), y = c(0.05, 0.05),color = "grey")+
  draw_line(x = c(0.1185+x, 0.1905+x), y = c(0.05, 0.05))+
  draw_line(x = c(0.1905+x, 0.2625+x), y = c(0.05, 0.05),color = "grey")+
  draw_line(x = c(0.2625+x, 0.3345+x), y = c(0.05, 0.05))+
  #3rd facet
  draw_line(x = c(0.0465+y, 0.1185+y), y = c(0.05, 0.05),color = "grey")+
  draw_line(x = c(0.1185+y, 0.1905+y), y = c(0.05, 0.05))+
  draw_line(x = c(0.1905+y, 0.2625+y), y = c(0.05, 0.05),color = "grey")+
  draw_line(x = c(0.2625+y, 0.3345+y), y = c(0.05, 0.05))


ggplot(adult_individual)+
  geom_point(aes(x= ZT, y = movement/100))+
  annotate("rect", xmin=-0.5,xmax=0, ymin=-Inf,ymax=Inf,fill = 
"steelblue4",alpha = 0.5)+
  annotate("rect", xmin=0,xmax=0.25, ymin=-Inf,ymax=Inf,fill = 
"steelblue4",alpha = 0.3)+
  annotate("rect", xmin=13.5,xmax=14, ymin=-Inf,ymax=Inf, fill = 
"steelblue4",alpha = 0.3)+
  annotate("rect", xmin=14,xmax=24, ymin=-Inf,ymax=Inf, fill = 
"steelblue4",alpha = 0.5)+
  facet_wrap(variable ~ plot, ncol = 2, scales = "free_y")+
  scale_x_continuous(limits=c(NA,24.1),breaks = c(seq(0,24,3)))+
  xlab("zeitgeber time (h)")+
  ylab("mean distance travelled (m/1min)")+
  theme_bw(base_size = 8)+
  ggtitle("adults")

dev.off()

## png 
##   2
```

7 Luciferase assay

We imported the data into Excel by using the "Import and Analysis" macro (S. Kay, Scripps Research Institute) and exported it as txt for import into R.

```
#load data
luciferase <- read.delim2("./data/luciferase.txt")

#exclude irrelevant data
luciferase[,c(1,10:17,18:25,34:41,66:73,82:89,58:65)] -> luciferase
```

```r
#make a new data frame by calculating mean and standard deviation for the different reporter constructs
as.data.frame(cbind(
  ZT = luciferase[,1],
  EAO_control = apply(luciferase[,2:9],1,mean),
  EAO_control_sd = apply(luciferase[,2:9],1,sd),
  EAO_controlb = apply(luciferase[,2:9],1,mean),
  EAO_controlb_sd = apply(luciferase[,2:9],1,sd),
  clownfish_per1b = apply(luciferase[,10:17],1,mean),
  clownfish_per1b_sd = apply(luciferase[,10:17],1,sd),
  clownfish_dbox = apply(luciferase[,18:25],1,mean),
  clownfish_dbox_sd = apply(luciferase[,18:25],1,sd),
  PAC2_per1b = apply(luciferase[,26:33],1,mean),
  PAC2_per1b_sd = apply(luciferase[,26:33],1,sd),
  PAC2_dbox = apply(luciferase[,34:41],1,mean),
  PAC2_dbox_sd = apply(luciferase[,34:41],1,sd),
  PAC2_controla = apply(luciferase[,42:48],1,mean),
  PAC2_controla_sd = apply(luciferase[,42:48],1,sd),
  PAC2_control = apply(luciferase[,42:48],1,mean),
  PAC2_control_sd = apply(luciferase[,42:48],1,sd)
))-> data

#melt the data frame into long format and add category
melt(data[,c(1,seq(2,17,2))],id.vars = 'ZT') -> melted.data
melt(data[,c(1,seq(3,17,2))],id.vars = 'ZT') -> melted.data.sd

as.data.frame(cbind(
  melted.data,
  sd = melted.data.sd[,3],
  category = c(rep("zfPer1b-Luc",172),rep("D-box[cry1a]*-
Luc",172),rep("zfPer1b-Luc",172),rep("D-box[cry1a]*-Luc",172),rep("zfPer1b-
Luc",172),rep("D-box[cry1a]*-Luc",172),rep("zfPer1b-Luc",172),rep("D-
box[cry1a]*-Luc",172))
)) -> melted.data

#choose colours
cbPalette <-
c("grey50","grey50","coral","coral","blue","blue","grey50","grey50")

y <- 1

#save data frame with light on/off
light <-  data.frame(
  lightoff = c(12,36,60,84),
  lighton =  c(24,48,72,120)
)

#sort
levels(melted.data$category) <- c("D-box[cry1a]*-Luc","zfPer1b-Luc")
```

```r
#make the plot
ggplot(melted.data,aes(x = ZT, y=value/y))+
  annotate('rect', xmin = light$lightoff, xmax = light$lighton, ymin = -Inf,
ymax = Inf,fill = "steelblue4",alpha = 0.5)+
  geom_point(aes(color = variable),size=0.25)+
  geom_line(aes(color = variable),size = 0.1)+
  geom_errorbar(aes(ymin= (value - sd)/y, ymax = (value + sd)/y), size=.01)+
  theme_bw(base_size = 8)+
  geom_rect(data = data.frame(category = "zfPer1b-Luc"), aes(xmin = 12, xmax
= 24, ymin = -1000/y, ymax = -700/y), inherit.aes = FALSE,fill="black")+
  geom_rect(data = data.frame(category = "zfPer1b-Luc"), aes(xmin = 36, xmax
= 48, ymin = -1000/y, ymax = -700/y), inherit.aes = FALSE,fill="black")+
  geom_rect(data = data.frame(category = "zfPer1b-Luc"), aes(xmin = 60, xmax
= 72, ymin = -1000/y, ymax = -700/y), inherit.aes = FALSE,fill="black")+
  geom_rect(data = data.frame(category = "zfPer1b-Luc"), aes(xmin = 84, xmax
= 120, ymin = -1000/y, ymax = -700/y), inherit.aes = FALSE,fill="black")+
  geom_rect(data = data.frame(category = "D-box[cry1a]*-Luc"), aes(xmin = 12,
xmax = 24, ymin = -600/y, ymax = -540/y), inherit.aes = FALSE,fill="black")+
  geom_rect(data = data.frame(category = "D-box[cry1a]*-Luc"), aes(xmin = 36,
xmax = 48, ymin = -600/y, ymax = -540/y), inherit.aes = FALSE,fill="black")+
  geom_rect(data = data.frame(category = "D-box[cry1a]*-Luc"), aes(xmin = 60,
xmax = 72, ymin = -600/y, ymax = -540/y), inherit.aes = FALSE,fill="black")+
  geom_rect(data = data.frame(category = "D-box[cry1a]*-Luc"), aes(xmin = 84,
xmax = 120, ymin = -600/y, ymax = -540/y), inherit.aes = FALSE,fill="black")+
  facet_wrap(category ~ .,scales = "free",ncol = 1, nrow =2, labeller =
label_parsed)+
  scale_x_continuous(breaks = seq(0,144,24), limits = c(0,120),labels =
c(0,24,0,24,0,24,0))+
  xlab("zeitgeber time (h)")+
  ylab("bioluminescence (counts/s)")+
  theme(legend.position="none")+
  scale_colour_manual(values=cbPalette)+
  scale_y_continuous(labels = comma)

## Warning: Removed 216 rows containing missing values (geom_point).

## Warning: Removed 216 row(s) containing missing values (geom_path).
```

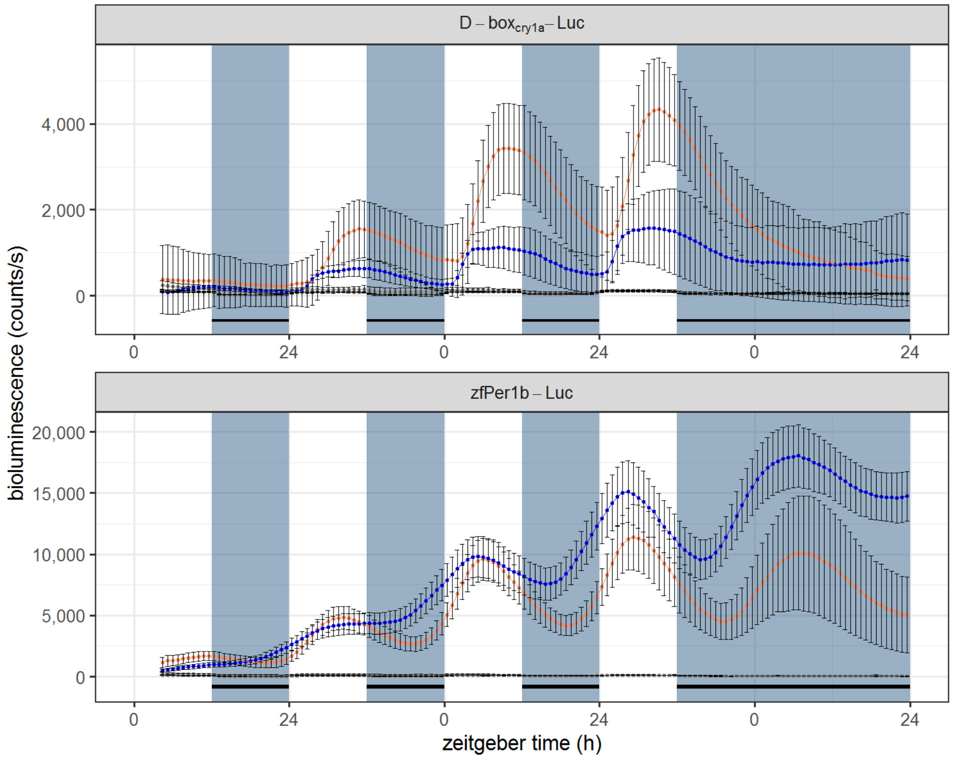


```
ggsave("./results/luciferase.pdf",width = 9, height = 8, units = "cm")

## Warning: Removed 216 rows containing missing values (geom_point).

## Warning: Removed 216 row(s) containing missing values (geom_path).
```
